# A near‐complete genome sequence of mungbean (*Vigna radiata* L.) provides key insights into the modern breeding program

**DOI:** 10.1002/tpg2.20121

**Published:** 2021-07-18

**Authors:** Jungmin Ha, Dani Satyawan, Haneul Jeong, Eunsoo Lee, Kang‐Heum Cho, Moon Young Kim, Suk‐Ha Lee

**Affiliations:** ^1^ Dep. of Plant Science Gangneung‐Wonju National Univ. Gangneung Republic of Korea; ^2^ Indonesian Center for Agricultural Biotechnology and Genetic Resources Research and Development (ICABIOGRAD‐IAARD) Jl. Tentara Pelajar No. 3A Bogor 16111 Indonesia; ^3^ Dep. of Agriculture, Forestry and Bioresources and Research Institute of Agriculture and Life Sciences Seoul National Univ. Seoul 08826 Republic of Korea; ^4^ Plant Genomics and Breeding Institute Seoul National Univ. Seoul 08826 Republic of Korea

## Abstract

Mungbean (*Vigna radiata* L.), a fast‐growing legume species, is an important source of carbohydrates and proteins in developing countries of Asia. Here, we constructed a near‐complete genome sequence of mungbean with a scaffold N50 value of 5.2 Mb and only a 0.4% gap, with a total scaffold size of 475 Mb. We identified several misassembled pseudomolecules (Chr03, Chr04, Chr05, and Chr08) in the previous draft assembly; Chr03, Chr04, and Chr08 were assembled into one chromosome, and Chr05 was broken into two chromosomes in the improved reference genome assembly, thus providing more accurate linkage information to breeders. Additionally, using an ultra‐high‐resolution linkage map constructed based on resequencing data, we identified several quantitative trait loci (QTLs) and the underlying candidate genes affecting synchronous pod maturity (SPM). Mungbean homologs of two soybean ([*Glycine max* (L.) Merr.] flowering genes, *E3* (*phytochrome A*) and *J* (*early flowering 3*), were identified as candidate genes for the QTLs, and the candidate genes for plant height, node number, and SPM showed critical nucleotide substitutions between the reference cultivar and other genotypes (landraces and wild accessions). Based on the analysis of genetic diversity among 276 accessions collected from 23 countries, we identified 36 selective sweep regions and observed that the overall genetic diversity of cultivars decreased to 30% of that in wild accessions postdomestication. The near‐complete genome sequence of mungbean represents an important resource for genome‐assisted improvement in the mungbean breeding program.

## Core Ideas

- A near‐complete reference genome sequence of mungbean was constructed.
- Misassembled pseudomolecules in the previous assembly were corrected.
- QTLs were identified based on ultra‐high‐resolution genetic map constructed by resequencing.
- Genetic factors affecting synchronous pod maturity were identified.
- Genetic diversity among 276 accessions from 23 countries were analyzed.

**Abbreviations**
Chr: chromosome
Fst: fixation index
GBS: genotyping‐by‐sequencing
GO: gene ontology
LD: linkage disequilibrium
LOD: logarithm of odds
PC: principle component
PCA: principal component analysis
PVE: percentage of variance explained
QTL: quantitative trait locus
RIL: recombinant inbred line
SNP: single nucleotide polymorphism
SPM: synchronous pod maturity
UTR: untranslated region
XP‐CLR: cross‐population composite likelihood ratio

## 1 INTRODUCTION

Mungbean (*Vigna radiata* L.) is an annual self‐pollinated species belonging to the papilionoid subfamily of the Fabaceae (2*n* = 2*x* = 22) (Kang et al., 2014). It is a versatile legume crop that is widely consumed in Asia. As a protein source, the production of mungbean seed proteins requires fewer resources than that of animal proteins. Moreover, mungbean is an excellent source of folate and other vitamins when consumed as vegetable sprouts (Shohag et al., 2012). The ability of mungbean to host nitrogen‐fixing bacteria improves soil fertility and reduces the greenhouse gas emission footprint commonly found in agricultural production (Nair et al., 2012). Because several *Vigna* species have been produced and consumed as economically important legume species in Asia, including adzuki bean [*V. angularis* (Willd.) Ohwi & Ohashi], cowpea [*V. unguiculata* (L.) Walp.], moth bean [*V. aconitifolia* (Jacq.) Marechal], rice bean [*V. umbellate* (Thunb.) Ohwi & H. Ohashi], and yard‐long bean [*V. sesquipedalis* (L.) Walp. ssp. *sesquipedalis* (L.) Verdc.], high‐quality genomic information of *Vigna* species is essential for improving the crop's quality in Asia.

Despite the effect of mungbean on the diet of a sizable proportion of the world population, genomic information for molecular breeding in this species is lacking compared with other legumes such as soybean [*Glycine max* (L.) Merr.] and chickpea (*Cicer arietinum* L.) (Schmutz et al., 2010; Varshney et al., 2013). A draft mungbean genome sequence constructed previously covers 80% of the estimated genome size but still contains gaps in 7.2% of the total scaffold, and only 239 out of 2,748 scaffolds have been mapped to pseudochromosomes (Kang et al., 2014). This draft reference genome has helped breeders to conduct quantitative trait locus (QTL) mapping or map‐based cloning, and to transfer QTL information from soybean through translational genomics (Kim et al., 2015). However, high‐quality reference sequence map data are crucial for forward genetics approaches to determine the exact location of genes and mutations underlying desirable phenotypes.

Mungbean is characterized by nonsynchronous pod maturity, i.e., continued flowering and pod production. Therefore, a single plant must be harvested multiple times to reduce yield loss. However, this costs extra labor and time. Additionally, each harvest must be conducted carefully to avoid damage to plants, which makes mechanical harvesting difficult. Although determinate growth habit has been characterized in mungbean (Li et al., 2018), the genetic basis of SPM remains unresolved. Synchronous pod maturity (SPM) is a complex trait affected by multiple factors, such as flowering time, maturity time, inflorescence structure, and plant architecture (Ha et al., 2020a, 2020b). Quantitative trait locus analyses of these factors in mungbean are limited, especially those that utilize high‐density single nucleotide polymorphism (SNP) markers. Only a few traits, such as flowering and disease resistance in mungbean, have been studied using this method (Hwang et al., 2017; Schafleitner et al., 2016).

Sequencing platforms capable of producing long reads have been utilized to reduce gaps and improve coverage in genome assemblies (English et al., 2012). Here, we report a near‐complete genome sequence of mungbean using long sequence reads as well as an ultra‐high‐resolution genetic map obtained from whole genome resequencing data of a genetic mapping population of mungbean. We took advantage of the resulting combination of a high‐quality reference genome and a dense linkage map to identify QTLs and the underlying candidate genes associated with agronomically important traits that affect SPM. We also examined the genetic diversity of 275 wild and cultivated mungbean accessions. Our results provide high‐quality genomic information for the improvement of all *Vigna* species, especially mungbean.

**Core Ideas**

- A near‐complete reference genome sequence of mungbean was constructed.
- Misassembled pseudomolecules in the previous assembly were corrected.
- QTLs were identified based on an ultra‐high‐resolution genetic map constructed by resequencing.
- Genetic factors affecting synchronous pod maturity were identified.
- Genetic diversity among 276 accessions from 23 countries were analyzed.

## 2 MATERIALS AND METHODS

### 2.1 Plant materials and phenotyping

One hundred eighty‐seven F_10_ plants of a recombinant inbred line (RIL) population were generated from F_2_ seed using single‐seed descent derived from a cross between *V. radiata* cultivar VC1973A and landrace V2984. The RIL population were planted at the Seoul National University Experimental Farm in Suwon, Korea (37°16′12.7″ N; 126°59′19.2″ E). To analyze QTLs, various agronomic traits were measured including plant height, node number, branch number, flower initiation, and SPM. Plant height, node number, and branch number were measured when the first flower appeared and vegetative growth stopped. The number of branches with two or more nodes generated on the main stem were evaluated. To measure flower initiation, the date of the appearance of the first flower was recorded for each line. To measure SPM, 10 plants of each line were harvested eight times at weekly intervals between 26 Aug. and 14 Oct. 2016, and the sum of the two highest grain yields from two consecutive weeks was divided by the total yield (Ha et al., 2020a). Theoretically, a SPM value of 1 indicates that all pods mature at the same time.

### 2.2 Genome assembly and annotation

*Vigna radiata* var. *radiata*, the pure line VC1973A, was sequenced for genome assembly using the PacBio RS II platform and data were deposited in the National Center for Biotechnology Information sequence read archive database under the accession number srrs9994113 (Supplemental Table S1). Raw sequence reads were error‐corrected and trimmed by Canu (Version 1.0) (Koren et al., 2017). The corrected reads were assembled into contigs using Falcon (Version 0.3.0) (Chin et al., 2016). The contigs were scaffolded with Illumina mate‐pair reads with library sizes of 5, 10, and 40 kb using SSPACE (Version 3.0) (Boetzer et al., 2011). The scaffolds were anchored to pseudochromosomes using two genetic maps by ALLMAPS (Tang et al., 2015). Gaps in the superscaffolds were filled by Illumina short reads using Gapfiller (Version 1.10) (Boetzer & Pirovano, 2012). Illumina reads were obtained from the National Center for Biotechnology Information under the accession number JJMO00000000 (Kang et al., 2014). The assembled genome was annotated via the pipeline described by Ha et al. (2019) based on transcriptome data from JJMO00000000. To examine the mungbean genome assembly, core eukaryotic genes from CEGMA (Version 2.5) (Parra et al., 2007) were mapped to the assembly using BLAST (Version 2.2.31) (Camacho et al., 2009). In addition, single‐copy orthologous genes of eukatyota and embryophyta selected from BUSCO (Simão et al., 2015) were mapped to the mungbean genome assembly. Default settings were used to run software programs.

### 2.3 Genetic map construction and QTL analysis

Illumina short reads (SRR10083737–SRR10083923) generated from 187 RILs with the Illumina HiSeq 4000 platform were used for SNP analysis (Supplemental Table S3). Reads were mapped to scaffolds using BWA (Version 0.7.15) (Li, 2013). The SNPs were called from the mapped reads and filtered using SAMtools (Version 1.3) (Li et al., 2009) using the following criteria: mapping quality ≥ 30, depth ≥ 5, heterozygosity ≤ 10, and missing data ≤ 12. The detected SNPs were used to construct a genetic map using JoinMap (Version 4.1) (Van Ooijen, 2006). To construct robust pseudochromosomes, a secondary genetic map was constructed (Supplemental Table S5) using the same method but with a different set of SNP markers with mapping quality ≥30, depth ≥5, heterozygosity ≤0, and missing data ≤18.

The QTL analysis was conducted using the primary genetic map composed of 8,966 SNP markers (Supplemental Table S4). The QTL positions were identified by inclusive composite interval mapping for additive QTLs using QTL ICIMapping (Version 4.0.6) (Meng et al., 2015). To determine the significance thresholds for QTLs, a logarithm of odds (LOD) score was calculated with 1,000 replications of a permutation test, with 99% confidence. Two significant QTLs, with the highest LOD scores, were reported per trait.

### 2.4 Genome sequence comparison

A total of 100 evenly distributed SNP markers per chromosome from the previous mungbean genome assembly were mapped to the newly assembled chromosomes using ±100 bp of the sequence flanking each marker using BLAST. Only the markers with 90% or higher identity were used to compare the two genome assemblies. Genes from the previous mungbean assembly were aligned to the genome of close relatives including *V. angularis* (Kang et al., 2015), *Medicago truncatula* Gaertn. (Tang et al., 2014), and *C. arietinum* (Varshney et al., 2013) using BLAST. To study synteny and collinearity between mungbean and other species, BLAST results were analyzed using MCScanX (Wang et al., 2012). Syntenic blocks containing ≥20 genes were used to compare the two species.

### 2.5 Genotyping‐by‐sequencing and variant calling

A total of 275 mungbean accessions, including 233 cultivars and 42 wild accessions, and one accession of *V. glabrescens* Maréchal, Mascherpa & Stainier (outgroup), collected from 23 countries (Supplemental Table S9), were grown and used for DNA extraction. Library construction for genotyping‐by‐sequencing (GBS) was performed as described previously (Elshire et al., 2011). Sequence reads generated using the Illumina HiSeq 2000 platform were mapped to the mungbean reference genome using BWA (Li, 2013), and variants were identified using bcftools call command in SAMtools (Li et al., 2009). The variants were filtered using vcftools (Danecek et al., 2011) and SnpEff (Cingolani et al., 2012). Only variants with a quality score greater than 30, sequencing depth of more than two reads per site, and a minimum of four reads per heterozygous variant were used for further analysis. The circular plot for variant number across chromosomes was drawn in Circos (Krzywinski et al., 2009).

### 2.6 Phylogenetic and population structure analyses

A neighbor‐joining tree was constructed based on the dissimilarity matrix generated using filtered SNPs with DARwin6 (Perrier & Jacquemoud‐Collet, 2015). The tree was drawn and edited using Figtree (Version 1.4.2) (http://tree.bio.ed.ac.uk) and iTOL (Letunic & Bork, 2006). Population structure analysis was performed using a random sample of 1,000 SNPs with STRUCTURE (Version 2.3.4) (Pritchard et al., 2000). The number of clusters was set from *K* = 2 to *K* = 7, with 50,000 burn‐in and 100,000 Markov Chain Monte Carlo replications. The optimal *K* value was identified according to the methods of Evanno et al. (2005). To observe the dominant cluster in each country of origin, the population structure data of each accession were grouped according to the country, and average *q*‐values (the proportion of the SNPs from each group) were calculated. Principle component analysis (PCA) was performed using GenAlEx (Version 6.5) (Peakall & Smouse, 2006).

### 2.7 Linkage disequilibrium profiling and identification of selective sweep regions

Linkage disequilibrium (LD) was calculated based on *R* ^2^ statistics (Hill & Robertson, 1968) implemented in vcftools (Danecek et al., 2011), and the calculation was performed separately for wild and cultivated mungbean accessions. The *R* ^2^ values between markers were then binned and averaged for each 10,000‐bp increment. The LD decay cutoff was set at 0.2, and the exact base pair position for the cutoff was calculated based on the trendline equation.

The fixation index (Fst) and cross‐population composite likelihood ratio (XP‐CLR) were calculated to identify selective sweep regions (Chen et al., 2010). A 100‐kb window was analyzed for XP‐CLR, and Fst analysis was based on markers. Segments with the top 5% values from each program were searched, and intervals that overlapped with each other were identified as selective sweep regions. Gene ontology (GO) analysis was performed by submitting orthologous *Arabidopsis* genes, identified by blastp, to BiNGO (Maere et al., 2005). Soybean QTLs were searched in regions homologous to mungbean selective sweep regions based on syntenic relationship identified using MCScanX (Wang et al., 2012).

## 3 RESULTS

### 3.1 Mungbean genome assembly and genetic map construction

The genome of mungbean cultivar VC1973A was sequenced using the PacBio platform at 68.21X coverage, and the resulting long sequence reads were de novo assembled into a near‐complete reference genome (Supplemental Table S1). The primary contigs consisted of 1,511 sequences with an N50 value of 2.8 Mb, which is significantly fewer and longer compared with the 25,922 contigs with N50 value of 42 kb in the previous draft assembly (Kang et al., 2014). The total length of contigs also increased from 431 to 473 Mb (Table 1; Supplemental Table S2), which represents 87.1% of the predicted genome size of 543 Mb (Arumuganathan & Earle, 1991). These contigs were then used to generate 487 scaffolds with a scaffold N50 value of 5.2 Mb, a notable improvement from the 2,748 scaffolds with an N50 value of 1.5 Mb in the previous assembly. The new scaffolds also contained 0.4% gaps compared with the 7.2% gaps found in the previous draft assembly.

**TABLE 1:** Summary of VC1973A assembly and annotation

Assembly and annotation	N50	Total length	No.
	Mbp	Mbp	
Contigs	2.8	473.4	1,511
Scaffolds	5.2	475.4	487
Superscaffolds	47.1	475.7	388
Ratio of Ns in scaffolds (%)	0.40		
GC contents (%)	33.27		
No. of high‐confidence genes^a^	30,958		
248 core eukaryotic genes	243		
Interproscan/GO	27,296		
KEGG	6,694		
Swissprot	23,800		
TrEMBL	29,509		
NR/NT	29,552		
Total annotated high‐confidence genes	29,792		
Total unannotated high‐confidence genes	1,166		

^a^ High‐confidence genes were mapped against publicly available databases, including 248 CEGs, Interproscan/GO, KEGG, Swissprot, TrEMBL, and NR/NT.

To assemble the scaffolds into pseudochromosomes, a new genetic linkage map was constructed using Illumina resequencing data of 187 RILs sequenced at an average coverage of 12.15X (Supplemental Tables S3 and S4). A high‐resolution genetic map consisting of 8,966 markers was used to place 110 scaffolds into 11 pseudochromosomes (Supplemental Table S5; Supplemental Figure S1). Gaps were further filled and assembly errors (0.02% error rate) were corrected using Illumina short reads to generate the final pseudochromosomes of 476 Mb. While the previous draft assembly contained only 22,427 predicted genes, the new assembly contained 30,958 high‐confidence genes, of which 29,792 (96%) were annotated (Kang et al., 2014). The predicted genes showed ∼98% identity to 248 core eukaryotic genes (Table 1).

### 3.2 Improvement of the mungbean reference genome sequence

**FIGURE 1:**
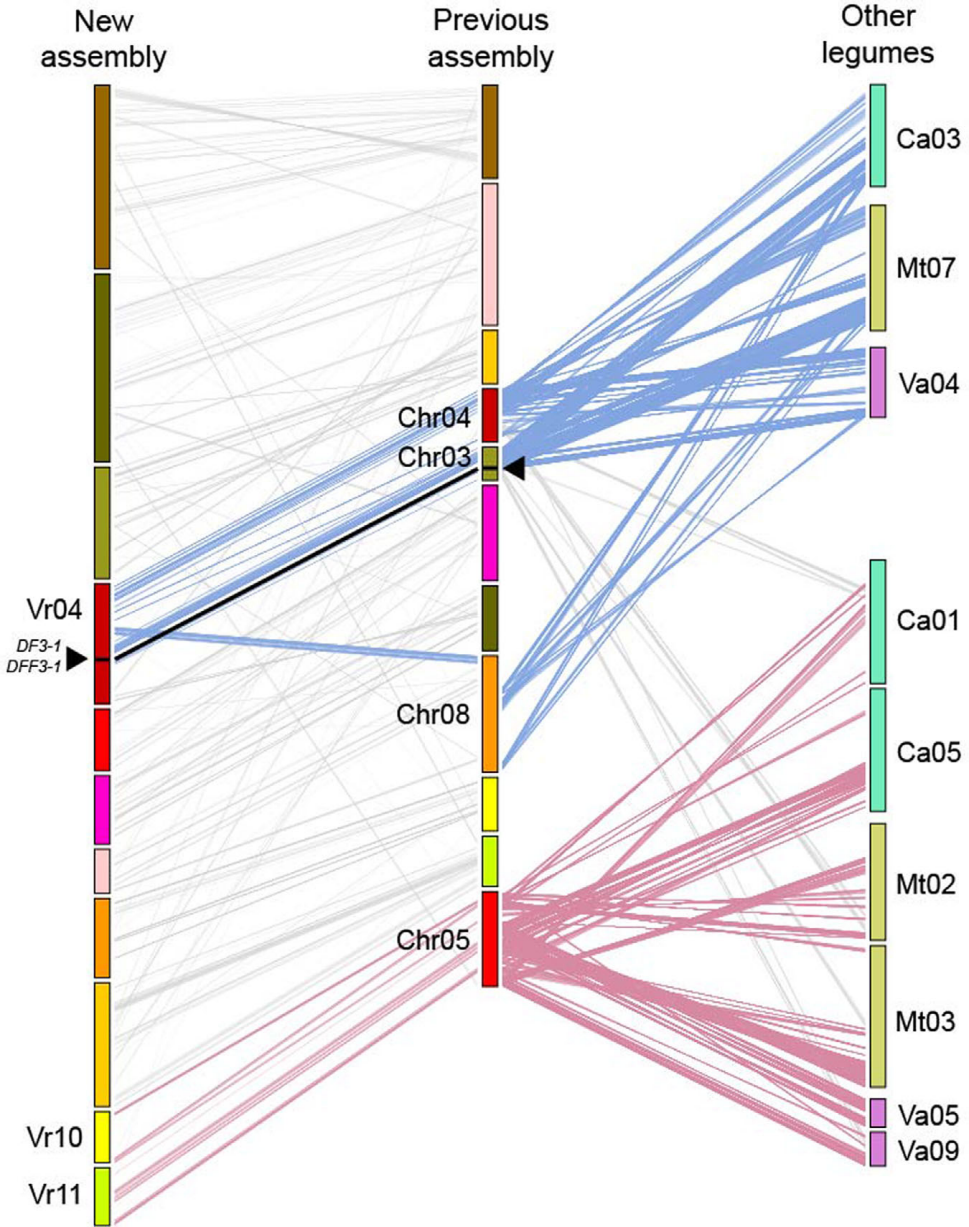
Syntenic relationship between *Vigna radiata* and its close relatives. Evenly distributed 100 markers per chromosome of the previous assembly (center) were mapped to the new assembly (left column). Some markers from chromosome (Chr) 03, Chr04, and Chr08 of the previous assembly were mapped to Vr04 of the new assembly as indicated by blue lines. Markers from Chr05 of the previous assembly that were mapped to Vr10 and Vr11 of the new assembly are indicated by red lines. Gene‐based synteny analysis between mungbean and *Cicer aretinum*, *Medicago truncatula*, and *V. angularis* (right column) shows that these close relatives have the same syntenic pattern with the new mungbean assembly. Black arrow head indicates the location of *DF3‐1* and *DFF3‐1*

Using data from the previous draft assembly, 100 evenly distributed SNP markers per chromosome (Chr) were mapped to the newly assembled chromosomes. Out of 1,100 markers on 11 chromosomes, 959 markers were mapped with 90% or higher identity (Supplemental Table S6). A total of 91, 79, and 41 markers from Chr03, Chr04, and Chr08, respectively, of the previous assembly were mapped to Vr04 in the new assembly (Figure 1).

Approximately 35% of the 85 markers on Chr05 of the previous assembly were mapped to Vr10, while 54% of those markers are mapped to Vr11 in the new assembly (Figure 1; Supplemental Table S6). To resolve the discrepancies between the old and new genome assemblies, syntenic relationships of genes on Chr03, Chr04, Chr05, and Chr08 in the old genome assembly were analyzed in the close relatives of mungbean, including chick pea (*C. arietinum*), barrel clover (*M. truncatula*), and adzuki bean (*V. angularis*). A large proportion of genes on Chr03, Chr04, and Chr08 in the old mungbean assembly converged to a single chromosome in the three related species: in Chr03 of *C. arietinum*, Chr07 of *M. truncatula*, and Chr04 of *V. angularis* (Figure 1; Supplemental Table S7). On the other hand, genes on Chr05 in the old mungbean assembly mapped to two different chromosomes in the related species (Chr01 and Chr05 of *C. aretinum*, Chr02 and Chr03 of *M. truncatula*, and Chr05 and Chr09 of *V. angularis*) (Figure 1; Supplemental Table S7). These syntenic relationships between mungbean and its close relatives indicate that the new genome assembly is more reliable than the previous assembly.

In a previous QTL study, *days‐to‐flowering3‐1* (*DF3‐1*) and *days‐to‐first‐flowering3‐1* (*DFF3‐1*) were mapped to Chr03, one of the misassembled pseudomolecules in the previous draft (Hwang et al., 2017). Such misassemblies on Chr03, Chr04, Chr05, and Chr08 in the previous assembly were corrected in the new assembly. Thus, the new assembly provides bonafide linkage information to breeders, which is crucial for marker‐assisted breeding and map‐based cloning.

### 3.3 QTL analysis of agronomically important traits affecting SPM

**FIGURE 2:**
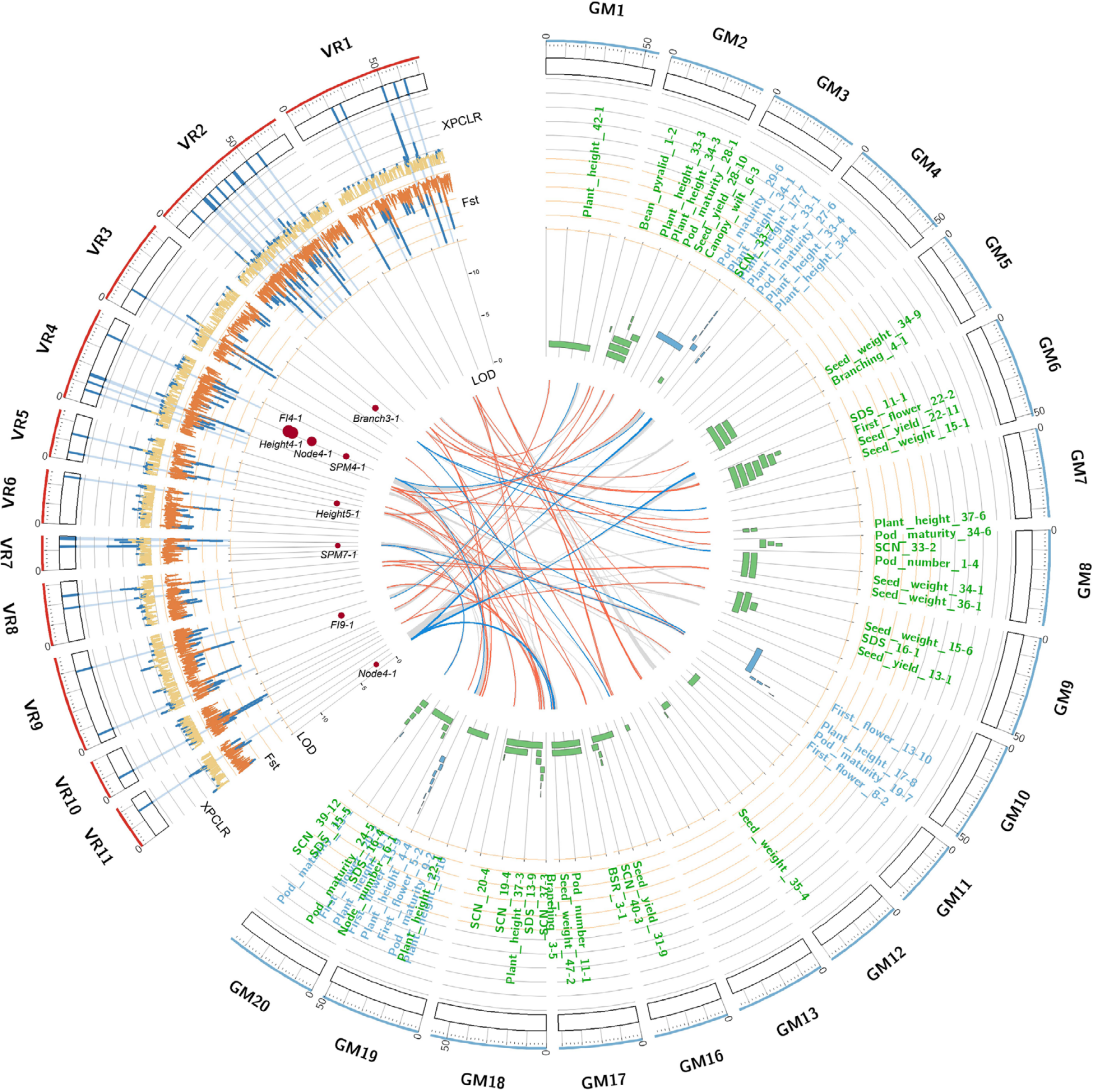
Distribution of genetic regions associated with domestication and agronomically important traits. The cross‐population composite likelihood ratio (XP‐CLR) (yellow lines) and fixation index (Fst) (orange lines) values between wild and cultivated accessions were indicated in the outer circle across mungbean chromosomes indicated by red bars. Blue peaks are the 5% highest values in XP‐CLR and Fst, which indicate selective sweep regions highlighted by blue on mungbean chromosomes. The middle circle shows loci and logarithm of odds (LOD) score of Vr quantitative trait loci (QTLs) identified in this study. The inner circle represents synteny between mungbean genomic regions of selective sweep (red) and QTLs (blue), and soybean genomic regions. The soybean QTLs reported on the syntenic regions of selective sweeps and VrQTLs were indicated by green and blue bars, respectively, on soybean chromosomes indicated by blue bars

Synchronous pod maturity is influenced by multiple traits that contribute to the plant architecture of mungbean. Mungbean develops indeterminate inflorescences from each node on branches and the upper stem, showing determinate and indeterminate vegetative growth in cultivated and wild species, respectively. This is defined as compound raceme (Benlloch et al., 2015). Therefore, QTLs regulating plant height, node number, branch number, flower initiation, and SPM (Supplemental Figure S2) were identified based on a genetic map constructed using resequencing data (Table 2; Supplemental Figures S3 and S4; Figure 2). The QTLs for plant height were detected on Vr04 (*Height4‐1*) and Vr05 (*Height5‐1*), which explained 23.9% and 6.2% of the variance in plant height, respectively. The QTLs for flower initiation were identified on Vr04 (*FI4‐1*) and Vr09 (*FI9‐1*), with 24.1 and 6.4 percentage of variance explained (PVE), respectively. One QTL affecting branch number was detected on Vr03 (*Branch3‐1*), with 11.1 PVE. The QTLs affecting node number were identified on Vr04 (*Node4‐1*) and Vr11 (*Node11‐1*), with 20.0 and 6.3 PVE, respectively. Two QTLs for SPM were detected on Vr04 (*SPM4‐1*) and Vr07 (*SPM7‐1*), with 10.3 and 6.8 PVE, respectively. On Vr04, *Height4‐1*, *Node4‐1*, and *SPM4‐1* were identified within the same marker interval, and *FI4‐1* was directly adjacent to them, sharing one genetic marker (29,615,281 bp) (Supplemental Figure S5). These four QTLs are located in a 235.7‐kb interval that showed syntenic relationship with three genomic regions on chromosomes 3, 10, and 19 in soybean (Gm03, Gm10, and Gm19, respectively) (Supplemental Figure S5). Multiple QTLs were reported previously in those genomic regions in soybean: *plant height 26‐17* (Sun et al., 2006) on Gm03; *plant height 4‐4* (Lee et al., 1996), *first flower 16‐4* (Khan et al., 2008), and *first flower 20‐2* (Funatsuki et al., 2005) on Gm19; and *plant height 17‐8* (Kabelka et al., 2004) and *pod maturity 19‐7* (Guzman et al., 2007) on Gm10. Moreover, QTLs *DF3‐1* and *DFF3‐1* were reported on Chr03 in the previous mungbean genome assembly (Hwang et al., 2017), which corresponds to Vr04 in the new assembly. The chromosome interval containing *FI4‐1*, *Height4‐1*, *Node4‐1*, and *SPM4‐1* overlapped with *DF3‐1* and *DFF3‐*1, thus narrowing down the QTL region for flowering and aiding the identification of a new candidate gene for flowering in mungbean. In addition, the *Dt1* locus, which regulates determinant growth habit in soybean, was located around the syntenic region on Gm19 (Tian et al., 2010).

**TABLE 2:** Identification of quantitative trait loci with major effects on agronomic traits

Trait	Locus	Chr	Left marker position	Right marker position	LOD	PVE %	Add	No. of genes	Candidate genes	Soybean flowering locus
Plant height	*Height4‐1*	4	29,846,687	29,615,281	14.2	24.9	−5.95	31	*PHYA*	*E3*
	*Height5‐1*	5	4,812,045	4,811,991	4.0	6.2	2.97	1	*kinase*	
Flower initiation	*FI4‐1*	4	29,615,280	29,610,944	14.8	24.1	−0.92	2	*Bonsai*	
	*FI9‐1*	9	21,846,626	21,872,313	4.5	6.4	−0.48	6	*R*‐genes	
No. of branches	*Branch3‐1*	3	41,265,068	41,618,277	4.8	11.1	0.27	44	*ERF4*	
No. of nodes	*Node4‐1*	4	29,846,687	29,615,281	10.8	20.0	−0.36	31	*PHYA*	*E3*
	*Node11‐1*	11	7,961,123	5,573,387	3.1	6.3	0.20	286	*ELF3*	*J*
Synchronous pod maturity	*SPM4‐1*	4	29,846,687	29,615,281	4.8	10.3	0.03	31	*PHYA*	*E3*
	*SPM7‐1*	7	11,619,769	11,535,175	3.2	6.8	0.02	6	*PKp1*	

*Note*. Add, additive; Chr, chromosome; LOD, logarithm of odds; PVE, percentage of variance explained.

### 3.4 Identification of candidate genes underlying QTLs

**FIGURE 3:**
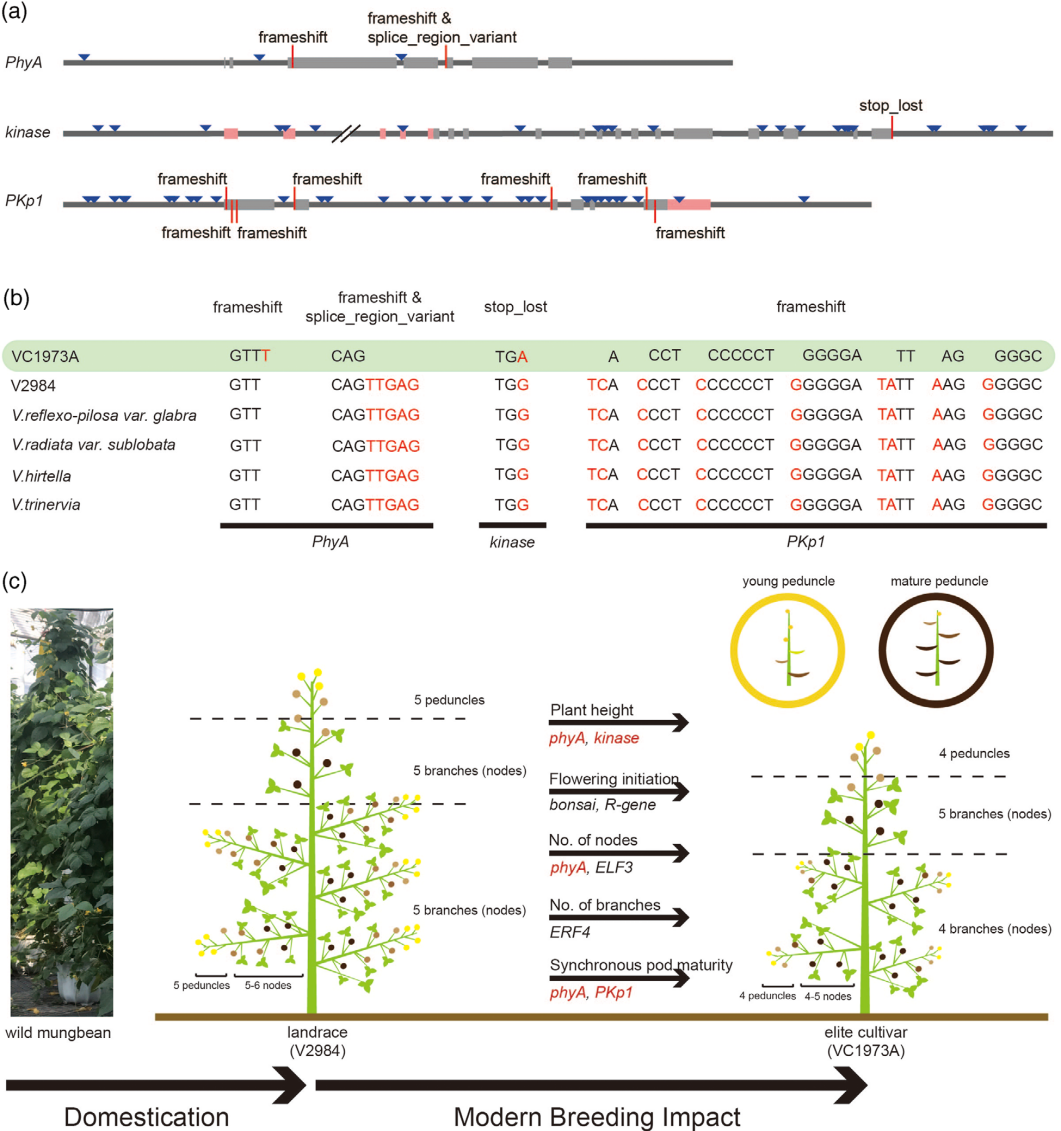
Genetic and morphological differences among VC1973A, V2984, and wild mungbeans. (a) The location of single nucleotide polymorphisms (SNPs) and insertions and deletions resulting in the losses of stop codons and frameshifts. Grey and pink rectangles indicate exons and untranslated regions. Blue arrow heads indicate SNPs. (b) Sequence comparison among VC1973A, V2984, and wild mungbean species. VC1973A, the mungbean reference cultivar, is highlighted in green. (c) Morphological changes among wild mungbean, landrace, and elite cultivar. VC1973A is shorter and has fewer nodes and branches than V2984. The candidate genes identified in this study are listed below the traits, and the genes with critical SNPs between VC1973A and V2984 with wild species are indicated in red. *phyA*, *phytochome A*; *PKp1*, *plastidial pyruvate kinase*

Taking advantage of the high‐density genetic map constructed using resequencing data, we identified highly plausible candidate genes for each QTL (Table 2; Supplemental Table S8). Marker intervals for the detected QTLs had an average length of ∼388 kb and contained ∼48.7 genes; the average interval is ∼138 kb containing 19 genes, excluding the largest interval (for *Node11‐1*) of ∼2.4 Mb in length containing 286 genes. The QTLs *Height4‐1*, *Node4‐1*, and *SPM4‐1* were located within the same marker interval, with *phyA* (Vradi04g00002773) as the candidate gene, which was previously identified as the candidate gene underlying *DF3‐1* and *DFF3‐1* QTLs (Hwang et al., 2017). The flower initiation QTL *FI4‐1* contained two genes in the marker interval, including *bonsai* (Vradi04g00002764), which affects inflorescent development (Saze & Kakutani, 2007). *Height5‐1* QTL, with the shortest interval, included only one gene (*kinase*; Vradi05g00000394), whereas *Node11‐1*, with the largest interval, contained *early flowering 3* (*ELF3*) as the candidate gene out of 286 genes. *Ethylene response factor 4* (*ERF4*; Vradi03g00002294) and *plastidial pyruvate kinase* (*PKp1*; Vradi07g00000916) were identified as the candidate genes underlying *Branch3‐1* and *SPM7‐1* QTLs, respectively. The *FI9‐1* QTL comprised six resistance (*R*) genes. Mungbean homologs of two soybean flowering genes, *E3* (*phyA*) and *J* (*ELF3*), were identified as candidate genes for *Height4‐1*, *Node4‐1*, and *SPM4‐1* and *Node11‐1*, respectively (Table 2). We compared nucleotide variations among VC1973A (elite cultivar), V2984 (landrace), and wild species, including *V. reflexopilosa* Hayata var. *glabra*, *V. radiata* var. *sublobata*, *V. hirtella* Ridl., and *V. trinervia* (B.Heyne ex Wight & Arn.) Tateishi & Maxted. Compared with VC1973A, the landrace V2984 and wild mungbean accessions showed several critical nucleotide insertions or deletions in *phyA* (Vradi04g00002773), *kinase* (Vradi05g00000394), and *PKp1* (Vradi07g00000916), resulting in frameshift mutations and the loss of stop codon (Figure 3a,b).

### 3.5 Molecular diversity among Asian mungbean accessions

The new mungbean genome assembly was used as a reference to assess genetic variation among 276 accessions, including 233 cultivars, 42 wild mungbean accessions, and one *V. glabrescens* accession (outgroup), collected from 23 countries (Supplemental Table S9). Raw sequence reads were generated via GBS at a sequencing depth of 4.3–13X, depending on the accession (Supplemental Table S10). The distribution of nucleotide variations was relatively even across the genome (Supplemental Figure S6), with an average nucleotide diversity (π) of 7.63 × 10^−6^ among wild accessions and 2.33 × 10^−6^ among cultivated accessions. Phylogenetic analysis confirmed this reduction of diversity among cultivated mungbean accessions (Supplemental Figure S7). The mungbean cultivar JP231223 from India was the closest to wild accessions, and the cluster closest to wild accessions was dominated by accessions from India and neighboring regions including Pakistan, Myanmar, and China (Supplemental Figure S8). This supports the hypothesis that mungbean was first domesticated in India (Fuller, 2007). Phenotypically, JP231223 has smaller seeds and lower yield than average, but it is not an outlier when compared with other cultivated accessions. This is consistent with the fact that this accession shares more genetic similarity with other cultivated mungbeans than with the nearest wild accession in this study.

**FIGURE 4:**
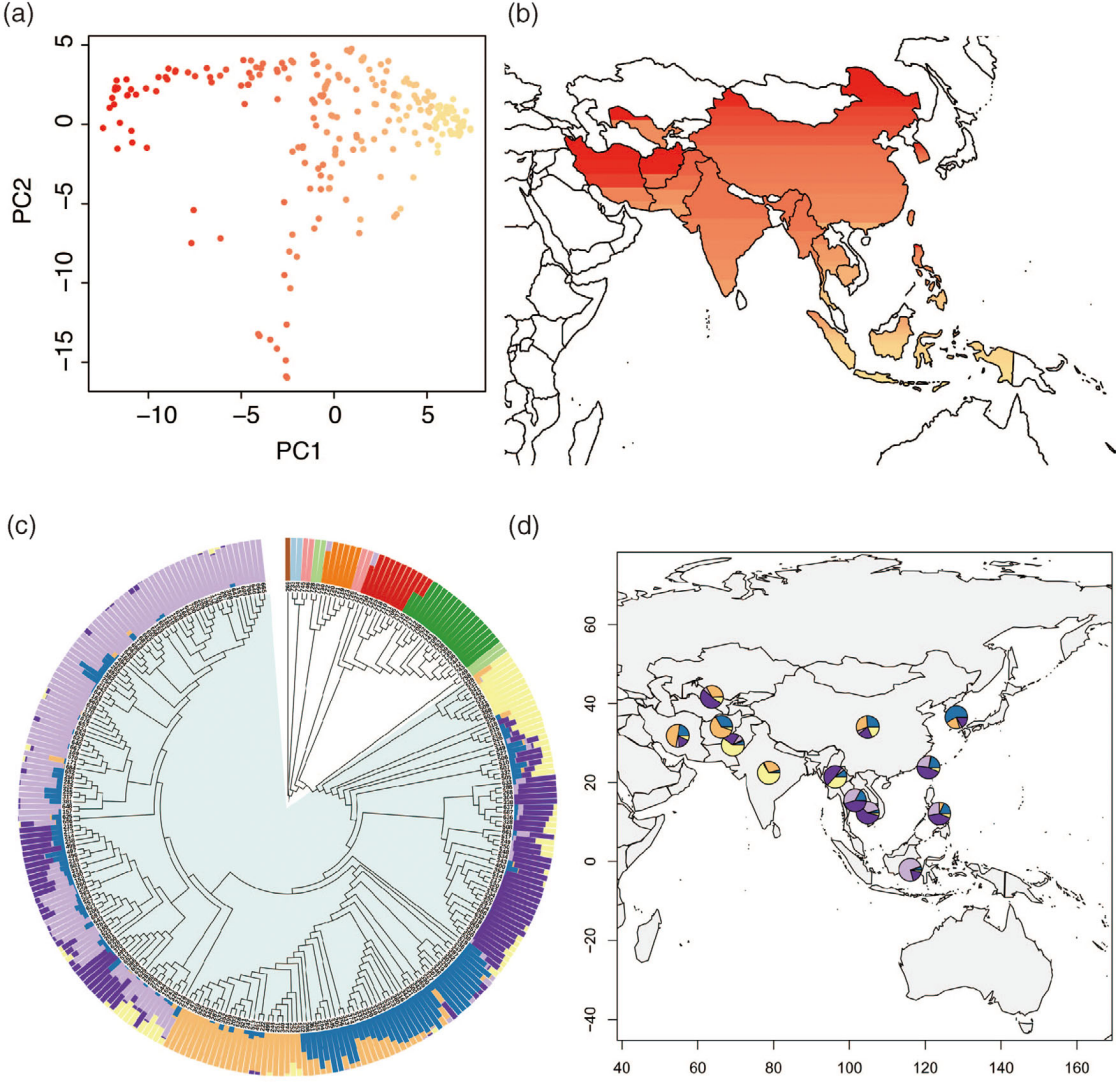
Principle component analysis (PCA) and geographical distribution. (a) PCA using the genotype data, with red‐to‐yellow gradient representing low‐to‐high PC1 values, respectively. (b) Distribution of PC1 values of all accessions obtained from each country, represented by color values sorted from red to yellow. The sorted color shows strong correlation with the latitude of each country of origin. (c) Pattern of genetic admixture when the population is divided into 12 populations viewed in the context of phylogenetic relationships among the accessions. Tree branches shaded in light blue represent cultivated mungbeans. (d) Proportion of DNA ancestry from admixture analysis in (c) when cultivated accessions were grouped according to their country of origin

A PCA of selected mungbean cultivars with reliable origin data showed some correlation between population group membership and geographical origin. The largest principle component (PC) value (PC1) showed a strong correlation with latitude (Pearson's *r* = −.74, *p* < 2.2 × 10^−16^) (Figure 4a), and a color chart representing sorted PC1 values on the country of origin showed a gradient of PC1 from high latitude countries to tropical countries (Figure 4b). Additionally, the population structure of wild accessions was more complex than that of cultivated accessions, as expected (Figure 4c). When cultivated accessions were grouped according to their country of origin, the genetic background of one subgroup was predominant in accessions that originated from nearby areas (Figure 4d).

### 3.6 Selective sweeps in mungbean cultivars

Based on sequence variation among cultivated and wild accessions, we identified chromosome segments under selective pressure during domestication. A total of 36 selective sweep regions, comprising 224 genes, were detected in mungbean (Figure 2; Supplemental Table S11). The number of genes in each selective sweep region varied from 1 to 14. The GO analysis of these genes showed enrichment in developmental processes such as embryonic development, unidimensional cell growth, and reproductive developmental process. Based on the syntenic relationships, we identified soybean genomic regions homologous to mungbean selective sweep regions (Figure 2). Multiple soybean QTLs related to yield, morphology, and biotic and abiotic resistance were identified in syntenic regions (https://www.soybase.org) (Supplemental Table S12), indicating that the associated traits have been positively selected in mungbean during domestication. The selective sweep region *chr7‐1* and mungbean QTL *SPM7‐1* were located close to each other on Vr07 (∼0.3 Mb apart) (Supplemental Table S11; Table 2). In the soybean homologous region of *chr7‐1* and *SPM7‐1*, a soybean QTL of *seed_weight_50‐16* was reported previously (Supplemental Table S12) (Kato et al., 2014). In mungbean, early and even pod maturity were reported to positively affect grain yield, indicating that QTL information on soybean homologous regions can be transferred to mungbean selective sweep regions, thus supporting translational genomic analysis (Supplemental Table S12) (Chen et al., 2008).

## 4 DISCUSSION

In this study, we constructed a near‐complete reference genome sequence of mungbean using long PacBio reads and an ultra‐high‐resolution linkage map constructed using resequencing data (Table 1). Compared with the previous draft assembly of the mungbean genome constructed using Illumina short reads, with possible polymerase chain reaction bias (Ha et al., 2019), and linkage maps based on the GBS strategy with nonuniform sequence coverage (Kang et al., 2014), the quality of the new mungbean reference genome sequence is significantly improved. Because high‐quality reference genome sequences of several economically important legume species have been updated, the accumulated genomic information on related legume species can be used to validate our assembly data without molecular cytogenetic approaches such as fluorescence in situ hybridization (Chamala et al., 2013; Hoang et al., 2020; Yang et al., 2012). Syntenic analysis with *C. arietinum*, *M. truncatula*, and *V. angularis* provided strong evidence that Chr03, Chr04, Chr05, and Chr08 were misassembled in the previous draft assembly, and these sequences have been corrected in the new assembly. The improved mungbean genome assembly will serve as a reference assembly for *Vigna* species and provides high‐quality genomic information for comparative and translational genomics among legume species.

Although determinate vegetative growth habit has been acquired during domestication, SPM has not yet been achieved in mungbean accessions, including in elite cultivars (Li et al., 2018). In soybean, the initiation of flowering marks the end of the juvenile growth period and subsequently affects plant morphology and grain yield (Takeshima et al., 2016). In mungbean, because indeterminate inflorescences are generated from each node on branches and the upper stem, plant morphology critically influences flowering capacity and eventually affects SPM and grain yield (Figure 3c). VC1973A, which is shorter and has fewer numbers of nodes and branches than V2984, is known to have higher SPM than V2984. Therefore, to elucidate the QTLs that influence SPM, all traits that are affected by the length of the juvenile growth phase, such as plant height, flower initiation, node number, branch number, and SPM itself, need to be evaluated.

**FIGURE 5:**
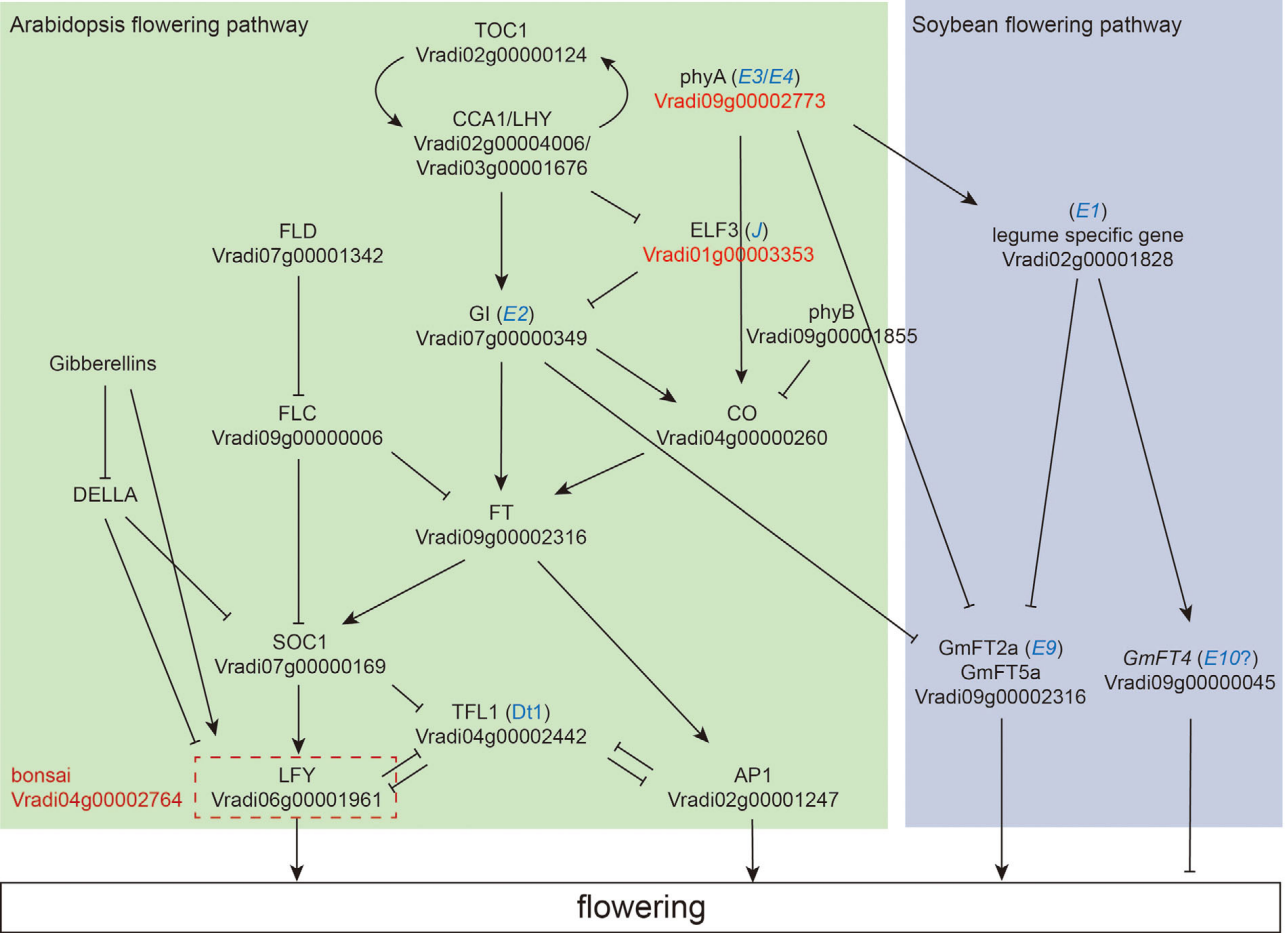
Mungbean homologous genes in flowering pathway. The flowering genes characterized in Arabidopsis are highlighted in the green box. Mungbean homologous genes for each flowering gene are listed, and the genes identified as the candidate genes for quantitative trait loci are indicated in red. Soybean flowering genes are highlighted in the blue box, and flowering locus are indicated in blue in parenthesis. *Bonsai* mutant resembles the phenotype of the *LFY* mutant in Arabidopsis. *AP1*, *apetala1*; *CCA1*, *circadian clock associated 1*; *CO*, *constans*; *FLC*, *flowering locus C*; *FLD*, *flowering locus D*; *FT*, *flowering locus T*; *GI*, *gigantean*; *GmFT*, *glycine max flowering locus*; *LFY*, *leafy*; *LHY*,; *late elongated hypocotyl*; *phyA*, *phytochrome A*; *phyB*, *phytochrome B*; *SOC1*, *suppressor of overexpression of CO1*; *TFL1*, *terminal flower 1*; *TOC1*, *timing of CAB expression 1*

In soybean, which is the best‐characterized model legume related to mungbean, 10 major loci (*E1*–*E9* and *J*) are involved in the regulation of flowering (Takeshima et al., 2016). To date, genes underlying *E1*–*E4*, *E9*, and *J* have been cloned and functionally characterized in soybean (Guo et al., 2015; Lu et al., 2017; Watanabe et al., 2009; Xia et al., 2012; Zhao et al., 2016). In this study, we identified mungbean homologs (Vradi09g00002773 and Vradi11g00000623) of *phyA* (*E3*) and *ELF3* (*J*) as the candidate genes underlying *Height4‐1*, *SPM4‐1* and *Node4‐1*, and *Node11‐1*, respectively (Table 2; Figure 5). The loss‐of‐function allele of *phyA* leads to a short juvenile growth phase and early flowering in soybean under long‐day conditions due to photoperiod insensitivity (Watanabe et al., 2009). Functional characterization shows that *ELF3* delays maturity in soybean, leading to increased plant height, node number, internode length, and grain yield under short‐day conditions (Lu et al., 2017). Thus, *VrELF3* (Vradi11g00000623) was identified as the candidate gene for *Node11‐1* (Table 2).

*VrPhyA* was previously identified as a candidate gene underlying mungbean QTLs *DF3‐1* and *DFF3‐1* (Supplemental Figure S5) (Hwang et al., 2017). In the current study, the QTL interval of *DF3‐1* and *DFF3‐1* was split into two intervals by *Height4‐1*, *Node4‐1* and *SPM4‐1* QTL cluster and *FI4‐1* QTL. *VrPhyA* (Vradi09g00002773) was mapped within the interval containing *Height4‐1*, *Node4‐1*, and *SPM4‐1*, while the interval intersecting the flowering QTL *FI4‐1* contained the *bonsai* gene (Vradi04g00002764), a homolog of AT1G73177, which is responsible for inflorescence development and shoot elongation (Saze & Kakutani, 2007). Although the *bonsai* mutant in petunia (*Pentunia* Juss.) regulates MADS‐box genes, such as *AP1*, and resembles the phenotype of the *leafy* mutant in Arabidopsis, the *bonsai* gene was not investigated separately in the previous flowering QTL study because it was located in the same marker interval as the *VrPhyA* gene (Figure 5) (Hwang et al., 2017; Schorderet et al., 2018). Our results indicate that mungbean homologs of *bonsai* and soybean *E3* and *J* genes regulate juvenile growth traits, such as plant height and node number, as well as flowering initiation, thus affecting the degree of synchronicity in pod maturity (Figure 3).

*Branch3‐1* contained the *AP2/ERF* gene (Vradi03g00002294), a homolog of *AtERF4* (AT3G15210), which negatively regulates ethylene and abscisic acid responses (Liu et al., 2019). Ethylene responsive factors function downstream of the ethylene signaling pathway in plant development and hormone signaling (Müller & Munné‐Bosch, 2015). *Pyruvate kinase* (Vradi07g00000916) was identified at *SMP7‐1*. The Arabidopsis homolog of *PKp1* affects seed filling and embryo development (Baud et al., 2007). Only one gene (Vradi05g00000394) was identified at *Height5‐1*, which is a homolog of *leucine‐rich repeat receptor‐like kinase* (*LRR‐RLK*; AT1G06840). Although the function of *LRR‐RLK* has not been characterized in any model species, it represents a large gene family predominantly involved in developmental processes and hormone perception (Dufayard et al., 2017). Interestingly, all six genes identified at *FI9‐1* were *R* genes. Because a shorter life cycle decreases the possibility of exposure to diseases and pathogens, vegetative lifespan has been associated with defense alleles. For example, expression levels of immunity‐related genes were associated with flowering time in the Arabidopsis natural population (Glander et al., 2017). In soybean, QTLs for disease resistance are reportedly correlated with maturity (Sun et al., 2013), thus supporting the association between *R* genes and flowering time in mungbean.

The candidate genes showed different patterns of nucleotide diversity in landraces and wild accessions of mungbean compared with the reference cultivar (Supplemental Table S13). Landraces and wild mungbean accessions carry frameshift or stop‐loss mutations in *phyA*, *kinase*, and *PKp1* compared with the elite cultivar (Figure 3b), and diverse nucleotide variations were found in the upstream region, 5′ untranslated regions (5′UTR), exons, introns, 3′UTR, and downstream regions of *bonsai*, *ELF3*, and *ERF4* in landraces and wild accessions (Supplemental Table S13). These genetic variations may have been caused by modern breeding. Our results can be used by breeders to improve mungbean elite cultivars with higher synchronicity of pod maturity (Figure 3c).

Using the near‐complete mungbean reference genome, analysis of nucleotide variations in the natural population provided key insights into mungbean domestication, geographical isolation, and genetic admixture. Domestication of mungbean resulted in a dramatic reduction in genetic diversity. The level of nucleotide diversity in cultivated mungbean accessions (π = 2.33 × 10^−6^) was only 30% of that in wild accessions (π = 7.63 × 10^−6^) (Supplemental Figure S6). This reduction in nucleotide diversity due to domestication is worse than that in soybean, where the diversity in landraces and elite cultivars is approximately 47 and 35% of that in wild accessions, respectively (Zhou et al., 2015). Linkage disequilibrium also decayed over a longer distance in cultivated mungbean accessions than in wild accessions (decay cutoff at 71.5 and 2.5 kb, respectively) (Supplemental Figure S9). This study illustrates that wild mungbean germplasm represents a key genetic resource for breeding (Muñoz et al., 2017).

Population structure analysis of mungbean cultivars indicated that geographical isolation occurred following the introduction of different mungbean cultivars in different locations across Asia. Because latitude affects day length and temperature, which have profound effects on growth and flowering, it is likely that this genetic differentiation was driven by selection for good agronomic performance at different latitudes (Figure 4b). This suggests that exchanges of germplasms with different genetic backgrounds among countries will also improve the genetic diversity of mungbean cultivars (Figure 4d).

Accurate genomic information is required for crop improvement. Here, we developed a near‐complete genome sequence of mungbean, which can serve as a reference genome sequence for *Vigna* species. Genomic information of mungbean accessions have revealed that mungbean cultivars still have much potential to be improved. Additional phenotypic data from the 276 accessions will enable future genome‐wide association study. Along with the expression profiles of the candidate genes, cross‐validation of QTL and genome‐wide association study results will help to validate the function of the candidate genes. The genetic and genomic data reported here will serve as excellent resources for genome‐assisted breeding to improve crop quality in Asia.

## AUTHOR CONTRIBUTIONS

Jungmin Ha, Dani Satyawan, and Haneul Jeong contributed equally to this work. Jungmin Ha: Data curation; Visualization; Writing‐original draft; Writing‐review & editing. Dani Satyawan: Data curation; Visualization; Writing‐original draft; Writing‐review & editing. Haneul Jeong: Formal analysis; Software. Eunsoo Lee: Investigation. Kang‐Heum Cho: Software; Validation. Moon Young Kim: Writing‐review & editing. Suk‐Ha Lee: Conceptualization; Funding acquisition; Supervision.

## CONFLICT OF INTEREST

The authors have no conflict of interest to declare.

## Supporting information

Supplementary table 1. Statistics for raw reads generated by Pacbio platform.
Supplementary table 2. Assembly statistic and comparison with the previous assembly.
Supplementary table 3. Statistics for raw reads generated by Illumina Hiseq4000.
Supplementary table 4. Marker details in linkage groups.
Supplementary table 5. Statistics for two genetic maps for scaffold anchoring.
Supplementary table 6. Mapping information of 100 SNP markers per chromosome.
Supplementary table 7. Number of genes from synteny blocks between *V. radiata* and its close species.
Supplementary table 8. List of genes located within the marker intervals associated with QTLs.
Supplementary table 9. Plant materials for genetic and phenotypic variations.
Supplementary table 10. Summary of variants obtained from GBS of 276 wild and cultivated mungbeans.
Supplementary table 11. List of selective sweep regions.
Supplementary table 12. The list of genes in selective sweep regions.
Supplementary table 13. List of nucleotide variations on candidate genes.
Supplementary figure 1. High‐resolution genetic map consisting of 8,966 markers.
Supplementary figure 2. Non‐synchronous maturity in mungbean.
Supplementary Figure 3. Distribution of phenotypes for 187 RIL population.
Supplementary figure 4. LOD scores of identified QTLs for agronomic trait.
Supplementary figure 5. the genomic regions with syntenic relationship between mungbean and soybean genomes.
Supplementary figure 6. Distribution of the number of sequence variants in 400,000 bp intervals on the 11 chromosomes of mungbean.
Supplementary figure 7. Neighbor joining tree for 276 mungbean accessions from 23 countries. Blue circle includes 233 cultivated mungbeans.
Supplementary figure 8. Cladogram of phylogenetic relationship among mungbean accessions in the context of their origins.
Supplementary figure 9. Average distance of LD decay in wild and cultivated mungbeans.

## Data Availability

Genome assembly and annotation data are available at http://plantgenomics.snu.ac.kr/.

## References

[tpg220121-bib-0001] Arumuganathan, K. , & Earle, E. D . (1991). Nuclear DNA content of some important plant species. Plant Molecular Biology Reporter, 9, 208—218. 10.1007/BF02672069

[tpg220121-bib-0002] Baud, S. , Wuillème, S. , Dubreucq, B. , De Almeida, A. , Vuagnat, C. , Lepiniec, L. , Miquel, M. , & Rochat, C . (2007). Function of plastidial pyruvate kinases in seeds of *Arabidopsis thaliana* . Plant Journal, 52, 405–419. 10.1111/j.1365-313X.2007.03232.x 17892448

[tpg220121-bib-0003] Benlloch, R. , Berbel, A. , Ali, L. , Gohari, G. , Millán, T. , & Madueño, F . (2015). Genetic control of inflorescence architecture in legumes. Frontiers in Plant Science, 6, 543. 10.3389/fpls.2015.00543 26257753 PMC4508509

[tpg220121-bib-0004] Boetzer, M. , Henkel, C. V. , Jansen, H. J. , Butler, D. , & Pirovano, W . (2011). Scaffolding pre‐assembled contigs using SSPACE. Bioinformatics, 27, 578–579. 10.1093/bioinformatics/btq683 21149342

[tpg220121-bib-0005] Boetzer, M. , & Pirovano, W . (2012). Toward almost closed genomes with GapFiller. Genome Biology, 13, R56. 10.1186/gb-2012-13-6-r56 22731987 PMC3446322

[tpg220121-bib-0006] Camacho, C. , Coulouris, G. , Avagyan, V. , Ma, N. , Papadopoulos, J. , Bealer, K. , & Madden, T. L . (2009). BLAST+: Architecture and applications. BMC Bioinformatics, 10, 421. 10.1186/1471-2105-10-421 20003500 PMC2803857

[tpg220121-bib-0007] Chamala, S. , Chanderbali, A. S. , Der, J. P. , Lan, T. , Walts, B. , Albert, V. A. , Depamphilis, C. W. , Leebens‐Mack, J. , Rounsley, S. , Schuster, S. C. , Wing, R. A. , Xiao, N. , Moore, R. , Soltis, P. S. , Soltis, D. E. , & Barbazuk, W. B. (2013). Assembly and validation of the genome of the nonmodel basal angiosperm Amborella. Science, 342, 1516–1517. 10.1126/science.1241130 24357320

[tpg220121-bib-0008] Chen, H. , Patterson, N. , & Reich, D . (2010). Population differentiation as a test for selective sweeps. Genome Research, 20, 393–402. 10.1101/gr.100545.109 20086244 PMC2840981

[tpg220121-bib-0009] Chen, L.‐R. , Markhart, A. H. , Shanmugasundaram, S. , & Lin, T.‐Y . (2008). Early developmental and stress responsive ESTs from mungbean, *Vigna radiata* (L.) Wilczek, seedlings. Plant Cell Reports, 27, 535–552. 10.1007/s00299-007-0488-3 18060406

[tpg220121-bib-0010] Chin, C.‐S. , Peluso, P. , Sedlazeck, F. J. , Nattestad, M. , Concepcion, G. T. , Clum, A. , Dunn, C. , O'Malley, R. , Figueroa‐Balderas, R. , Morales‐Cruz, A. , Cramer, G. R. , Delledonne, M. , Luo, C. , Ecker, J. R. , Cantu, D. , Rank, D. R. , & Schatz, M. C . (2016). Phased diploid genome assembly with single molecule real‐time sequencing . bioRxiv. 10.1101/056887 PMC5503144 27749838

[tpg220121-bib-0011] Cingolani, P. , Platts, A. , Wang, L. L. , Coon, M. , Nguyen, T. , Wang, L. , Land, S. J. , Lu, X. , & Ruden, D. M . (2012). A program for annotating and predicting the effects of single nucleotide polymorphisms, SnpEff: SNPs in the genome of *Drosophila melanogaster* strain w1118; iso‐2; iso‐3. Fly (Austin), 6, 80–92. 10.4161/fly.19695 22728672 PMC3679285

[tpg220121-bib-0012] Danecek, P. , Auton, A. , Abecasis, G. , Albers, C. A. , Banks, E. , Depristo, M. A. , Handsaker, R. E. , Lunter, G. , Marth, G. T. , Sherry, S. T. , McVean, G. , & Durbin, R . (2011). The variant call format and VCFtools. Bioinformatics, 27, 2156–2158. 10.1093/bioinformatics/btr330 21653522 PMC3137218

[tpg220121-bib-0013] Dufayard, J.‐F. , Bettembourg, M. , Fischer, I. , Droc, G. , Guiderdoni, E. , Périn, C. , Chantret, N. , & Diévart, A . (2017). New insights on leucine‐rich repeats receptor‐like kinase orthologous relationships in angiosperms. Frontiers in Plant Science, 8. 10.3389/fpls.2017.00381 PMC5380761 28424707

[tpg220121-bib-0014] Elshire, R. J. , Glaubitz, J. C. , Sun, Q. , Poland, J. A. , Kawamoto, K. , Buckler, E. S. , & Mitchell, S. E . (2011). A robust, simple genotyping‐by‐sequencing (GBS) approach for high diversity species. PLOS ONE, 6, e19379. 10.1371/journal.pone.0019379 21573248 PMC3087801

[tpg220121-bib-0015] English, A. C. , Richards, S. , Han, Y. , Wang, M. , Vee, V. , Qu, J. , Qin, X. , Muzny, D. M. , Reid, J. G. , Worley, K. C. , & Gibbs, R. A . (2012). Mind the gap: Upgrading genomes with Pacific Biosciences RS long‐read sequencing technology. PLOS ONE, 7, e47768. 10.1371/journal.pone.0047768 23185243 PMC3504050

[tpg220121-bib-0016] Evanno, G. , Regnaut, S. , & Goudet, J. (2005). Detecting the number of clusters of individuals using the software structure: A simulation study. Molecular Ecology, 14(8), 2611–2620. 10.1111/j.1365-294X.2005.02553.x 15969739

[tpg220121-bib-0017] Fuller, D. Q. (2007). Contrasting patterns in crop domestication and domestication rates: Recent archaeobotanical insights from the Old World. Annals of Botany, 100, 903–924. 10.1093/aob/mcm048 17495986 PMC2759199

[tpg220121-bib-0018] Funatsuki, H. , Kawaguchi, K. , Matsuba, S. , Sato, Y. , & Ishimoto, M . (2005). Mapping of QTL associated with chilling tolerance during reproductive growth in soybean. Theoretical and Applied Genetics, 111, 851–861. 10.1007/s00122-005-0007-2 16059730

[tpg220121-bib-0019] Glander, S. , He, F. , Schmitz, G. , Witten, A. , Telschow, A. , & de Meaux, J . (2017). Assortment of flowering time and defense alleles in natural *Arabidopsis thaliana* populations suggests co‐evolution between defense and vegetative lifespan strategies. bioRxiv. 10.1101/131136 PMC6133262 30215800

[tpg220121-bib-0020] Guo, G. , Xu, K. , Zhang, X. , Zhu, J. , Lu, M. , Chen, F. , Liu, L. , Xi, Z.‐Y. , Bachmair, A. , Chen, Q. , & Fu, Y.‐F . (2015). Extensive analysis of GmFTL and GmCOL expression in northern soybean cultivars in field conditions. PLOS ONE, 10, e0136601. 10.1371/journal.pone.0136601 26371882 PMC4570765

[tpg220121-bib-0021] Guzman, P. S. , Diers, B. W. , Neece, D. J. , St Martin, S. K. , Leroy, A. R. , Grau, C. R. , Hughes, T. J. , & Nelson, R. L . (2007). QTL associated with yield in three backcross‐derived populations of soybean. Crop Science, 47, 111–122. 10.2135/cropsci2006.01.0003

[tpg220121-bib-0022] Ha, J. , Kwon, H. , Cho, K.‐H. , Yoon, M. Y. , Kim, M. Y. , & Lee, S.‐H . (2020a). Identification of epigenetic variation associated with synchronous pod maturity in mungbean (*Vigna radiata* L.). Scientific Reports, 10, 1–8. 10.1038/s41598-020-74520-z 33060755 PMC7562708

[tpg220121-bib-0023] Ha, J. , Shim, S. , Lee, T. , Kang, Y. J. , Hwang, W. J. , Jeong, H. , Laosatit, K. , Lee, J. , Kim, S. K. , Satyawan, D. , Lestari, P. , Yoon, M. Y. , Kim, M. Y. , Chitikineni, A. , Tanya, P. , Somta, P. , Srinives, P. , Varshney, R. K. , & Lee, S.‐H . (2019). Genome sequence of *Jatropha curcas* L., a non‐edible biodiesel plant, provides a resource to improve seed‐related traits. Plant Biotechnology Journal, 17, 517–530. 10.1111/pbi.12995 30059608 PMC6335072

[tpg220121-bib-0024] Ha, J. , Shim, S. , Lee, T. , Lee, E. , Yang, X. , Jeong, H. , Kim, M. Y. , & Lee, S.‐H . (2020b). Transcriptomic and biochemical analyses of the accumulation of sucrose in mungbean (*Vigna radiata* (L.) Wilczek) leaves after pod removal. Theoretical and Applied Genetics, 133(8), 2355–2362. 10.1007/s00122-020-03603-2 32447408

[tpg220121-bib-0025] Hill, W. G. , & Robertson, A . (1968). Linkage disequilibrium in finite populations. Theoretical and Applied Genetics, 38, 226–231. 10.1007/BF01245622 24442307

[tpg220121-bib-0026] Hoang, P. T. N. , Fiebig, A. , Novák, P. , Macas, J. , Cao, H. X. , Stepanenko, A. , Chen, G. , Borisjuk, N. , Scholz, U. , & Schubert, I. (2020). Chromosome‐scale genome assembly for the duckweed *Spirodela intermedia*, integrating cytogenetic maps, PacBio and Oxford Nanopore libraries. Scientific reports, 10, 1–14. 10.1038/s41598-020-75728-9 33154426 PMC7645714

[tpg220121-bib-0027] Hwang, W. J. , Ha, J. , Lee, T. , Jeong, H. , Kim, M. Y. , Kim, S. K. , Lee, Y.‐H.o , Jung, J.i W. , & Lee, S.‐H . (2017). A candidate flowering gene in mungbean is homologous to a soybean *Phytochrome A* gene. Euphytica, 213, 79. 10.1007/s10681-017-1866-8

[tpg220121-bib-0028] Kabelka, E. A. , Diers, B. W. , Fehr, W. R. , Leroy, A. R. , Baianu, I. C. , You, T. , Neece, D. J. , & Nelson, R. L . (2004). Putative alleles for increased yield from soybean plant introductions. Crop Science, 44, 784–791. 10.2135/cropsci2004.7840

[tpg220121-bib-0029] Kang, Y. J. , Kim, S. K. , Kim, M. Y. , Lestari, P. , Kim, K. H. , Ha, B.‐K. , Jun, T. H. , Hwang, W. J. , Lee, T. , Lee, J. , Shim, S. , Yoon, M. Y. , Jang, Y. E. , Han, K. S. , Taeprayoon, P. , Yoon, N. , Somta, P. , Tanya, P. , Kim, K. S. , … Lee, S.‐H . (2014). Genome sequence of mungbean and insights into evolution within *Vigna* species. Nature Communications, 5, 5443. 10.1038/ncomms6443 PMC4241982 25384727

[tpg220121-bib-0030] Kang, Y. J. , Satyawan, D. , Shim, S. , Lee, T. , Lee, J. , Hwang, W. J. , Kim, S. K. , Lestari, P. , Laosatit, K. , Kim, K. H. , Ha, T. J. , Chitikineni, A. , Kim, M. Y. , Ko, J.‐M. , Gwag, J.‐G. , Moon, J.‐K. , Lee, Y.‐H. , Park, B.‐S. , Varshney, R. K. , & Lee, S.‐H. (2015). Draft genome sequence of adzuki bean, *Vigna angularis* . Scientific Reports, 5, srep08069. 10.1038/srep08069 PMC5389050 25626881

[tpg220121-bib-0031] Kato, S. , Sayama, T. , Fujii, K. , Yumoto, S. , Kono, Y. , Hwang, T.‐Y. , Kikuchi, A. , Takada, Y. , Tanaka, Y. , Shiraiwa, T. , & Ishimoto, M . (2014). A major and stable QTL associated with seed weight in soybean across multiple environments and genetic backgrounds. Theoretical and Applied Genetics, 127, 1365–1374. 10.1007/s00122-014-2304-0 24718925

[tpg220121-bib-0032] Khan, N. A. , Githiri, S. M. , Benitez, E. R. , Abe, J. , Kawasaki, S. , Hayashi, T. , & Takahashi, R . (2008). QTL analysis of cleistogamy in soybean. Theoretical and Applied Genetics, 117, 479–487. 10.1007/s00122-008-0792-5 18506418

[tpg220121-bib-0033] Kim, S. K. , Nair, R. M. , Lee, J. , & Lee, S.‐H. (2015). Genomic resources in mungbean for future breeding programs. Frontiers in Plant Science, 6, 626. 10.3389/fpls.2015.00626 26322067 PMC4530597

[tpg220121-bib-0034] Koren, S. , Walenz, B. P. , Berlin, K. , Miller, J. R. , Bergman, N. H. , & Phillippy, A. M . (2017). Canu: Scalable and accurate long‐read assembly via adaptive *k*‐mer weighting and repeat separation. bioRxiv. 10.1101/071282 PMC5411767 28298431

[tpg220121-bib-0035] Krzywinski, M. , Schein, J. , Birol, I. , Connors, J. , Gascoyne, R. , Horsman, D. , Jones, S. J. , & Marra, M. A . (2009). Circos: An information aesthetic for comparative genomics. Genome Research, 19, 1639–1645. 10.1101/gr.092759.109 19541911 PMC2752132

[tpg220121-bib-0036] Lee, S. H. , Bailey, M. A. , Mian, M. A. R. , Shipe, E. R. , Ashley, D. A. , Parrott, W. A. , Hussey, R. S. , & Boerma, H. R . (1996). Identification of quantitative trait loci for plant height, lodging, and maturity in a soybean population segregating for growth habit. Theoretical and Applied Genetics, 92, 516–523. 10.1007/BF00224553 24166318

[tpg220121-bib-0037] Letunic, I. , & Bork, P . (2006). Interactive Tree Of Life (iTOL): An online tool for phylogenetic tree display and annotation. Bioinformatics, 23, 127–128. 10.1093/bioinformatics/btl529 17050570

[tpg220121-bib-0038] Li, H. (2013). Aligning sequence reads, clone sequences and assembly contigs with BWA‐MEM. arXiv. https://arxiv.org/abs/1303.3997

[tpg220121-bib-0039] Li, H. , Handsaker, B. , Wysoker, A. , Fennell, T. , Ruan, J. , Homer, N. , Marth, G. , Abecasis, G. , Durbin, R. , & 1000 Genome Project Data Processing Subgroup . (2009). The sequence alignment/map format and SAMtools. Bioinformatics, 25, 2078–2079. 10.1093/bioinformatics/btp352 19505943 PMC2723002

[tpg220121-bib-0040] Li, S. , Ding, Y. , Zhang, D. , Wang, X. , Tang, X. , Dai, D. , Jin, H. , Lee, S.‐H. , Cai, C. , & Ma, J . (2018). Parallel domestication with a broad mutational spectrum of determinate stem growth habit in leguminous crops. Plant Journal, 96, 761–771. 10.1111/tpj.14066 30112860

[tpg220121-bib-0041] Liu, M. , Sun, W. , Ma, Z. , Zheng, T. , Huang, L.i , Wu, Q. , Zhao, G. , Tang, Z. , Bu, T. , Li, C. , & Chen, H . (2019). Genome‐wide investigation of the AP2/ERF gene family in tartary buckwheat (*Fagopyum Tataricum*). BMC Plant Biology [Electronic Resource], 19, 84. 10.1186/s12870-019-1681-6 30786863 PMC6381666

[tpg220121-bib-0042] Lu, S. , Zhao, X. , Hu, Y. , Liu, S. , Nan, H. , Li, X. , Fang, C. , Cao, D. , Shi, X. , Kong, L. , Su, T. , Zhang, F. , Li, S. , Wang, Z. , Yuan, X. , Cober, E. R. , Weller, J. L. , Liu, B. , Hou, X. , … Kong, F . (2017). Natural variation at the soybean J locus improves adaptation to the tropics and enhances yield. Nature Genetics, 49, 773. 10.1038/ng.3819 28319089

[tpg220121-bib-0043] Maere, S. , Heymans, K. , & Kuiper, M . (2005). BiNGO: A Cytoscape plugin to assess overrepresentation of gene ontology categories in biological networks. Bioinformatics, 21, 3448–3449. 10.1093/bioinformatics/bti551 15972284

[tpg220121-bib-0044] Meng, L. , Li, H. , Zhang, L. , & Wang, J . (2015). QTL IciMapping: Integrated software for genetic linkage map construction and quantitative trait locus mapping in biparental populations. Crop Journal, 3, 269–283. 10.1016/j.cj.2015.01.001

[tpg220121-bib-0045] Müller, M. , & Munné‐Bosch, S . (2015). Ethylene response factors: A key regulatory hub in hormone and stress signaling. Plant Physiology, 169, 32–41. 10.1104/pp.15.00677 26103991 PMC4577411

[tpg220121-bib-0046] Muñoz, N. , Liu, A. , Kan, L. , Li, M.‐W. , & Lam, H.‐M . (2017). Potential uses of wild germplasms of grain legumes for crop improvement. International Journal of Molecular Sciences, 18, 328. 10.3390/ijms18020328 28165413 PMC5343864

[tpg220121-bib-0047] Nair, R. , Schafleitner, R. , Kenyon, L. , Srinivasan, R. , Easdown, W. , Ebert, A. , & Hanson, P . (2012). Genetic improvement of mungbean. SABRAO Journal of Breeding and Genetics, 44, 177–190.

[tpg220121-bib-0048] Parra, G. , Bradnam, K. , & Korf, I . (2007). CEGMA: A pipeline to accurately annotate core genes in eukaryotic genomes. Bioinformatics, 23, 1061–1067. 10.1093/bioinformatics/btm071 17332020

[tpg220121-bib-0049] Peakall, R. , & Smouse, P. E . (2006). GENALEX 6: Genetic analysis in Excel. Population genetic software for teaching and research. Molecular Ecology Notes, 6, 288–295. 10.1111/j.1471-8286.2005.01155.x PMC3463245 22820204

[tpg220121-bib-0050] Perrier, X. , & Jacquemoud‐Collet, J . (2015). *DARwin* [Software]. https://darwin.cirad.fr/

[tpg220121-bib-0051] Pritchard, J. K. , Stephens, M. , & Donnelly, P . (2000). Inference of population structure using multilocus genotype data. Genetics, 155, 945–959. 10.1093/genetics/155.2.945 10835412 PMC1461096

[tpg220121-bib-0052] Saze, H. , & Kakutani, T . (2007). Heritable epigenetic mutation of a transposon‐flanked *Arabidopsis* gene due to lack of the chromatin‐remodeling factor DDM1. EMBO Journal, 26, 3641–3652. 10.1038/sj.emboj.7601788 17627280 PMC1949009

[tpg220121-bib-0053] Schafleitner, R. , Huang, S. , Chu, S. , Yen, J. , Lin, C. , Yan, M. , Krishnan, B. , Liu, M. , Lo, H. , Chen, C. , Chen, L. O. , Wu, D. , Bui, T.‐G. T. , Ramasamy, S. , Tung, C. , & Nair, R . (2016). Identification of single nucleotide polymorphism markers associated with resistance to bruchids (*Callosobruchus* spp.) in wild mungbean (*Vigna radiata* var. *sublobata*) and cultivated *V. radiata* through genotyping by sequencing and quantitative trait locus analysis. BMC Plant Biology, 16, 159. 10.1186/s12870-016-0847-8 27422285 PMC4946214

[tpg220121-bib-0054] Schmutz, J. , Cannon, S. B. , Schlueter, J. , Ma, J. , Mitros, T. , Nelson, W. , Hyten, D. L. , Song, Q. , Thelen, J. J. , Cheng, J. , Xu, D. , Hellsten, U. , May, G. D. , Yu, Y. , Sakurai, T. , Umezawa, T. , Bhattacharyya, M. K. , Sandhu, D. , Valliyodan, B. , … Jackson, S. A . (2010). Genome sequence of the palaeopolyploid soybean. Nature, 463, 178–183. 10.1038/nature08670 20075913

[tpg220121-bib-0055] Schorderet, M. , Duvvuru Muni, R. , Fiebig, A. , & Reinhardt, D . (2018). Deregulation of MADS‐box transcription factor genes in a mutant defective in the WUSCHEL‐LIKE HOMEOBOX gene EVERGREEN of Petunia hybrida. Plant Signaling & Behavior, 13, e1471299. 29995575 10.1080/15592324.2018.1471299 PMC6207418

[tpg220121-bib-0056] Shohag, M. J. I. , Wei, Y. , & Yang, X . (2012). Changes of folate and other potential health‐promoting phytochemicals in legume seeds as affected by germination. Journal of Agricultural and Food Chemistry, 60, 9137–9143. 10.1021/jf302403t 22906127

[tpg220121-bib-0057] Simão, F. A. , Waterhouse, R. M. , Ioannidis, P. , Kriventseva, E. V. , & Zdobnov, E. M . (2015). BUSCO: Assessing genome assembly and annotation completeness with single‐copy orthologs. Bioinformatics, 31, 3210–3212. 10.1093/bioinformatics/btv351 26059717

[tpg220121-bib-0058] Sun, D. , Li, W. , Zhang, Z. , Chen, Q. , Ning, H. , Qiu, L. , & Sun, G . (2006). Quantitative trait loci analysis for the developmental behavior of soybean (*Glycine max* L. Merr.). Theoretical and Applied Genetics, 112, 665–673. 10.1007/s00122-005-0169-y 16365761

[tpg220121-bib-0059] Sun, S. , Kim, M. Y. , Van, K. , Lee, Y.‐W. , Li, B. , & Lee, S.‐H . (2013). QTLs for resistance to Phomopsis seed decay are associated with days to maturity in soybean *(Glycine max)* . Theoretical and Applied Genetics, 126, 2029–2038. 10.1007/s00122-013-2115-8 23702513

[tpg220121-bib-0060] Takeshima, R. , Hayashi, T. , Zhu, J. , Zhao, C. , Xu, M. , Yamaguchi, N. , Sayama, T. , Ishimoto, M. , Kong, L. , Shi, X. , Liu, B. , Tian, Z. , Yamada, T. , Kong, F. , & Abe, J . (2016). A soybean quantitative trait locus that promotes flowering under long days is identified as *FT5a*, a *FLOWERING LOCUS T* ortholog. Journal of Experimental Botany, 67, 5247–5258. 10.1093/jxb/erw283 27422993 PMC5014162

[tpg220121-bib-0061] Tang, H. , Krishnakumar, V. , Bidwell, S. , Rosen, B. , Chan, A. , Zhou, S. , Gentzbittel, L. , Childs, K. L. , Yandell, M. , Gundlach, H. , Mayer, K. F. , Schwartz, D. C. , & Town, C. D . (2014). An improved genome release (version Mt4.0) for the model legume Medicago truncatula. BMC Genomics, 15, 312. 10.1186/1471-2164-15-312 24767513 PMC4234490

[tpg220121-bib-0062] Tang, H. , Zhang, X. , Miao, C. , Zhang, J. , Ming, R. , Schnable, J. C. , Schnable, P. S. , Lyons, E. , & Lu, J . (2015). ALLMAPS: Robust scaffold ordering based on multiple maps. Genome Biology, 16, 3. 10.1186/s13059-014-0573-1 25583564 PMC4305236

[tpg220121-bib-0063] Tian, Z. , Wang, X. , Lee, R. , Li, Y. , Specht, J. E. , Nelson, R. L. , McClean, P. E. , Qiu, L. , & Ma, J . (2010). Artificial selection for determinate growth habit in soybean. Proceedings of the National Academy of Sciences of the United States of America, 107, 8563–8568. 10.1073/pnas.1000088107 20421496 PMC2889302

[tpg220121-bib-0064] Van Ooijen, J. (2006). *JoinMap 4.1* [Software]. Kyazma.

[tpg220121-bib-0065] Varshney, R. K. , Song, C. , Saxena, R. K. , Azam, S. , Yu, S. , Sharpe, A. G. , Cannon, S. , Baek, J. , Rosen, B. D. , Tar'an, B. , Millan, T. , Zhang, X. , Ramsay, L. D. , Iwata, A. , Wang, Y. , Nelson, W. , Farmer, A. D. , Gaur, P. M. , Soderlund, C. , … Cook, D. R . (2013). Draft genome sequence of chickpea (*Cicer arietinum*) provides a resource for trait improvement. Nature Biotechnology, 31, 240–246. 10.1038/nbt.2491 23354103

[tpg220121-bib-0066] Wang, Y. , Tang, H. , Debarry, J. D. , Tan, X. , Li, J. , Wang, X. , Lee, T.‐H. , Jin, H. , Marler, B. , Guo, H. , Kissinger, J. C. , & Paterson, A. H . (2012). MCScanX: A toolkit for detection and evolutionary analysis of gene synteny and collinearity. Nucleic Acids Research, 40, e49–e49. 10.1093/nar/gkr1293 22217600 PMC3326336

[tpg220121-bib-0067] Watanabe, S. , Hideshima, R. , Xia, Z. , Tsubokura, Y. , Sato, S. , Nakamoto, Y. , Yamanaka, N. , Takahashi, R. , Ishimoto, M. , Anai, T. , Tabata, S. , & Harada, K . (2009). Map‐based cloning of the gene associated with the soybean maturity locus E3. Genetics, 182, 1251–1262. 10.1534/genetics.108.098772 19474204 PMC2728863

[tpg220121-bib-0068] Xia, Z. , Watanabe, S. , Yamada, T. , Tsubokura, Y. , Nakashima, H. , Zhai, H. , Anai, T. , Sato, S. , Yamazaki, T. , Lu, S. , Wu, H. , Tabata, S. , & Harada, K . (2012). Positional cloning and characterization reveal the molecular basis for soybean maturity locus E1 that regulates photoperiodic flowering. Proceedings of the National Academy of Sciences of the United States of America, 109, E2155–E2164. 10.1073/pnas.1117982109 22619331 PMC3420212

[tpg220121-bib-0069] Yang, L. , Koo, D.‐H. , Li, Y. , Zhang, X. , Luan, F. , Havey, M. J. , Jiang, J. , & Weng, Y. (2012). Chromosome rearrangements during domestication of cucumber as revealed by high‐density genetic mapping and draft genome assembly. The Plant Journal, 71, 895–906. 10.1111/j.1365-313X.2012.05017.x 22487099

[tpg220121-bib-0070] Zhao, C. , Takeshima, R. , Zhu, J. , Xu, M. , Sato, M. , Watanabe, S. , Kanazawa, A. , Liu, B. , Kong, F. , Yamada, T. , & Abe, J . (2016). A recessive allele for delayed flowering at the soybean maturity locus *E9* is a leaky allele of *FT2a*, a *FLOWERING LOCUS T* ortholog. BMC Plant Biology, 16, 20. 10.1186/s12870-016-0704-9 26786479 PMC4719747

[tpg220121-bib-0071] Zhou, Z. , Jiang, Y. , Wang, Z. , Gou, Z. , Lyu, J. , Li, W. , Yu, Y. , Shu, L. , Zhao, Y. , Ma, Y. , Fang, C. , Shen, Y. , Liu, T. , Li, C. , Li, Q. , Wu, M. , Wang, M. , Wu, Y. , Dong, Y. , … Tian, Z . (2015). Resequencing 302 wild and cultivated accessions identifies genes related to domestication and improvement in soybean. Nature Biotechnology, 33, 408–414. 10.1038/nbt.3096 25643055

