# Supplementary material for: A near‐complete genome sequence of mungbean (Vigna radiata L.) provides key insights into the modern breeding program
Source: Plant Genome. 2021 Jul 18;14(3):e20121. doi: 10.1002/tpg2.20121 (PMC12807378; doi:10.1002/tpg2.20121)
Supplement: Supplementary file 1 [file TPG2-14-e20121-s001.pdf]

SUPPLEMENTAL MATERIAL

Supplementary table 1. Statistics for raw reads generated by Pacbio platform.

Total reads	Total bases (bp)	Average read length (bp)	GC (%)	Sequencing depth (X)	NCBI SRA*
6,944,099	37,037,906,144	5333.72	34.78	68.21	SRR9994113

NCBI SRA*: National Center for Biotechnology Information Short Read Archives

For Review Only

Supplementary table 2. Assembly statistic and comparison with the previous assembly.

Genome assembly		Kang et al. (2014)	Improved assembly
minimum contig size		1,000	1,005
Total number	contigs	25,922	1,511
	scaffolds	2,748	487
	superscaffolds	2,469	388
N50 length (kbp)	contigs	42	2,809
	scaffolds	1,516	5,182
	superscaffolds	25,361	47,082
Total length (kbp)	contigs	430,875	473,375
	scaffolds	463,143	475,421
	superscaffolds	464,766	475,744
Ratio of Ns in scaffolds (%)		7.2	0.4
GC contents (%)		33.30	33.27
Number of high-confident genes		22,427	30,958
248 core eukaryotic genes (%)		97	98

For Review Only

Supplementary table 3. Statistics for raw reads generated by Illumina Hiseq4000.
In total, 187 RIL population was re-sequenced. Re-sequencing data were deposited in NCBI SRA database(SRR10083737-SRR10083923).

No. of samples	number of reads per sample on average	Read length (bp)	total bases per sample on average (bp)	Average sequencing depth
187	43,700,528	151	6,598,779,804	12.15x

For Review Only

Supplementary table 4. Marker details in linkage groups.

Linkage group	Total number of mapped markers	Length (cM)	Average interval (cM)
LG1	2,519	2,056.13	0.82
LG2	1,354	1,205.81	0.89
LG3	850	611.70	0.72
LG4	809	739.74	0.92
LG5	714	601.00	0.84
LG6	616	487.24	0.79
LG7	471	427.67	0.91
LG8	250	242.21	0.97
LG9	638	511.15	0.80
LG10	366	305.68	0.84
LG11	379	330.75	0.87
Total	8,966	7519.08	0.85

For Review Only

Supplementary table 5. Statistics for two genetic maps for scaffold anchoring.

	Map1	Map2	Consensus map for scaffold anchoring	
			Anchored	Oriented
Linkage Groups	11	11		
Markers (unique)	8,966	1,475	10,270	10,155
Markers per Mb	21.2	4.1	24.1	26.8
Scaffolds	112	76	110	84
Scaffolds with 1 marker	18	11	17	0
Scaffolds with 2 markers	3	3	3	2
Scaffolds with 3 markers	5	2	4	2
Scaffolds with >=4 markers	86	60	86	80
Total bases	422,950,601 (89.0%)	359,448,445 (75.6%)	426,870,856 (89.8%)	379,617,302 (79.8%)

For Review Only

Supplementary figure 1. High-resolution genetic map consisting of 8,966 markers.
Linkage fragments under each linkage group are all connected from left to right.

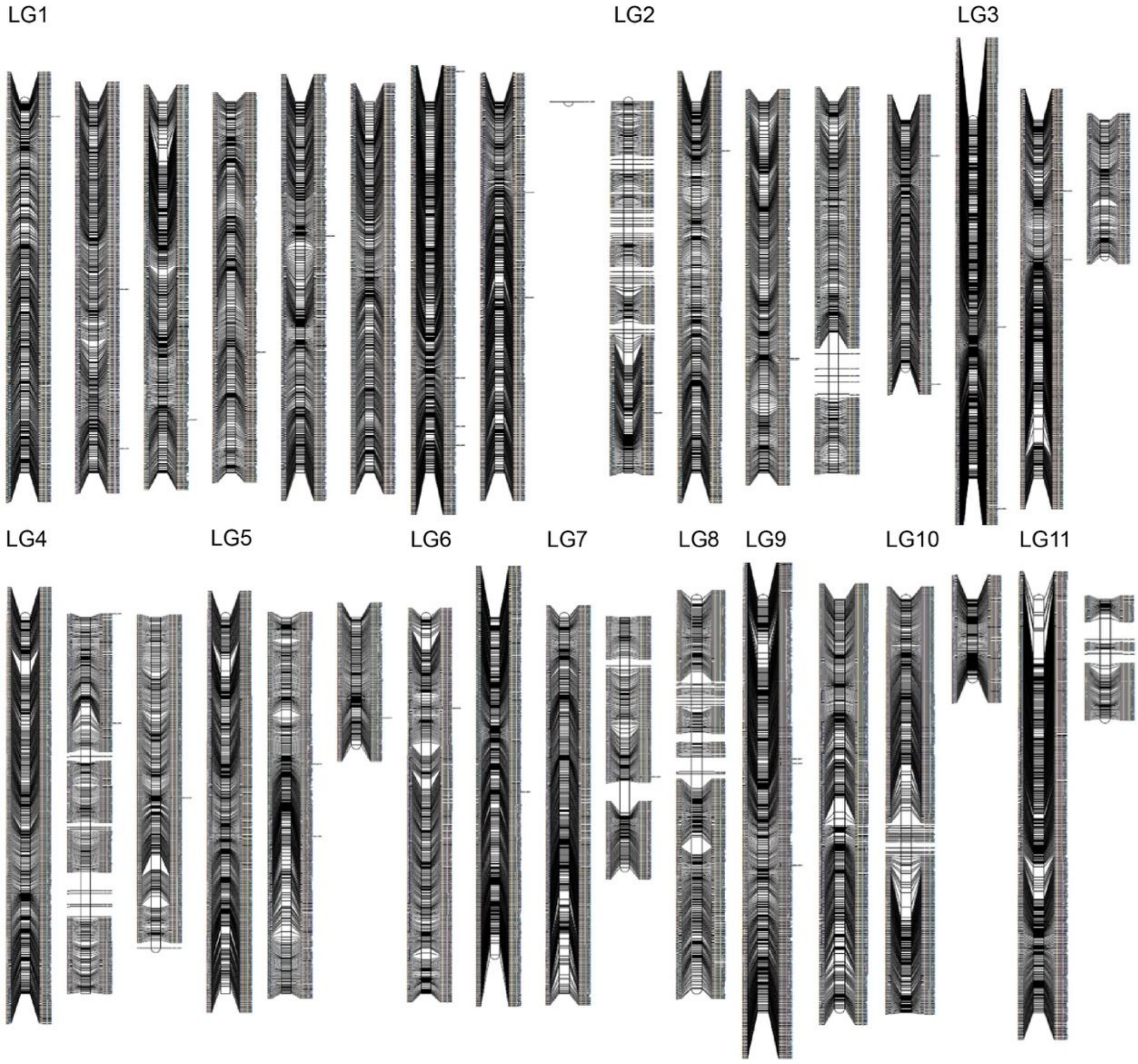

Supplementary table 6. Mapping information of 100 SNP markers per chromosome.

Chromosome of previous reference genome	Mapped chromosome of new reference genome	Number of mapped markers	Percentage of mapped markers (%)
Chr01	Vr1	83	91.2
	Vr2	4	4.4
	Vr9	2	2.2
	Vr4	1	1.1
	Vr11	1	1.1
Chr02	Vr6	68	76.4
	Vr4	18	20.2
	Vr3	3	3.4
Chr03	**Vr4**	91	**100.0**
Chr04	**Vr4**	79	**90.8**
	Vr9	5	5.8
	Vr3	2	2.3
	Vr10	1	1.2
Chr05	**Vr11**	47	**55.3**
	**Vr10**	30	**35.3**
	Vr2	8	9.4
Chr06	Vr5	48	55.2
	Vr6	13	14.9
	Vr2	10	11.5
	Vr9	9	10.3
	Vr1	5	5.8
	Vr3	1	1.2
	Vr4	1	1.2
Chr07	Vr2	62	69.7
	Vr4	8	9.0
	Vr1	5	5.6
	Vr9	5	5.6
	Vr7	3	3.4
	Vr8	3	3.4
	Vr3	2	2.3
	Vr6	1	1.1
Chr08	**Vr4**	41	**47.1**
	Vr7	36	41.4
	Vr3	5	5.8
	Vr5	3	3.5
	Vr1	1	1.2

For Review Only

	Vr8	1	1.2
Chr09	Vr3	82	93.2
	Vr2	5	5.7
	Vr7	1	1.1
Chr10	Vr8	82	90.1
	Vr9	4	4.4
	Vr1	3	3.3
	Vr2	1	1.1
	Vr11	1	1.1
Chr11	Vr9	78	90.7
	Vr1	7	8.1
	Vr4	1	1.2

For Review Only

Supplementary table 7. Number of genes from synteny blocks between *V. radiata* and its close species.

V. radiata	*C. arietinum*		*M. truncatula*		*C. arietinum*	
Chr	Chr	Number of genes (% ratio)	Chr	Number of genes (% ratio)	Chr	Number of genes (% ratio)
Chr03	Ca3	550 (58.1)	Mt7	539 (58.0)	Va4	277 (43.4)
	Ca4	221 (23.4)	Mt1	249 (26.8)	Va7	117 (18.3)
	Ca6	103 (10.9)	Mt3	80 (8.6)	Va9	100 (15.7)
	Ca2	28 (3.0)	Mt5	42 (4.5)	Va6	99 (15.5)
	Ca1	23 (2.4)	Mt2	20 (2.2)	Va1	23 (3.6)
	Ca7	21 (2.2)			Va11	22 (3.5)
Chr04	Ca3	347 (55.4)	Mt7	424 (60.5)	Va4	457 (95.0)
	Ca1	158 (25.2)	Mt2	162 (23.1)	Va9	24 (5.0)
	Ca6	84 (13.4)	Mt4	68 (9.7)		
	Ca2	37 (5.9)	Mt6	27 (3.9)		
			Mt8	20 (2.9)		
Chr08	Ca4	1193 (85.5)	Mt1	1229 (88.3)	Va6	1613 (92.3)
	Ca3	202 (14.5)	Mt7	163 (11.7)	Va4	58 (3.3)
					Va3	54 (3.1)
					Va7	23 (1.3)
Chr05	Ca5	736 (54.2)	Mt3	769 (51.9)	Va5	626 (55.5)
	Ca1	317 (23.4)	Mt2	447 (30.2)	Va9	329 (29.1)
	Ca4	143 (10.5)	Mt4	135 (9.1)	Va11	92 (8.2)
	Ca7	112 (8.3)	Mt1	130 (8.8)	Va1	82 (7.3)
	Ca6	49 (3.6)				

For Review Only

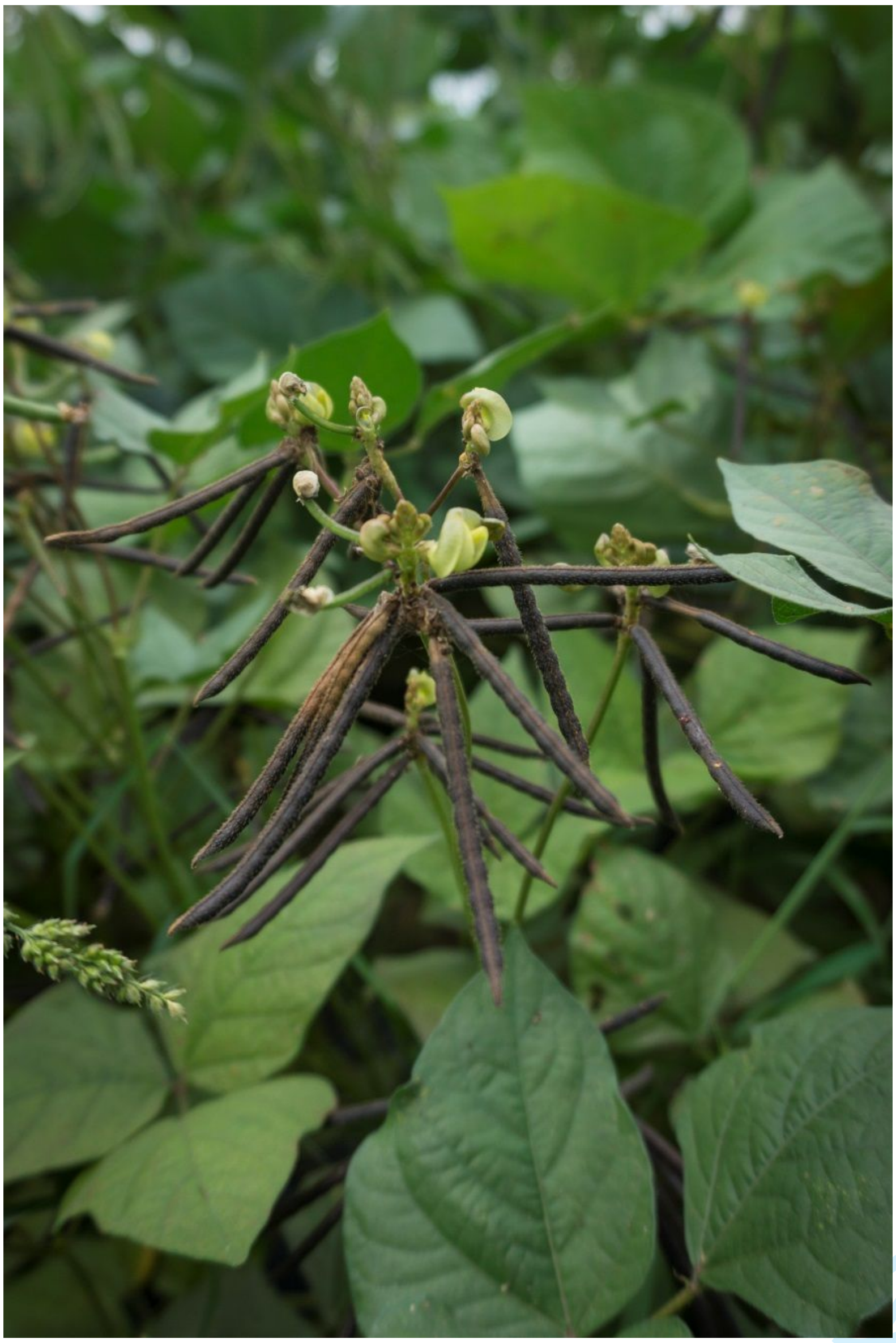

Supplementary figure 2. Non-synchronous maturity in mungbean.

Prolonged productivity of peduncles is one of the reasons of non-synchronous pod maturity in mungbean. New flowers are still formed even when earlier pods from the same peduncles have matured already

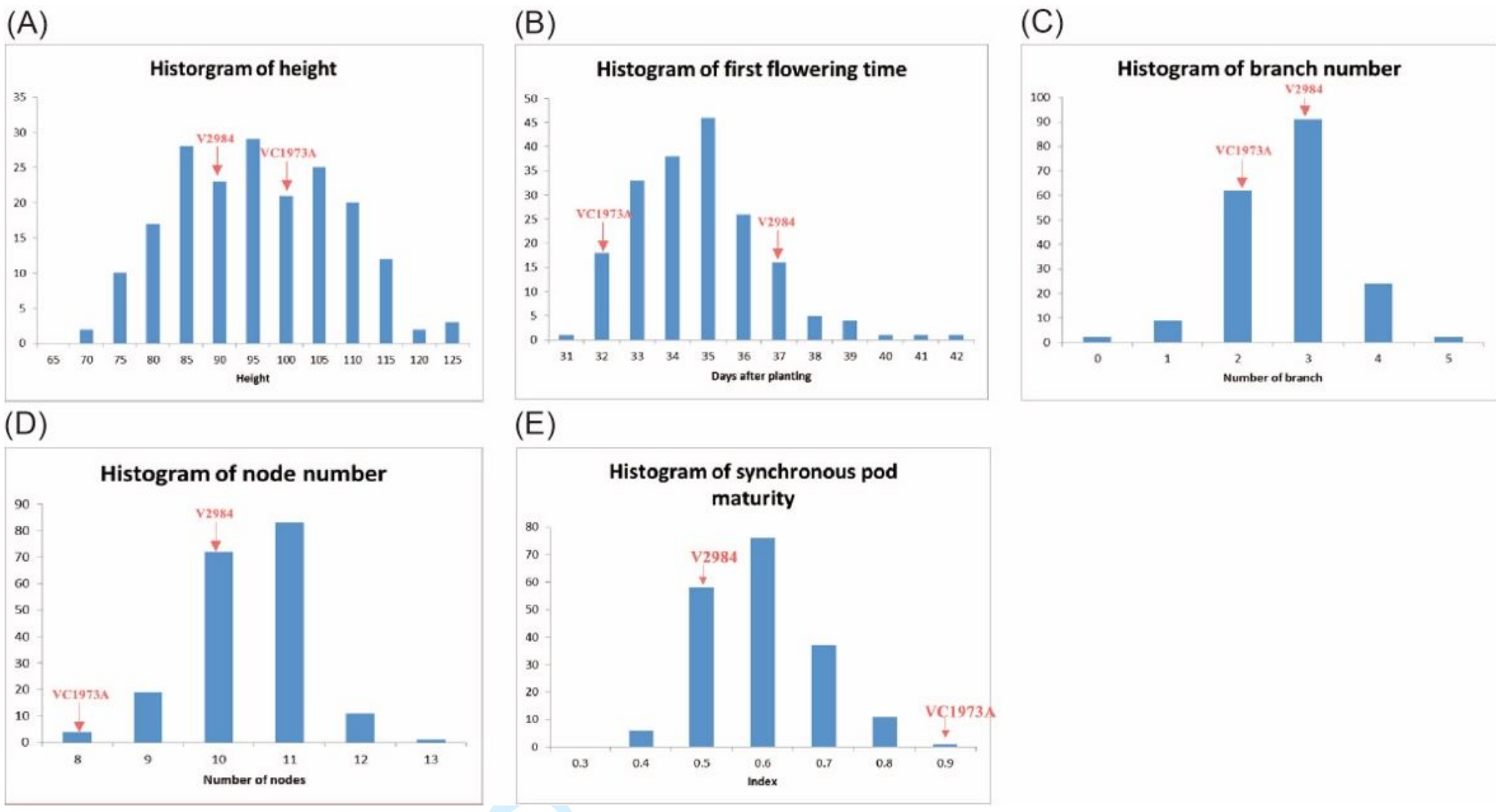

Supplementary Figure 3. Distribution of phenotypes for 187 RIL population.
Phenotypic variations were measured for (A) plant height, (B) first flowering time, (C) branch number, (D) node number and (E) synchronous pod maturity.

For Review Only

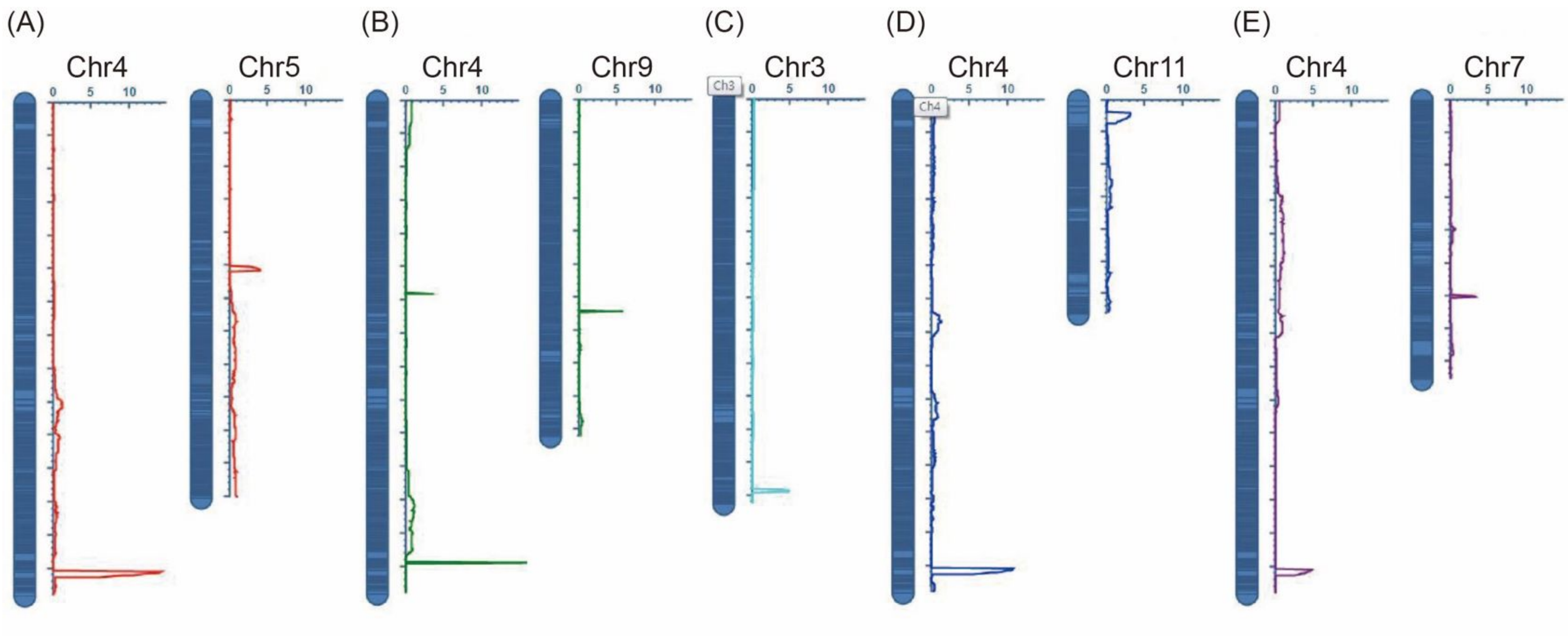

Supplementary figure 4. LOD scores of identified QTLs for agronomic trait.
(A) plant height, (B) first flowering time, (C) number of branch, (D) number of node and (E) synchronous pod maturity

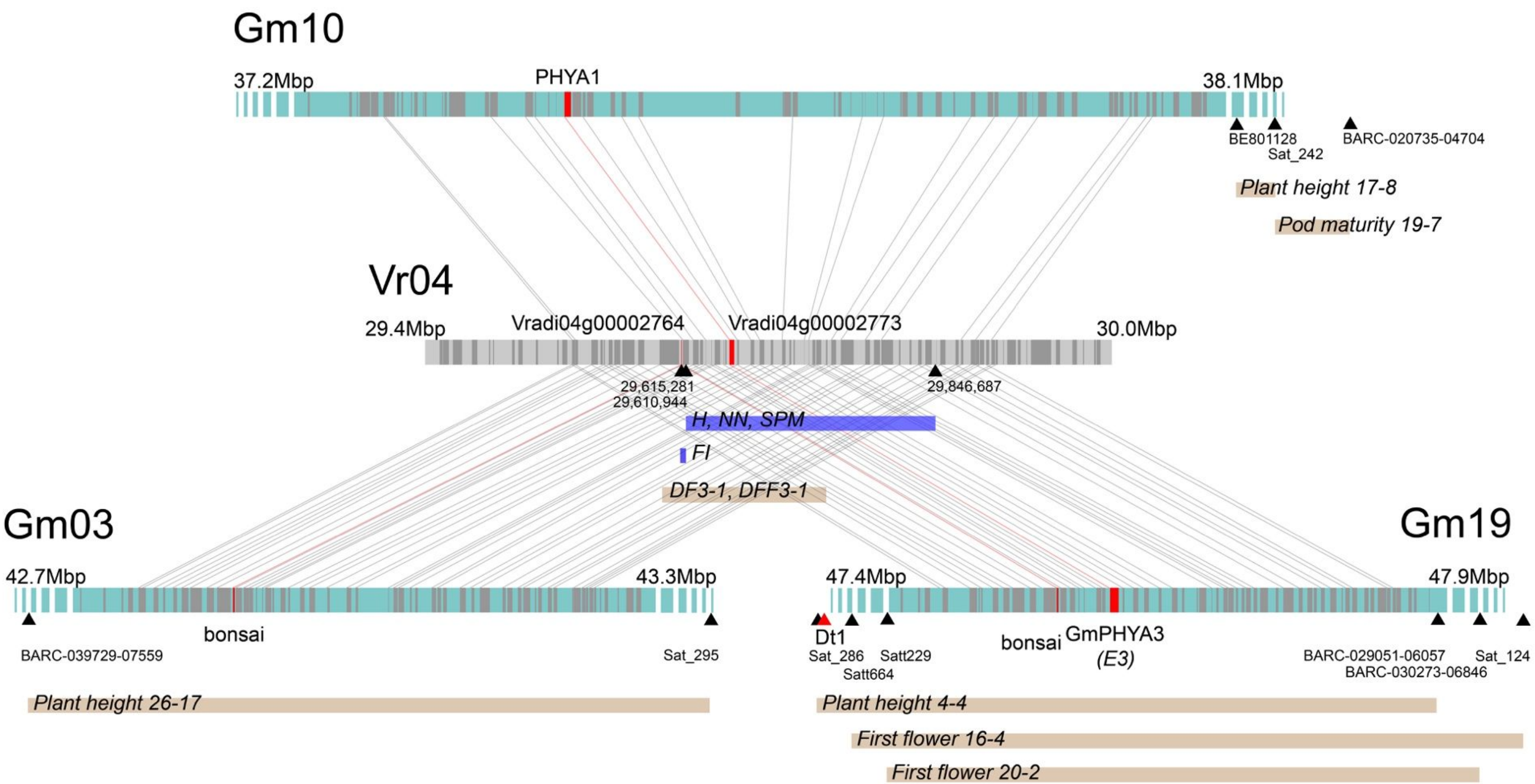

Supplementary figure 5. the genomic regions with syntenic relationship between mungbean and soybean genomes. Mungbean chromosome 4 is indicated by Vr04, and soybean chromosome 3, 10 and 19 are indicated by Gm03, Gm10 and Gm19. Previously identified soybean QTLs are indicated by brown rectangles below soybean chromosomes. Purple and brown rectangles below Vr04 represent mungbean QTLs identified in current and previous studies, respectively (H: plant height, NN: number of nodes, SPM: synchronous pod maturity, FI: flowering initiation, DF: days to flowering, DFF: days to first flowering). PHYA indicates phytochroma A. Black arrow heads represent genetic markers associated with the QTLs. Red arrow head indicates *GmDt1*.

Supplementary table 8. List of genes located within the marker intervals associated with QTLs. Candidate genes were indicated in red.

Locus	Gene name	*At* orthologs	Description
Branch3-1	Vradi03g00002265	AT5G20230.1	plastocyanin-like domain containing protein, putative, expressed
	Vradi03g00002266	AT3G15810.1	DUF567 domain containing protein, putative, expressed
	Vradi03g00002267	AT1G80100.1	histidine-containing phosphotransfer protein, putative, expressed
	Vradi03g00002268	AT3G15850.1	fatty acid desaturase 5
	Vradi03g00002269	AT5G20420.1	expressed protein
	Vradi03g00002270	AT3G15840.1	chloroplast post-illumination chlorophyll fluorescence increase protein, putative, expressed
	Vradi03g00002271	AT3G15820.1	expressed protein
	Vradi03g00002272	AT3G15890.1	BRASSINOSTEROID INSENSITIVE 1-associated receptor kinase 1 precursor, putative, expressed
	Vradi03g00002273	AT3G15890.1	BRASSINOSTEROID INSENSITIVE 1-associated receptor kinase 1 precursor, putative, expressed
	Vradi03g00002274	AT5G32440.1	expressed protein
	Vradi03g00002275	AT5G03500.3	mediator of RNA polymerase II transcription subunit 7, putative, expressed
	Vradi03g00002276	AT1G48900.1	signal recognition particle 54 kDa protein, putative, expressed
	Vradi03g00002277	AT3G15930.1	pentatricopeptide, putative, expressed
	Vradi03g00002278	AT1G52510.2	hydrolase, alpha/beta fold family domain containing protein, expressed
	Vradi03g00002279	AT1G15290.1	tetratricopeptide repeat domain containing protein, putative, expressed
	Vradi03g00002280	AT1G15290.1	tetratricopeptide repeat domain containing protein, putative, expressed
	Vradi03g00002281	AT1G80450.1	VQ domain containing protein, putative, expressed
	Vradi03g00002282	AT1G80460.1	FGGY family of carbohydrate kinases, putative, expressed
	Vradi03g00002283	AT5G57300.3	methyltransferase domain containing protein, putative, expressed
	Vradi03g00002284	AT1G15740.1	regulatory subunit, putative, expressed
	Vradi03g00002285	AT1G80490.2	lissencephaly type-1-like homology motif, putative, expressed
	Vradi03g00002286	AT1G15760.1	jp18, putative, expressed
	Vradi03g00002287	AT1G15780.1	expressed protein
	Vradi03g00002288	AT1G15780.1	expressed protein
	Vradi03g00002289	AT2G12400.1	expressed protein
	Vradi03g00002290	AT3G02645.1	plant protein of unknown function domain containing protein, expressed
	Vradi03g00002291	AT4G37850.1	helix-loop-helix DNA-binding domain containing protein, expressed
	Vradi03g00002292	AT4G37850.1	helix-loop-helix DNA-binding domain containing protein, expressed
	Vradi03g00002293	AT4G37850.1	helix-loop-helix DNA-binding domain containing protein, expressed
	Vradi03g00002294	AT3G15210.1	AP2 domain containing protein, expressed
	Vradi03g00002295	AT1G80620.1	ribosomal protein S15 containing protein, expressed
	Vradi03g00002296	AT2G33590.1	reductase, putative, expressed
	Vradi03g00002297	AT1G80630.1	F-box/LRR-repeat protein 3, putative, expressed
	Vradi03g00002298	AT1G80630.1	F-box/LRR-repeat protein 3, putative, expressed
	Vradi03g00002299	AT1G80660.1	plasma membrane ATPase, putative, expressed
	Vradi03g00002300	AT1G15860.2	DCN1, putative, expressed

For Review Only

	Vradi03g00002301	AT1G80690.1	ethylene-responsive element-binding protein, putative, expressed
	Vradi03g00002302	AT1G80700.1	expressed protein
	Vradi03g00002303	AT5G10970.1	ZOS3-07 - C2H2 zinc finger protein, expressed
	Vradi03g00002304	AT1G15880.1	vesicle transport v-SNARE protein, putative, expressed
	Vradi03g00002305	AT1G80760.1	aquaporin protein, putative, expressed
	Vradi03g00002306	AT1G15910.1	transcription factor X1, putative, expressed
	Vradi03g00002307	AT2G32070.1	CAF1 family ribonuclease containing protein, putative, expressed
	Vradi03g00002308	AT1G80810.2	AT hook motif family protein, expressed
FFT4-1	Vradi04g00002764	AT1G73177.1	BONSAI
	Vradi04g00002765	AT5G26742.1	AtRH3, EMB1138
Height4-1	Vradi04g00002765	AT5G26742.1	AtRH3, EMB1138
Node4-1	Vradi04g00002766	AT3G05940.1	domain of unknown function domain containing protein, expressed
SPM4-1	Vradi04g00002767	AT3G14690.1	cytochrome P450 72A1, putative, expressed
	Vradi04g00002768	AT5G26731.1	hypothetical protein
	Vradi04g00002769	AT3G05936.1	hypothetical protein
	Vradi04g00002770	AT4G11160.1	expressed protein
	Vradi04g00002771	AT5G26720.1	ubiquitin carboxyl-terminal hydrolase-like protein
	Vradi04g00002772	AT5G49200.1	nucleic acid binding protein, putative, expressed
	Vradi04g00002773	AT1G09570.1	phytochrome A, putative, expressed
	Vradi04g00002774	AT3G62020.1	Cupin domain containing protein, expressed
	Vradi04g00002775	AT1G69850.1	peptide transporter PTR2, putative, expressed
	Vradi04g00002776	AT3G05910.1	pectinacetylesterase domain containing protein, expressed
	Vradi04g00002777	AT1G09540.1	MYB family transcription factor, putative, expressed
	Vradi04g00002778	AT1G09530.2	helix-loop-helix DNA-binding domain containing protein, expressed
	Vradi04g00002779	AT2G22070.1	pentatricopeptide, putative, expressed
	Vradi04g00002780	AT3G27670.1	RST1, putative, expressed
	Vradi04g00002781	AT5G07040.1	OsFBO23 - F-box and other domain containing protein, expressed
	Vradi04g00002782	AT3G05858.1	expressed protein
	Vradi04g00002783	AT5G26610.3	G-patch domain containing protein, expressed
	Vradi04g00002784	AT1G11910.1	aspartic proteinase oryzasin-1 precursor, putative, expressed
	Vradi04g00002785	AT5G26600.2	aminotransferase, putative, expressed
	Vradi04g00002786	AT3G62200.1	expressed protein
	Vradi04g00002787	AT1G09450.1	serine/threonine-protein kinase Haspin, putative, expressed
	Vradi04g00002788	AT1G56720.3	serine/threonine-protein kinase, putative, expressed
	Vradi04g00002789	AT1G09430.1	ATP-grasp domain containing protein, expressed
	Vradi04g00002790	AT3G07100.1	protein transport protein Sec24-like, putative, expressed
	Vradi04g00002791	AT5G66460.1	OsMan01 - Endo-Beta-Mannanase, expressed
	Vradi04g00002792	AT5G66460.1	OsMan01 - Endo-Beta-Mannanase, expressed
	Vradi04g00002793	AT1G56700.3	pyrrolidone-carboxylate peptidase, putative, expressed
	Vradi04g00002794	AT1G09420.1	glucose-6-phosphate 1-dehydrogenase, chloroplast precursor, putative, expressed

For Review Only

Height5-1	Vradi05g00000394	AT1G06840.1	expressed protein
SPM7-1	Vradi07g00000915	AT3G22970.1	plant-specific domain TIGR01615 family protein, expressed
	Vradi07g00000916	AT3G22960.1	pyruvate kinase, putative, expressed
	Vradi07g00000917	AT3G22942.1	AGG2, putative, expressed
	Vradi07g00000918	AT3G22950.2	ADP-ribosylation factor, putative, expressed
	Vradi07g00000919	AT3G22930.1	OsCam2 - Calmodulin, expressed
	Vradi07g00000920	AT5G51670.1	avr9/Cf-9 rapidly elicited protein, putative, expressed
FFT9-1	Vradi09g00001301	AT3G14470.1	disease resistance RPP13-like protein 1, putative, expressed
	Vradi09g00001302	AT1G69550.1	NBS-LRR disease resistance protein, putative, expressed
	Vradi09g00001303	AT3G50950.2	disease resistance protein RGA4, putative, expressed
	Vradi09g00001304	AT3G14470.1	disease resistance RPP13-like protein 1, putative, expressed
	Vradi09g00001305	AT1G69550.1	NBS-LRR disease resistance protein, putative, expressed
	Vradi09g00001306	AT3G14470.1	disease resistance RPP13-like protein 1, putative, expressed
Node11-1	Vradi11g00000546	AT5G38590.2	OsFBO11 - F-box and other domain containing protein, expressed
	Vradi11g00000547	AT2G19130.1	S-domain receptor-like protein kinase, putative, expressed
	Vradi11g00000548	AT1G34300.1	lectin protein kinase family protein, putative, expressed
	Vradi11g00000549	AT1G02740.1	chromatin modification-related protein EAF3, putative, expressed
	Vradi11g00000550	AT4G24210.1	OsFBX77 - F-box domain containing protein, expressed
	Vradi11g00000551	AT3G08820.1	pentatricopeptide, putative, expressed
	Vradi11g00000552	AT1G47200.1	MFP1 attachment factor 1, putative, expressed
	Vradi11g00000553	AT5G67320.1	F-box-like/WD repeat-containing protein TBL1XR1-A, putative, expressed
	Vradi11g00000554	AT5G43080.1	cyclin, putative, expressed
	Vradi11g00000555	AT5G43080.1	cyclin, putative, expressed
	Vradi11g00000556	AT1G54250.1	DNA-directed RNA polymerases I, II, and III subunit RPABC3, putative, expressed
	Vradi11g00000557	AT4G24204.3	zinc finger, C3HC4 type domain containing protein, expressed
	Vradi11g00000558	AT1G47240.1	natural resistance-associated macrophage protein, putative, expressed
	Vradi11g00000559	AT4G12080.1	AT hook motif domain containing protein, expressed
	Vradi11g00000560	AT4G08840.1	pumilio-family RNA binding repeat domain containing protein, expressed
	Vradi11g00000561	AT5G59845.1	GASR3 - Gibberellin-regulated GASA/GAST/Snakin family protein precursor, expressed
	Vradi11g00000562	AT5G43120.1	expressed protein
	Vradi11g00000563	AT4G24220.1	NAD dependent epimerase/dehydratase family protein, putative, expressed
	Vradi11g00000564	AT1G27695.2	glycine-rich protein, putative, expressed
	Vradi11g00000565	AT5G48810.1	cytochrome b5-like Heme/Steroid binding domain containing protein, expressed
	Vradi11g00000566	AT1G27700.1	syntaxin 6, N-terminal domain containing protein, expressed
	Vradi11g00000567	AT5G65860.2	ankyrin repeat domain containing protein, putative, expressed
	Vradi11g00000568	AT5G67360.1	OsSub30 - Putative Subtilisin homologue, expressed
	Vradi11g00000569	AT5G67360.1	OsSub30 - Putative Subtilisin homologue, expressed
	Vradi11g00000570	AT5G43160.1	expressed protein
	Vradi11g00000571	AT1G27730.1	ZOS12-09 - C2H2 zinc finger protein, expressed
	Vradi11g00000572	AT1G27450.1	phosphoribosyl transferase, putative, expressed

For Review Only

Vradi11g00000573	AT1G27752.1	CUE domain containing protein, expressed
Vradi11g00000574	AT5G43190.1	OsFBX153 - F-box domain containing protein, expressed
Vradi11g00000575	AT3G01400.1	spotted leaf 11, putative, expressed
Vradi11g00000576	AT1G27770.1	calcium-transporting ATPase, plasma membrane-type, putative, expressed
Vradi11g00000577	AT4G37630.2	cyclin, putative, expressed
Vradi11g00000578	AT1G27760.1	interferon-related developmental regulator, putative, expressed
Vradi11g00000579	AT5G58610.1	PHD finger family protein, putative, expressed
Vradi11g00000580	AT1G27840.1	WD domain, G-beta repeat domain containing protein, expressed
Vradi11g00000581	AT1G27460.1	NPGR1, putative, expressed
Vradi11g00000582	AT5G63905.1	expressed protein
Vradi11g00000583	AT1G59590.1	ZCF37, putative, expressed
Vradi11g00000584	AT5G22370.2	ATP binding protein, putative, expressed
Vradi11g00000585	AT1G10200.1	OsWLIM1 - LIM domain protein, putative actin-binding protein and transcription factor, expressed
Vradi11g00000586	AT2G32200.1	expressed protein
Vradi11g00000587	AT1G65550.1	nucleobase-ascorbate transporter, putative, expressed
Vradi11g00000588	AT4G38320.1	haemolysin-III, putative, expressed
Vradi11g00000589	AT4G37680.2	haemolysin-III, putative, expressed
Vradi11g00000590	AT4G38290.1	haemolysin-III, putative, expressed
Vradi11g00000591	AT1G58460.1	expressed protein
Vradi11g00000592	AT1G10130.1	calcium-transporting ATPase 3, endoplasmic reticulum-type, putative, expressed
Vradi11g00000593	AT5G09640.1	OsSCP60 - Putative Serine Carboxypeptidase homologue, expressed
Vradi11g00000594	AT1G58420.1	expressed protein
Vradi11g00000595	AT1G08790.1	expressed protein
Vradi11g00000596	AT2G01410.1	expressed protein
Vradi11g00000597	AT1G27480.1	lecithin cholesterol acyltransferase, putative, expressed
Vradi11g00000598	AT3G49810.1	U-box domain containing protein, expressed
Vradi11g00000599	AT1G10720.1	BSD domain containing protein, expressed
Vradi11g00000600	AT1G10150.1	PHLOEM 2-LIKE A10, putative, expressed
Vradi11g00000601	AT4G10810.1	expressed protein
Vradi11g00000602	AT1G59520.1	CW7, putative, expressed
Vradi11g00000603	AT1G75390.1	CPuORF1 - conserved peptide uORF-containing transcript, expressed
Vradi11g00000604	AT1G75390.1	CPuORF1 - conserved peptide uORF-containing transcript, expressed
Vradi11g00000605	AT5G26820.1	expressed protein
Vradi11g00000606	AT1G22680.1	hypothetical protein
Vradi11g00000607	AT3G45140.1	lipoxygenase, putative, expressed
Vradi11g00000608	AT1G58470.1	RNA recognition motif containing protein, putative, expressed
Vradi11g00000609	AT4G37750.1	AP2-like ethylene-responsive transcription factor AINTEGUMENTA, putative, expressed
Vradi11g00000610	AT4G07960.1	CSLC7 - cellulose synthase-like family C, expressed
Vradi11g00000611	AT1G58440.1	squalene monooxygenase, putative, expressed
Vradi11g00000612	AT4G07990.1	heat shock protein DnaJ, putative, expressed

For Review Only

Vradi11g00000613	AT5G65810.1	expressed protein
Vradi11g00000614	AT4G37770.1	aminotransferase, classes I and II, domain containing protein, expressed
Vradi11g00000615	AT3G54510.2	early-responsive to dehydration protein-related, putative, expressed
Vradi11g00000616	AT3G49690.1	MYB family transcription factor, putative, expressed
Vradi11g00000617	AT1G10070.3	aminotransferase domain containing protein, putative, expressed
Vradi11g00000618	AT5G06710.1	homeobox associated leucine zipper, putative, expressed
Vradi11g00000619	AT1G58370.1	glycosyl hydrolase family 10 protein, putative, expressed
Vradi11g00000620	AT1G66590.1	CHCH domain containing protein, expressed
Vradi11g00000621	AT1G66590.2	CHCH domain containing protein, expressed
Vradi11g00000622	AT2G25900.2	zinc finger CCCH type family protein, putative, expressed
Vradi11g00000623	AT2G25930.1	ELF3 protein, putative, expressed
Vradi11g00000624	AT5G25950.1	carboxyl-terminal peptidase, putative, expressed
Vradi11g00000625	AT1G55340.2	expressed protein
Vradi11g00000626	AT4G32940.1	vacuolar-processing enzyme precursor, putative, expressed
Vradi11g00000627	AT4G32940.1	vacuolar-processing enzyme precursor, putative, expressed
Vradi11g00000628	AT3G20430.1	expressed protein
Vradi11g00000629	AT5G36250.1	protein phosphatase 2C, putative, expressed
Vradi11g00000630	AT4G32960.1	expressed protein
Vradi11g00000631	AT2G25970.1	KH domain-containing protein, putative, expressed
Vradi11g00000632	AT5G53370.1	pectinesterase, putative, expressed
Vradi11g00000633	AT2G25970.1	KH domain-containing protein, putative, expressed
Vradi11g00000634	AT5G11770.1	ubiquinone oxidoreductase, putative, expressed
Vradi11g00000635	AT5G11790.1	pollen-specific protein SF21, putative, expressed
Vradi11g00000636	AT4G32980.1	homeobox domain containing protein, expressed
Vradi11g00000637	AT1G66730.1	ATP dependent DNA ligase family protein, putative, expressed
Vradi11g00000638	AT2G26040.1	bet v I allergen family protein, putative, expressed
Vradi11g00000639	AT5G56840.1	MYB family transcription factor, putative, expressed
Vradi11g00000640	AT2G26080.1	glycine dehydrogenase, putative, expressed
Vradi11g00000641	AT2G26080.1	glycine dehydrogenase, putative, expressed
Vradi11g00000642	AT5G56860.1	GATA zinc finger domain containing protein, expressed
Vradi11g00000643	AT1G60960.1	metal cation transporter, putative, expressed
Vradi11g00000644	AT5G11870.2	expressed protein
Vradi11g00000645	AT5G11890.1	harpin-induced protein 1 domain containing protein, expressed
Vradi11g00000646	AT5G56450.1	mitochondrial carrier protein, putative, expressed
Vradi11g00000647	AT4G33040.1	OsGrx_A2 - glutaredoxin subgroup III, expressed
Vradi11g00000648	AT2G26150.1	heat stress transcription factor, putative, expressed
Vradi11g00000649	AT2G26170.1	cytochrome P450, putative, expressed
Vradi11g00000650	AT2G26170.2	cytochrome P450, putative, expressed
Vradi11g00000651	AT2G26180.1	IQ calmodulin-binding motif domain containing protein, expressed
Vradi11g00000652	AT1G14560.1	mitochondrial carrier protein, putative, expressed

For Review Only

Vradi11g00000653		
Vradi11g00000654	AT5G11950.2	uncharacterized protein PA4923, putative, expressed
Vradi11g00000655	AT5G11960.1	expressed protein
Vradi11g00000656	AT5G11970.1	expressed protein
Vradi11g00000657	AT3G13920.3	eukaryotic translation initiation factor 4A-1
Vradi11g00000658	AT1G54270.1	EIF4A-2
Vradi11g00000659	AT5G11990.1	expressed protein
Vradi11g00000660	AT5G12000.1	protein kinase domain containing protein, putative, expressed
Vradi11g00000661	AT5G12010.1	expressed protein
Vradi11g00000662	AT4G33100.1	expressed protein
Vradi11g00000663	AT4G33090.1	puromycin-sensitive aminopeptidase, putative, expressed
Vradi11g00000664	AT1G72770.3	protein phosphatase 2C, putative, expressed
Vradi11g00000665	AT1G72770.2	protein phosphatase 2C, putative, expressed
Vradi11g00000666	AT4G14350.2	AGC_AGC_other_NDRh_TRCd.2 - ACG kinases include homologs to PKA, PKG and PKC, expressed
Vradi11g00000667	AT1G76940.2	RNA recognition motif containing protein, putative, expressed
Vradi11g00000668	AT4G33110.2	Cyclopropane-fatty-acyl-phospholipid synthase, putative, expressed
Vradi11g00000669	AT5G26170.1	WRKY7, expressed
Vradi11g00000670	AT5G12020.1	hsp20/alpha crystallin family protein, putative, expressed
Vradi11g00000671	AT5G26180.2	NOL1/NOP2/sun family protein, putative, expressed
Vradi11g00000672	AT5G12020.1	hsp20/alpha crystallin family protein, putative, expressed
Vradi11g00000673	AT5G12020.1	hsp20/alpha crystallin family protein, putative, expressed
Vradi11g00000674	AT1G72820.1	mitochondrial carrier protein, putative, expressed
Vradi11g00000675	AT3G42790.1	PHD finger protein, putative, expressed
Vradi11g00000676	AT5G39710.1	Rf1, mitochondrial precursor, putative, expressed
Vradi11g00000677	AT4G35590.1	RWP-RK domain-containing protein, putative, expressed
Vradi11g00000678	AT5G12040.1	nitrilase, putative, expressed
Vradi11g00000679	AT3G13980.1	expressed protein
Vradi11g00000680	AT4G31170.3	serine/threonine-protein kinase HT1, putative, expressed
Vradi11g00000681	AT5G12080.3	uncharacterized mscS family protein, putative, expressed
Vradi11g00000682	AT2G06520.1	ultraviolet-B-repressible protein, putative, expressed
Vradi11g00000683	AT5G21430.1	heat shock protein DnaJ, putative, expressed
Vradi11g00000684	AT5G03680.1	expressed protein
Vradi11g00000685	AT1G56600.1	glycosyl transferase 8 domain containing protein, putative, expressed
Vradi11g00000686	AT4G25500.3	RNA recognition motif containing protein, expressed
Vradi11g00000687	AT3G14620.1	cytochrome P450 72A1, putative, expressed
Vradi11g00000688	AT5G21482.1	cytokinin dehydrogenase precursor, putative, expressed
Vradi11g00000689	AT5G21482.1	cytokinin dehydrogenase precursor, putative, expressed
Vradi11g00000690	AT4G33210.1	OsFBL52 - F-box domain and LRR containing protein, expressed
Vradi11g00000691	AT5G12020.1	hsp20/alpha crystallin family protein, putative, expressed
Vradi11g00000692	AT2G26340.1	expressed protein

For Review Only

Vradi11g00000693	AT1G02180.1	expressed protein
Vradi11g00000694	AT5G21910.1	expressed protein
Vradi11g00000695	AT5G21920.1	YGGT family protein, putative, expressed
Vradi11g00000696	AT5G21930.3	copper-transporting ATPase PAA1, putative, expressed
Vradi11g00000697	AT1G55770.1	invertase/pectin methylesterase inhibitor family protein, putative, expressed
Vradi11g00000698	AT2G26330.1	receptor-like protein kinase 5 precursor, putative, expressed
Vradi11g00000699	AT4G33200.1	myosin, putative, expressed
Vradi11g00000700	AT2G26300.1	G-protein alpha subunit, putative, expressed
Vradi11g00000701	AT5G21950.1	hydrolase protein, putative, expressed
Vradi11g00000702	AT5G21950.1	hydrolase protein, putative, expressed
Vradi11g00000703	AT2G05940.1	protein kinase APK1B, chloroplast precursor, putative, expressed
Vradi11g00000704	AT5G20650.1	ctr copper transporter family protein, putative, expressed
Vradi11g00000705	AT1G72510.2	expressed protein
Vradi11g00000706	AT2G26280.1	smr domain containing protein, expressed
Vradi11g00000707	AT4G33170.1	pentatricopeptide repeat protein PPR986-12, putative, expressed
Vradi11g00000708	AT1G01040.2	DCL2, putative, expressed
Vradi11g00000709	AT3G43920.1	DCL2, putative, expressed
Vradi11g00000710	AT2G26270.1	BRCA1 C Terminus domain containing protein, expressed
Vradi11g00000711	AT5G27640.1	eukaryotic translation initiation factor 3 subunit B, putative, expressed
Vradi11g00000712	AT2G26260.1	sterol-4-alpha-carboxylate 3-dehydrogenase, decarboxylating, putative, expressed
Vradi11g00000713	AT2G45700.1	DNA repair metallo-beta-lactamase family protein, expressed
Vradi11g00000714	AT1G74930.1	ethylene-responsive transcription factor, putative, expressed
Vradi11g00000715	AT2G26250.1	3-ketoacyl-CoA synthase 10, putative, expressed
Vradi11g00000716	AT5G21280.1	hydroxyproline-rich glycoprotein family protein
Vradi11g00000717	AT4G33160.1	OsFBX94 - F-box domain containing protein, expressed
Vradi11g00000718	AT2G24640.1	ubiquitin carboxyl-terminal hydrolase family protein, expressed
Vradi11g00000719	AT2G45590.1	protein kinase, putative, expressed
Vradi11g00000720	AT5G10720.1	histidine kinase, putative, expressed
Vradi11g00000721	AT4G10720.1	ankyrin repeat-containing protein, putative, expressed
Vradi11g00000722	AT5G10730.1	NAD dependent epimerase/dehydratase family protein, putative, expressed
Vradi11g00000723	AT5G57685.1	GDU1, putative, expressed
Vradi11g00000724	AT5G24930.1	CCT/B-box zinc finger protein, putative, expressed
Vradi11g00000725	AT4G31750.1	protein phosphatase 2C, putative, expressed
Vradi11g00000726	AT5G24970.1	ELMO/CED-12 family protein, putative, expressed
Vradi11g00000727	AT5G10745.1	expressed protein
Vradi11g00000728	AT2G45670.1	acyltransferase, putative, expressed
Vradi11g00000729	AT5G10750.1	EDR2, putative, expressed
Vradi11g00000730	AT4G31770.1	debranching enzyme 1
Vradi11g00000731	AT2G24820.1	pheophorbide a oxygenase, chloroplast precursor, putative, expressed
Vradi11g00000732	AT1G14180.2	mandelonitrile lyase, putative, expressed

For Review Only

Vradi11g00000733	AT2G24940.1	cytochrome b5-like Heme/Steroid binding domain containing protein, expressed
Vradi11g00000734	AT2G24960.2	expressed protein
Vradi11g00000735	AT4G31860.1	protein phosphatase 2C, putative, expressed
Vradi11g00000736	AT4G31850.1	PPR repeat domain containing protein, putative, expressed
Vradi11g00000737	AT4G31850.1	PPR repeat domain containing protein, putative, expressed
Vradi11g00000738	AT5G25080.1	sas10/Utp3 family protein, expressed
Vradi11g00000739	AT2G25060.1	plastocyanin-like domain containing protein, putative, expressed
Vradi11g00000740	AT5G10840.1	transmembrane 9 superfamily member, putative, expressed
Vradi11g00000741	AT4G31820.1	BTBN19 - Bric-a-Brac, Tramtrack, Broad Complex BTB domain with non-phototropic hypocotyl 3 NPH3 domain, expressed
Vradi11g00000742	AT5G11900.1	translation initiation factor SUI1, putative, expressed
Vradi11g00000743	AT3G23780.2	DNA-directed RNA polymerase subunit, putative, expressed
Vradi11g00000744	AT3G18090.1	DNA-directed RNA polymerase subunit, putative, expressed
Vradi11g00000745		
Vradi11g00000746	AT4G22030.1	F-box family protein, putative, expressed
Vradi11g00000747	AT5G10870.1	chorismate mutase, chloroplast precursor, putative, expressed
Vradi11g00000748	AT5G10870.1	chorismate mutase, chloroplast precursor, putative, expressed
Vradi11g00000749	AT3G18090.1	DNA-directed RNA polymerase subunit, putative, expressed
Vradi11g00000750	AT4G31805.1	expressed protein
Vradi11g00000751	AT1G80840.1	WRKY71, expressed
Vradi11g00000752	AT1G80840.1	WRKY71, expressed
Vradi11g00000753	AT5G25110.1	CAMK_KIN1/SNF1/Nim1_like.34 - CAMK includes calcium/calmodulin depedent protein kinases, expressed
Vradi11g00000754	AT5G52060.1	BAG domain-containing protein, putative, expressed
Vradi11g00000755	AT5G26600.2	aminotransferase, putative, expressed
Vradi11g00000756	AT4G31820.1	BTBN19 - Bric-a-Brac, Tramtrack, Broad Complex BTB domain with non-phototropic hypocotyl 3 NPH3 domain, expressed
Vradi11g00000757	AT5G25150.1	WD domain, G-beta repeat domain containing protein, expressed
Vradi11g00000758	AT4G31880.1	AT hook motif family protein, expressed
Vradi11g00000759	AT4G31880.2	AT hook motif family protein, expressed
Vradi11g00000760	AT2G25110.1	stromal cell-derived factor 2-like protein precursor, putative, expressed
Vradi11g00000761	AT4G31890.2	spotted leaf 11, putative, expressed
Vradi11g00000762	AT4G39952.1	pentatricopeptide, putative, expressed
Vradi11g00000763	AT2G25140.1	chaperone protein clpB 1, putative, expressed
Vradi11g00000764	AT1G24650.1	receptor protein kinase TMK1 precursor, putative, expressed
Vradi11g00000765	AT2G32070.1	CAF1 family ribonuclease containing protein, putative, expressed
Vradi11g00000766	AT2G25170.1	expressed protein
Vradi11g00000767	AT2G25180.1	two-component response regulator, putative, expressed
Vradi11g00000768	AT5G10970.1	ZOS3-07 - C2H2 zinc finger protein, expressed
Vradi11g00000769	AT5G25170.1	ethylene-responsive element-binding protein, putative, expressed
Vradi11g00000770	AT5G11000.1	expressed protein

For Review Only

Vradi11g00000771	AT5G25190.1	AP2 domain containing protein, expressed
Vradi11g00000772	AT4G31985.1	60S ribosomal protein L39, putative, expressed
Vradi11g00000773	AT2G25220.1	protein kinase family protein, putative, expressed
Vradi11g00000774	AT4G32020.1	serine/arginine repetitive matrix-like protein
Vradi11g00000775	AT1G29220.1	transcriptional regulator family protein, putative, expressed
Vradi11g00000776	AT2G34190.1	nucleobase-ascorbate transporter, putative, expressed
Vradi11g00000777	AT4G18710.1	CGMC_GSK.2 - CGMC includes CDA, MAPK, GSK3, and CLKC kinases, expressed
Vradi11g00000778	AT5G05830.1	zinc finger, C3HC4 type domain containing protein, expressed
Vradi11g00000779	AT4G18780.1	CESA4 - cellulose synthase, expressed
Vradi11g00000780	AT5G25210.1	hydrolase protein, putative, expressed
Vradi11g00000781	AT4G32030.1	hydrolase protein, putative, expressed
Vradi11g00000782	AT5G25220.2	homeobox protein knotted-1, putative, expressed
Vradi11g00000783	AT5G11070.1	expressed protein
Vradi11g00000784	AT1G80550.1	PPR repeat domain containing protein, putative, expressed
Vradi11g00000785	AT5G25260.1	nodulin, putative, expressed
Vradi11g00000786	AT2G25270.1	expressed protein
Vradi11g00000787	AT5G11090.1	serine-rich protein, putative, expressed
Vradi11g00000788	AT4G32060.1	EF hand family protein, putative, expressed
Vradi11g00000789	AT2G25290.3	octicosapeptide/Phox/Bem1p, putative, expressed
Vradi11g00000790	AT2G25290.3	octicosapeptide/Phox/Bem1p, putative, expressed
Vradi11g00000791	AT1G15750.4	lissencephaly type-1-like homology motif, putative, expressed
Vradi11g00000792	AT2G31170.1	cysteinyl-tRNA synthetase, putative, expressed
Vradi11g00000793	AT4G32140.1	thiamine-repressible mitochondrial transport protein THI74, putative, expressed
Vradi11g00000794	AT2G22125.1	expressed protein
Vradi11g00000795	AT5G11100.1	C2 domain containing protein, expressed
Vradi11g00000796	AT2G25320.1	expressed protein
Vradi11g00000797	AT5G03080.1	phosphatase, putative, expressed
Vradi11g00000798	AT1G62510.1	LTPL121 - Protease inhibitor/seed storage/LTP family protein precursor, putative, expressed
Vradi11g00000799	AT3G15920.1	phox domain-containing protein, putative, expressed
Vradi11g00000800	AT5G11160.1	phosphoribosyl transferase, putative, expressed
Vradi11g00000801	AT4G32250.3	protein kinase domain containing protein, expressed
Vradi11g00000802	AT4G32260.1	ATP synthase B chain, chloroplast precursor, putative, expressed
Vradi11g00000803	AT4G32270.1	expressed protein
Vradi11g00000804	AT4G32272.1	transporter family protein, putative, expressed
Vradi11g00000805	AT4G32280.1	OsIAA17 - Auxin-responsive Aux/IAA gene family member, expressed
Vradi11g00000806	AT2G25430.1	clathrin assembly protein, putative, expressed
Vradi11g00000807	AT4G32295.2	kinase-inducible domain interacting 9, KIX9
Vradi11g00000808	AT3G11760.1	expressed protein
Vradi11g00000809	AT4G32300.1	lectin protein kinase family protein, putative, expressed
Vradi11g00000810	AT4G32320.1	peroxidase precursor, putative, expressed

For Review Only

Vradi11g00000811	AT4G32330.2	targeting protein for Xklp2, putative, expressed
Vradi11g00000812	AT2G25490.1	OsFBL7 - F-box domain and LRR containing protein, expressed
Vradi11g00000813	AT5G25360.2	expressed protein
Vradi11g00000814	AT3G15730.1	phospholipase D, putative, expressed
Vradi11g00000815	AT3G15730.1	phospholipase D, putative, expressed
Vradi11g00000816	AT5G25390.2	AP2 domain containing protein, expressed
Vradi11g00000817	AT3G49500.1	RNA-dependent RNA polymerase, putative, expressed
Vradi11g00000818	AT3G47650.1	bundle sheath defective protein 2, putative, expressed
Vradi11g00000819	AT5G11170.1	UAP56A
Vradi11g00000820	AT4G32390.1	domain of unknown function, DUF250 domain containing protein, expressed
Vradi11g00000821	AT1G26900.1	pentatricopeptide, putative, expressed
Vradi11g00000822	AT5G25400.1	domain of unknown function, DUF250 domain containing protein, expressed
Vradi11g00000823	AT5G66520.1	pentatricopeptide, putative, expressed
Vradi11g00000824	AT2G18660.1	beta-expansin precursor, putative, expressed
Vradi11g00000825	AT4G32400.1	mitochondrial carrier protein, putative, expressed
Vradi11g00000826	AT4G32410.1	CESA1 - cellulose synthase, expressed
Vradi11g00000827	AT5G11270.1	homeobox domain containing protein, expressed
Vradi11g00000828	AT5G11280.1	expressed protein
Vradi11g00000829	AT2G28580.1	plant protein of unknown function domain containing protein, expressed
Vradi11g00000830	AT2G28580.1	plant protein of unknown function domain containing protein, expressed
Vradi11g00000831	AT2G28580.1	plant protein of unknown function domain containing protein, expressed

For Review Only

Supplementary table 9. Plant materials for genetic and phenotypic variations. AFG: Afghanistan, AUS: Australia, BGD: Bangladesh, CHN: China, IDN: Indonesia, IRN: Iran, KHM: Cambodia, KOR: Korea, MMR: Myanmar, NPL: Nepal, PAK: Pakistan, PHL: Philippines, PRK: North Korea, SGP: Singapore, THA: Thailand, TKY: Turkey, TWN: Taiwan, UNK: Unknown, UZB: Uzbekistan, VNM: Vietnam.

Accession Name	Genebank Code	Country of Origin	Wild/ Cultivar	Accession Name	Genebank Code	Country of Origin	Wild/ Cultivar
V01673	K024062	AFG	Cultivated		182273	KOR	Cultivated
JP229241		AFG	Cultivated		183235	KOR	Cultivated
JP31324		AFG	Cultivated		183264	KOR	Cultivated
Celera	183789	AUS	Cultivated		185570	KOR	Cultivated
HERNITAGERS	K024067	AUS	Cultivated		185575	KOR	Cultivated
JP229130		BGD	Cultivated		185576	KOR	Cultivated
Vo1301	163175	CHN	Cultivated		191127	KOR	Cultivated
CHN-LJR-2000-35	212108	CHN	Cultivated	Original Tashkent 1978	199273	KOR	Cultivated
ML-9	K130624	CHN	Cultivated		212101	KOR	Cultivated
VC-2307A	K130631	CHN	Cultivated		216796	KOR	Cultivated
R-288-8	K130632	CHN	Cultivated	KHM-LWJ-2011-10	K176373	KOR	Cultivated
KJA17	K136410	CHN	Cultivated	JP229109		KOR	Cultivated
K163475	K163475	CHN	Cultivated	RUS-NYW-2000-201	K005410	MDA	Cultivated
CHN-2010-18	K166126	CHN	Cultivated	K001419	K001419	MMR	Cultivated
CHN-2010-19	K166127	CHN	Cultivated	CN 72	K165772	MMR	Cultivated
CHN-2010-20	K166128	CHN	Cultivated	VC 6141-54	K165773	MMR	Cultivated
	K175297	CHN	Cultivated	VC 6368-46-40	K165775	MMR	Cultivated
	K175298	CHN	Cultivated	VC 6173-B-10	K165776	MMR	Cultivated
CHN-PMW-2011-4	K175546	CHN	Cultivated	VC 6173C	K165781	MMR	Cultivated
JP229144		CHN	Cultivated	VC 12-3-4A	K165782	MMR	Cultivated
JP229145		CHN	Cultivated	Siraha Local-2	136322	NPL	Cultivated
JP229215		CHN	Cultivated		IT109327	NPL	Cultivated

For Review Only

JP229216		CHN	Cultivated	Pakistan	154087	PAK	Cultivated
V01471	K024060	IDN	Cultivated	Vo3484	163234	PAK	Cultivated
Arta Moseng		IDN	Cultivated	JP103138-1		PAK	Cultivated
Si Walik		IDN	Cultivated	JP103138-2		PAK	Cultivated
Lok Madura		IDN	Cultivated	JP99066		PAK	Cultivated
Arta Item		IDN	Cultivated	V01946A-Y	189516	PHL	Cultivated
Arta ijo		IDN	Cultivated	ACC6	201172	PHL	Cultivated
Manyar		IDN	Cultivated	ACC11	201177	PHL	Cultivated
Bhakti		IDN	Cultivated	Acc. 363	807483	PHL	Cultivated
No 129		IDN	Cultivated	EG MG-13	807492	PHL	Cultivated
Nuri		IDN	Cultivated	Dull Yellow	807498	PHL	Cultivated
Kenari		IDN	Cultivated	NATIVE COLLECTION	IT201188/701 320	PHL	Cultivated
Betet		IDN	Cultivated	EG-MG-60	K024059	PHL	Cultivated
Gelatik		IDN	Cultivated	CES-J-24	K130625	PHL	Cultivated
Parkit		IDN	Cultivated	FG-MG-1743	K130627	PHL	Cultivated
Merpati		IDN	Cultivated	CES-87	K130634	PHL	Cultivated
Camar		IDN	Cultivated	Bohabe yellow mongo		PHL	Cultivated
Merak		IDN	Cultivated	JP231194		PHL	Cultivated
Calon haji 1a		IDN	Cultivated	VIR 6559	K173279	PRK	Cultivated
Samsek-a		IDN	Cultivated	VIR 6560	K173280	PRK	Cultivated
Calon haji ongko		IDN	Cultivated	YV 542	K169726	SGP	Cultivated
Plastik		IDN	Cultivated	JP229096		THA	Cultivated
Lok Ps Jailolo		IDN	Cultivated	JP229097		THA	Cultivated
FOrewehal		IDN	Cultivated	JP229098		THA	Cultivated
Fore Lotu		IDN	Cultivated	JP229099		THA	Cultivated
Bue bura		IDN	Cultivated	JP231216		THA	Cultivated
Lok Kab Borong A		IDN	Cultivated	JP231220		THA	Cultivated

For Review Only

Lok Kota Kumbah A	IDN	Cultivated	CN900001		THA	Cultivated
Nilon	IDN	Cultivated	CN900002		THA	Cultivated
Lok Mutoha M-1	IDN	Cultivated	CN900004		THA	Cultivated
Lok Abuki	IDN	Cultivated	CN900005		THA	Cultivated
Lok Majenang A	IDN	Cultivated	CN900007		THA	Cultivated
Lok Pangalengan	IDN	Cultivated	CN900008		THA	Cultivated
Lok Tarogong	IDN	Cultivated	CN900014		THA	Cultivated
Mentik hitam	IDN	Cultivated	CN900015		THA	Cultivated
PB-1 (benggolo Puti)	IDN	Cultivated	V01122B-G	189472	TKY	Cultivated
Lok Kudus	IDN	Cultivated		104747	TWN	Cultivated
Lok Ngawi	IDN	Cultivated	VC1089A	182225	TWN	Cultivated
Lok Pemeungpeuk	IDN	Cultivated	VC2768B	182247	TWN	Cultivated
Lok Bungbulang	IDN	Cultivated	VC3890B	182296	TWN	Cultivated
Lok Kupang	IDN	Cultivated	NCM-1	K130648	TWN	Cultivated
Butek Surade	IDN	Cultivated	JP99049		TWN	Cultivated
Fore Belu	IDN	Cultivated	VC3566-B-2-1-3	145301	UNK	Cultivated
Perkutut	IDN	Cultivated	Yellowgram	154085	UNK	Cultivated
Tecer Hitam	IDN	Cultivated		182212	UNK	Cultivated
Lok Sampang 1	IDN	Cultivated	VC3523	182255	UNK	Cultivated
Lima-1	IDN	Cultivated		183263	UNK	Cultivated
Lok Jerowaru	IDN	Cultivated	Acc. 7861	801374	UNK	Cultivated
Kh.50 hari (L.insana)	IDN	Cultivated	P-3-A-40	K130639	UNK	Cultivated
Lokal Landa Baru	IDN	Cultivated	P-69-319	K130644	UNK	Cultivated
Lokal Mutaha K2	IDN	Cultivated	P-4-44	K130646	UNK	Cultivated
Lokal Garut M	IDN	Cultivated	Local	K130649	UNK	Cultivated
Mentik Coklat	IDN	Cultivated	Lokal	K130650	UNK	Cultivated
Lok. Pasar Welahan	IDN	Cultivated	Local	K130652	UNK	Cultivated
Lokal Sidamulih	IDN	Cultivated	MYS-PYJ-2007-55	K131569	UNK	Cultivated

For Review Only

Kuyak		IDN	Cultivated	M135	M135	UNK	Cultivated
RR-2		IDN	Cultivated	M136	M136	UNK	Cultivated
Lok. Garut		IDN	Cultivated			UNK	Cultivated
Lok Puda 1		IDN	Cultivated			UNK	Cultivated
Lok Galis		IDN	Cultivated			UNK	Cultivated
Arta Koneng		IDN	Cultivated			UNK	Cultivated
Lok. Pasanggaran		IDN	Cultivated			UNK	Cultivated
Lok. Jonggat		IDN	Cultivated			UNK	Cultivated
Fore Modok		IDN	Cultivated			UNK	Cultivated
ArthaZatim		IDN	Cultivated	V3686	154080	USA	Cultivated
Fue Nutu		IDN	Cultivated	V03720B-G	189553	USA	Cultivated
Foe Nutu		IDN	Cultivated	UZB-Shahrisabz-2011-21	K187555	UZB	Cultivated
Kambe Morowisa		IDN	Cultivated	UZB-Shahrisabz-2011-33	K187567	UZB	Cultivated
Kambe Kulita Kokana		IDN	Cultivated	UZB-Khazarbag-2011-60	K187592	UZB	Cultivated
Kabe Mor		IDN	Cultivated	Zilola		UZB	Cultivated
Lok. Bajawa Ngada		IDN	Cultivated	Durdona		UZB	Cultivated
Lok. Ps. Embai Golewa Barat		IDN	Cultivated	Turon		UZB	Cultivated
JP229233		IDN	Cultivated	JP78939		VNM	Cultivated
Lok NTB		IDN	Cultivated	JP229290		AUS	Wild
Antap ongko 1a		IDN	Cultivated	M146	M146	UNK	Wild
Lokal Bima 2a		IDN	Cultivated	M151	M151	UNK	Wild
JP229163		IND	Cultivated	M153	M153	UNK	Wild
JP229177		IND	Cultivated	W017	W017	UNK	Wild
JP229175		IND	Cultivated	W063	W063	UNK	Wild
JP229211		IND	Cultivated	W065	W065	UNK	Wild
JP229190		IND	Cultivated	W073	W073	UNK	Wild
JP231223		IND	Cultivated	W077	W077	UNK	Wild
V1153	154078	IRN	Cultivated	W080	W080	UNK	Wild

For Review Only

Vo5551	163280	IRN	Cultivated	W083	W083	UNK	Wild
V03827A-G	189554	IRN	Cultivated	W101	W101	UNK	Wild
JP229254		IRN	Cultivated	W105	W105	UNK	Wild
JP229257		IRN	Cultivated	W109	W109	UNK	Wild
JP229263		IRN	Cultivated	W111	W111	UNK	Wild
JP31331		IRN	Cultivated	W115	W115	UNK	Wild
KHM-LWJ-2011-9	K176372	KHM	Cultivated	W116	W116	UNK	Wild
KHM-LWJ-2011-12	K176375	KHM	Cultivated	W128	W128	UNK	Wild
	K191035	KHM	Cultivated	W130	W130	UNK	Wild
V03538B-G	189552	KNY	Cultivated	W131	W131	UNK	Wild
	26018	KOR	Cultivated	W133	W133	UNK	Wild
	26062	KOR	Cultivated	W134	W134	UNK	Wild
	26252	KOR	Cultivated	W138	W138	UNK	Wild
	102800	KOR	Cultivated	W141	W141	UNK	Wild
	102855	KOR	Cultivated	W146	W146	UNK	Wild
	103400	KOR	Cultivated	W147	W147	UNK	Wild
	103418	KOR	Cultivated	W148	W148	UNK	Wild
	103835	KOR	Cultivated	W158	W158	UNK	Wild
	104183	KOR	Cultivated	W159	W159	UNK	Wild
	105626	KOR	Cultivated	W162	W162	UNK	Wild
	105639	KOR	Cultivated	W168	W168	UNK	Wild
	111041	KOR	Cultivated	W169	W169	UNK	Wild
	112822	KOR	Cultivated	W172	W172	UNK	Wild
	138114	KOR	Cultivated	W174	W174	UNK	Wild
	162743	KOR	Cultivated	W176	W176	UNK	Wild
	162779	KOR	Cultivated	W177	W177	UNK	Wild
	162880	KOR	Cultivated	W182	W182	UNK	Wild
MBLS90-19	168064	KOR	Cultivated	W190	W190	UNK	Wild

For Review Only

MBLS90-30	168075	KOR	Cultivated	W191	W191	UNK	Wild
	175816	KOR	Cultivated	W192	W192	UNK	Wild
	180833	KOR	Cultivated	W203	W203	UNK	Wild
	180835	KOR	Cultivated	W204	W204	UNK	Wild
	181876	KOR	Cultivated	GP109		UNK	*Vigna glabrescens*

For Review Only

Supplementary table 10. Summary of variants obtained from GBS of 276 wild and cultivated mungbeans.

Genome total length	475,779,714
Genome effective length	474,607,383
Sequencing read number	
Minimum	660,387
Maximum	14,258,096
Median	2,628,462
Number of aligned reads	
Minimum	624,092 (48.4%)
Maximum	4,671,112 (95.4%)
Median	2,162,549 (79.7%)
Variant rate	1 variant every 15,237 bases
Number of variants	30,424
**Location relative to genes**	
Downstream	13,449
Exon	20,338
Intergenic	2,957
Intron	4,482
Splice site acceptor	19
Splice site donor	31
Splice site region	398
Upstream	9,920
UTR 3’	1,083
UTR 5’	1,411

For Review Only

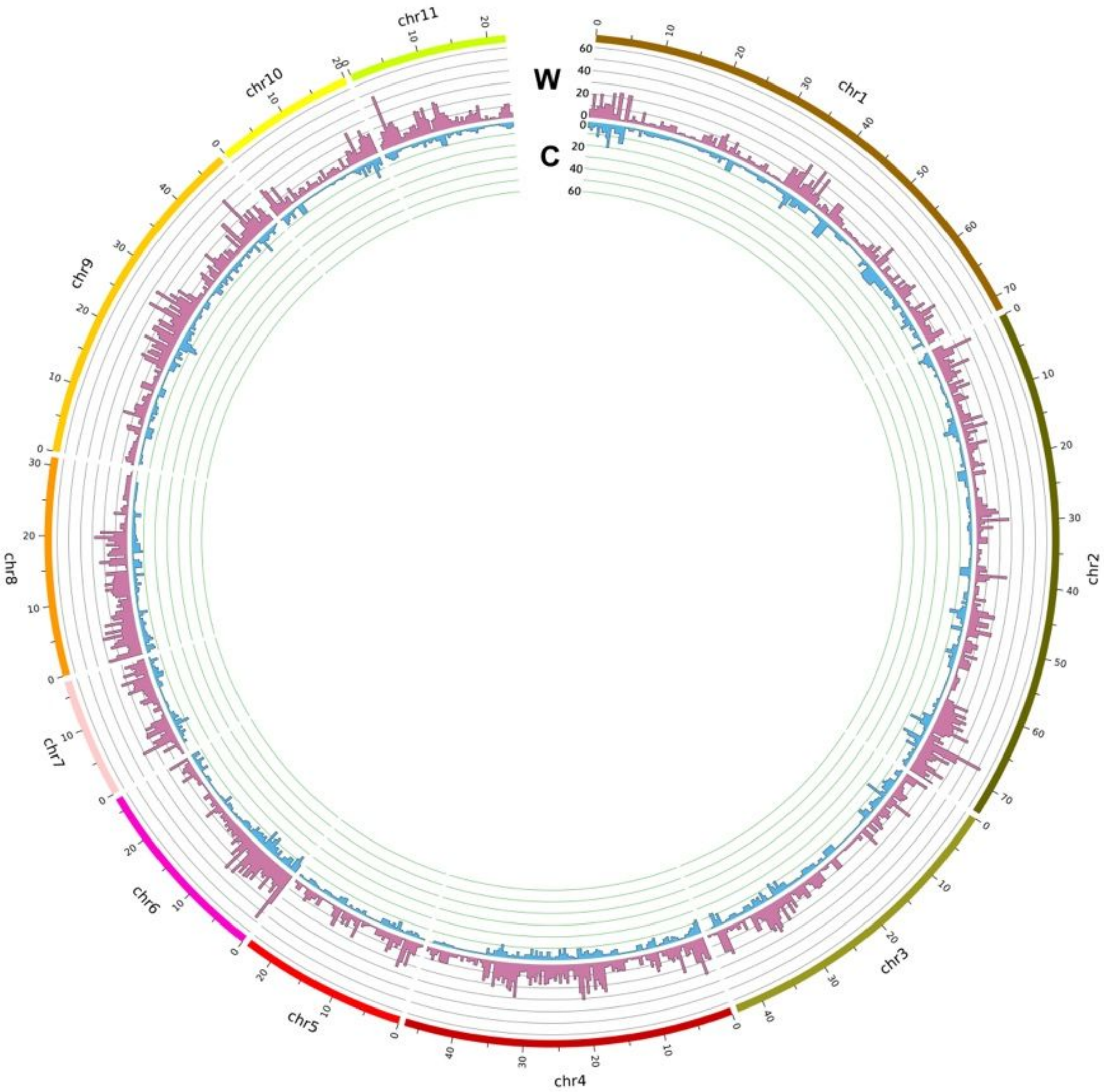

Supplementary figure 6. Distribution of the number of sequence variants in 400,000 bp intervals on the 11 chromosomes of mungbean. Outer and inner circles indicate sequence variants of wild and cultivated mungbeans, respectively.

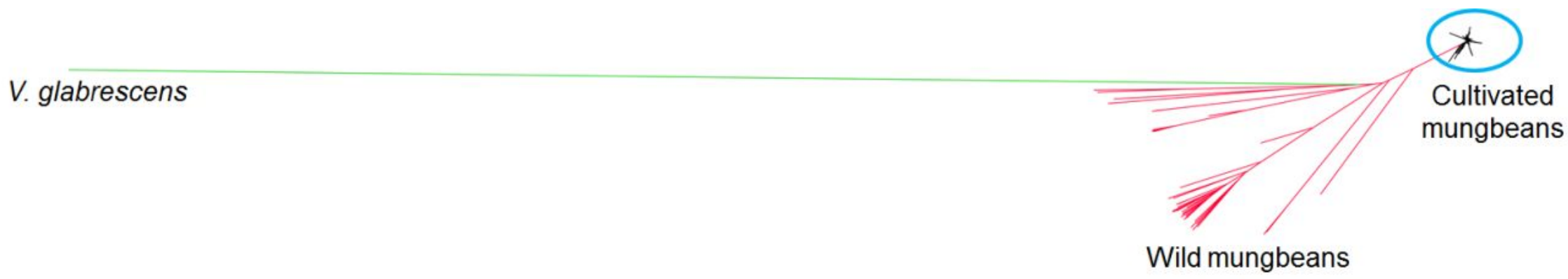

Supplementary figure 7. Neighbor joining tree for 276 mungbean accessions from 23 countries. Blue circle includes 233 cultivated mungbeans. Red branches indicate the 42 wild mungbeans, while green branch indicates *V. glabrescens* as an outgroup.

For Review Only

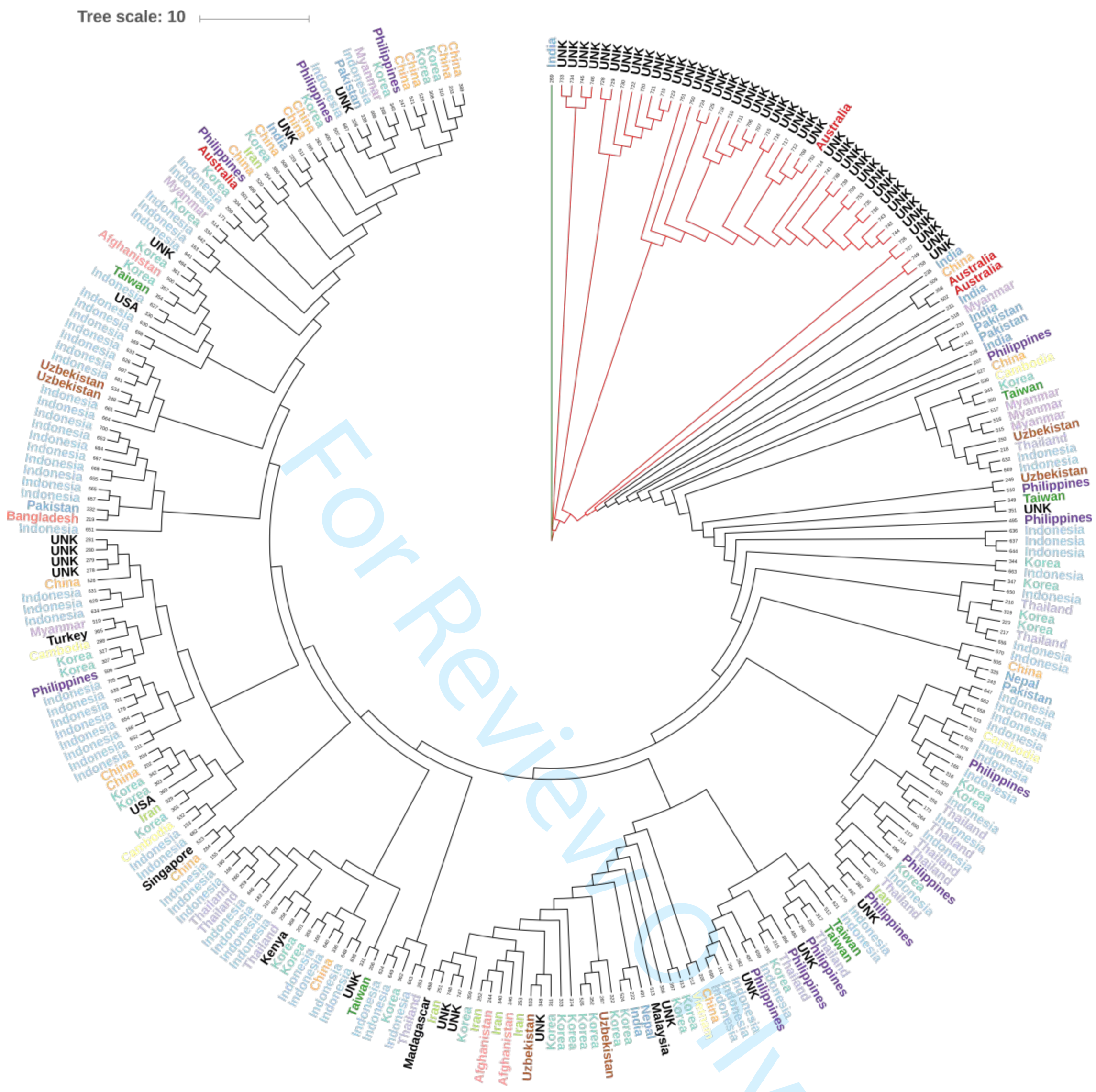

For Review Only

Supplementary figure 8. Cladogram of phylogenetic relationship among mungbean accessions in the context of their origins.

Red and black branches indicate wild and cultivated accessions, respectively, and the black branch closest to the red branches is JP231223 from India. Unk denotes accessions with no known country of origin data. Green branch indicates the outgroup, *V. glabrescens.*

543 Supplementary table 11. List of selective sweep regions.

Selective sweep	Start (Mbp)	End (Mbp)	Fst	XPCLR	The number of genes
chr1-1	21.4	21.5	0.35	6.03	11
chr1-2	26.1	26.2	0.93	6.00	8
chr1-3	49.5	49.6	0.52	18.50	2
chr1-4	55.9	56.0	0.95	8.36	6
chr1-5	61.5	61.6	0.39	6.40	8
chr2-1	11.5	11.6	0.36	15.66	4
chr2-2	20.0	20.1	0.94	6.64	7
chr2-3	20.5	20.6	0.39	6.16	8
chr2-4	21.1	21.2	0.33	6.53	4
chr2-5	23.9	24.0	0.44	6.29	5
chr2-6	29.2	29.3	0.38	6.30	13
chr2-7	29.3	29.4	0.48	8.44	6
chr2-8	34.9	35.0	0.57	6.17	2
chr2-9	40.6	40.7	0.44	6.30	2
chr2-10	41.4	41.5	0.36	6.70	2
chr2-11	49.4	49.5	0.42	6.58	1
chr2-12	56.4	56.5	0.38	8.32	3
chr3-1	10.7	10.8	0.96	7.78	5
chr4-1	7.0	7.1	0.34	20.74	10
chr4-2	11.3	11.4	0.40	6.70	5
chr4-3	15.0	15.1	0.33	6.23	14
chr4-4	15.2	15.3	0.44	7.17	8
chr4-5	15.6	15.7	0.89	7.31	6
chr4-6	37.7	37.8	0.42	6.33	5
chr5-1	4.0	4.1	0.61	6.84	12
chr5-2	13.7	13.8	0.63	15.94	7
chr6	24.6	24.7	0.34	6.90	6
chr7-1	11.9	12.0	0.33	21.50	10
chr7-2	15.7	15.8	0.88	17.72	4
chr7-3	16.4	16.5	0.41	6.70	10
chr8-1	17.3	17.4	0.35	6.85	2
chr8-2	23.8	23.9	0.59	8.59	10
chr9-1	4.6	4.7	0.45	6.27	2
chr9-2	39.1	39.2	0.41	6.51	4
chr10-1	6.4	6.5	0.45	7.09	7
chr11-1	15.5	15.6	0.35	11.28	5

For Review Only

Supplementary table 12. The list of genes in selective sweep regions. Soybean QTLs were searched on the genomic regions in soybean with syntenic relationship with the genomic regions of mungbean selective sweep. Arabidopsis orthologous genes were identified by blastp. (SDS: sudden death syndrome, SCN: soybean cyst nematode)

Selective sweep	Gene name	Soybean QTLs	*At* orthologs	Description
Chr1-1	Vradi01g00001233	*Seed_weight_35-4* *mqPlant_height_006*	AT2G27310.1	OsFBX396 - F-box domain containing protein, expressed
	Vradi01g00001234		AT5G22320.1	leucine rich repeat domain containing protein, putative, expressed
	Vradi01g00001235		AT1G08370.1	mRNA-decapping enzyme, putative, expressed
	Vradi01g00001236		AT2G27370.1	membrane associated DUF588 domain containing protein, putative, expressed
	Vradi01g00001237		AT5G22350.1	nucleoside-diphosphate-sugar epimerase, putative, expressed
	Vradi01g00001238		AT3G10230.2	lycopene beta cyclase, chloroplast precursor, putative, expressed
	Vradi01g00001239		AT5G22380.1	no apical meristem protein, putative, expressed
	Vradi01g00001240		AT2G27385.1	POEI29 - Pollen Ole e I allergen and extensin family protein precursor, putative, expressed
	Vradi01g00001241		AT5G09225.1	expressed protein
	Vradi01g00001242		AT2G41700.2	ABC transporter, ATP-binding protein, putative, expressed
	Vradi01g00001243		AT2G27450.1	N-carbamoylputrescine amidase, putative, expressed
Chr1-2	Vradi01g00001518	*mqPlant_height_003* *SCN_19-4* *SCN_37-3* *Branching_3-5* *SDS_13-9* *Seed_weight_per_plant_6-4*	AT1G65660.1	pre-mRNA-splicing factor SLU7, putative, expressed
	Vradi01g00001519		AT1G65660.1	
	Vradi01g00001520		AT1G07110.1	6PF-2-K/Fru-2,6-P2ASE liver isozyme, putative, expressed
	Vradi01g00001521		AT3G16720.1	zinc finger, C3HC4 type domain containing protein, expressed
	Vradi01g00001522		AT3G05200.1	zinc finger, C3HC4 type domain containing protein, expressed
	Vradi01g00001523		AT2G30080.1	metal cation transporter, putative, expressed

For Review Only

	Vradi01g00001524		AT3G25855.1	expressed protein
	Vradi01g00001525		AT4G32060.2	EF hand family protein, putative, expressed
Chr1-3	Vradi01g00002538		AT3G02510.1	Regulator of chromosome condensation domain containing protein, expressed
	Vradi01g00002539		AT5G53390.1	wax synthase, putative, expressed
Chr1-4	Vradi01g00002865		AT2G33420.1	expressed protein
	Vradi01g00002866		AT3G60470.1	plant protein of unknown function domain containing protein, expressed
	Vradi01g00002867		AT5G06540.1	expressed protein
	Vradi01g00002868		AT4G37380.1	pentatricopeptide, putative, expressed
	Vradi01g00002869		AT5G11290.1	plant protein of unknown function domain containing protein, expressed
	Vradi01g00002870		AT2G33420.1	
Chr1-5	Vradi01g00003057		AT1G03840.1	ZOS1-05 - C2H2 zinc finger protein, expressed
	Vradi01g00003058		AT1G28480.1	OsGrx_S2 - glutaredoxin subgroup III, expressed
	Vradi01g00003059		AT1G75840.1	ras-related protein, putative, expressed
	Vradi01g00003060	*Flood_tolerance_7-1*	AT1G03860.3	prohibitin-2, putative, expressed
	Vradi01g00003061	*mqPlant_height_006*	AT3G12670.1	CTP synthase, putative, expressed
	Vradi01g00003062		AT3G54680.1	
	Vradi01g00003063		AT5G60860.1	ras-related protein, putative, expressed
	Vradi01g00003064		AT1G75840.1	
chr2-1	Vradi02g00000912	*Pod_dehiscence_2-1*	AT2G23790.1	E2F-related protein, putative, expressed
	Vradi02g00000913	*Seed_yield_31-9*	AT4G36810.1	polyprenyl synthetase, putative, expressed
	Vradi02g00000914	*SCN_40-3*	AT3G22150.1	pentatricopeptide, putative, expressed
	Vradi02g00000915	*BSR_3-1*	AT4G36800.2	ubiquitin-conjugating enzyme, putative, expressed
chr2-2	Vradi02g00001476		AT1G64940.1	cytochrome P450, putative, expressed
	Vradi02g00001477	*Plant_height_37-6*	AT2G21790.1	ribonucleoside-diphosphate reductase large subunit, putative, expressed
	Vradi02g00001478	*Pod_maturity_34-6*	AT2G35100.1	exostosin family domain containing protein, expressed
	Vradi02g00001479	*Pod_maturity_24-5*	AT1G31170.2	sulfiredoxin-1, putative, expressed
	Vradi02g00001480	*SDS_16-4*<br>*Node_number_6-1*	AT2G15430.1	DNA-directed RNA polymerase subunit, putative, expressed
	Vradi02g00001481		AT2G35110.1	NAP1, putative, expressed

For Review Only

	Vradi02g00001482		AT2G35110.2	
chr2-3	Vradi02g00001503		AT2G04340.1	expressed protein
	Vradi02g00001504		AT1G31260.1	metal cation transporter, putative, expressed
	Vradi02g00001505	*Plant_height_37-6*	AT1G31260.1	
	Vradi02g00001506	*Pod_maturity_34-6*	AT2G04340.1	
	Vradi02g00001507	*Pod_maturity_24-5* *SDS_16-4*	AT5G67460.1	glucan endo-1,3-beta-glucosidase-related, putative, expressed
	Vradi02g00001508	*Node_number_6-1*	AT3G09270.1	glutathione S-transferase, putative, expressed
	Vradi02g00001509		AT1G31280.1	PAZ domain-containing protein, putative, expressed
	Vradi02g00001510		AT4G13240.1	ATROPGEF7/ROPGEF7, putative, expressed
chr2-4	Vradi02g00001525		AT1G50575.1	possible lysine decarboxylase domain containing protein, expressed
	Vradi02g00001526		ATMG00580.1	NADH-ubiquinone oxidoreductase chain 4, putative, expressed
	Vradi02g00001527		AT1G26580.1	MYB family transcription factor, putative, expressed
	Vradi02g00001528			
chr2-5	Vradi02g00001625		AT1G26870.1	no apical meristem protein, putative, expressed
	Vradi02g00001626		AT1G14270.1	CAAX amino terminal protease family protein, putative, expressed
	Vradi02g00001627		AT1G26850.2	methyltransferase, putative, expressed
	Vradi02g00001628		AT4G21330.1	helix-loop-helix DNA-binding domain containing protein, expressed
	Vradi02g00001629		AT2G02950.1	expressed protein
chr2-6	Vradi02g00001901	*Seed_yield_22-11* *Seed_weight_15-1* *Seed_weight_34-15* *First_flower_22-2* *Total_growth_duration_1-4* *Flood_tolerance_4-1* *SDS_11-1*	AT3G30390.2	amino acid transporter, putative, expressed
	Vradi02g00001902		AT3G57360.1	expressed protein
	Vradi02g00001903		AT2G41600.3	mitochondrial glycoprotein, putative, expressed
	Vradi02g00001904		AT1G68930.1	pentatricopeptide, putative, expressed
	Vradi02g00001905		AT5G49610.1	OsFBX53 - F-box domain containing protein, expressed
	Vradi02g00001906		AT5G16970.1	NADP-dependent oxidoreductase, putative, expressed
	Vradi02g00001907		AT5G66520.1	pentatricopeptide, putative, expressed
	Vradi02g00001908		AT5G15050.1	N-acetylglucosaminyltransferase, putative, expressed
	Vradi02g00001909		AT1G52600.1	OsSigP4 - Putative Type I Signal Peptidase homologue; employs a putative Ser/His catalytic dyad, expressed

For Review Only

	Vradi02g00001910		AT3G57340.2	chaperone protein dnaJ 49, putative, expressed
	Vradi02g00001911		AT3G57340.2	
	Vradi02g00001912		AT2G26830.1	choline/ethanolamine kinase, putative, expressed
	Vradi02g00001913		AT2G15325.1	LTPL11 - Protease inhibitor/seed storage/LTP family protein precursor, expressed
chr2-7	Vradi02g00001914		AT2G15325.1	
	Vradi02g00001915		AT5G20070.1	hydrolase, NUDIX family protein, expressed
	Vradi02g00001916		AT5G49610.1	
	Vradi02g00001917		AT5G20150.1	SPX domain containing protein, putative, expressed
	Vradi02g00001918		AT1G15360.1	AP2 domain containing protein, expressed
	Vradi02g00001919		AT2G27170.2	chromosome segregation protein, putative, expressed
chr2-8	Vradi02g00002127		AT4G08540.1	expressed protein
	Vradi02g00002128		AT4G08540.1	
chr2-9	Vradi02g00002334		AT5G51810.1	gibberellin 20 oxidase 1, putative, expressed
	Vradi02g00002335		AT4G11820.1	hydroxymethylglutaryl-CoA synthase, putative, expressed
chr2-10	Vradi02g00002366	*Seed_weight_34-9*	AT5G17330.1	glutamate decarboxylase, putative, expressed
	Vradi02g00002367	*Branching_4-1*<br>*Japanese_beetle_resistance_1-4*<br>*Seed_weight_34-10*<br>*SCN_33-2*<br>*Pod_number_1-4*	AT2G21080.1	extracellular ligand-gated ion channel, putative, expressed
chr2-11	Vradi02g00002816		AT5G54440.1	expressed protein
chr2-12	Vradi02g00003063		AT5G11260.1	bZIP transcription factor domain containing protein, expressed
	Vradi02g00003064		AT1G80930.1	nucampholin, putative, expressed
	Vradi02g00003065		AT4G01020.1	pre-mRNA-splicing factor ATP-dependent RNA helicase DHX16, putative, expressed
chr3-1	Vradi03g00000738		AT5G18210.1	versicolorin reductase, putative, expressed
	Vradi03g00000739	*SCN_19-4*	AT2G40350.1	AP2 domain containing protein, expressed
	Vradi03g00000740	*SCN_20-4*	AT2G38360.1	prenylated rab acceptor, putative, expressed
	Vradi03g00000741	*Reproductive_period_1-3*	AT5G01650.1	macrophage migration inhibitory factor, putative, expressed

For Review Only

	Vradi03g00000742		AT3G51660.1	macrophage migration inhibitory factor, putative, expressed
	Vradi04g00000761		AT4G00660.2	
	Vradi04g00000762		AT4G00660.2	
	Vradi04g00000763		AT5G45500.2	disease resistance protein, putative, expressed
	Vradi04g00000764		AT5G45500.2	
	Vradi04g00000765		AT1G11910.1	aspartic proteinase oryzasin-1 precursor, putative, expressed
chr4-1	Vradi04g00000766		AT4G38260.1	Ser/Thr-rich protein T10 in DGCR region, putative, expressed
	Vradi04g00000767		AT3G50380.1	protein kinase domain containing protein, expressed
	Vradi04g00000768		ATCG01240.1	chloroplast 30S ribosomal protein S7, putative, expressed
	Vradi04g00000769		ATCG01250.1	NADPH-dependent oxidoreductase, putative, expressed
	Vradi04g00000770		ATCG00860.1	OsFtsH1 FtsH protease, homologue of AtFtsH1/5, expressed
	Vradi04g00001017		AT3G51800.3	peptidase, M24 family protein, putative, expressed
	Vradi04g00001018	*SCN_19-4*	AT3G08960.1	importin subunit beta, putative, expressed
chr4-2	Vradi04g00001019	*SCN_20-4*	AT3G08970.1	heat shock protein DnaJ, putative, expressed
	Vradi04g00001020	*Reproductive_period_1-3*	AT1G49900.1	ZOS4-09 - C2H2 zinc finger protein, expressed
	Vradi04g00001021		AT4G33070.1	pyruvate decarboxylase isozyme 2, putative, expressed
	Vradi04g00001196		AT2G41451.1	abscisic acid insensitive 8, putative, expressed
	Vradi04g00001197		AT2G41450.1	acetyltransferase, GNAT family, putative, expressed
	Vradi04g00001198		AT3G57180.1	GTP binding protein, putative, expressed
	Vradi04g00001199		AT2G41430.5	expressed protein
chr4-3	Vradi04g00001200	*SCN_33-7*	AT4G15770.1	60S ribosome subunit biogenesis protein NIP7, putative, expressed
	Vradi04g00001201		AT3G57170.1	N-acetylglucosaminyl transferase component, putative, expressed
	Vradi04g00001202		AT1G11130.1	STRUBBELIG-RECEPTOR FAMILY 3 precursor, putative, expressed
	Vradi04g00001203		AT5G51850.1	

For Review Only

	Vradi04g00001204		AT1G52820.1	oxidoreductase, 2OG-FeII oxygenase domain containing protein, putative, expressed
	Vradi04g00001205		AT1G52800.1	oxidoreductase, 2OG-FeII oxygenase domain containing protein, putative, expressed
	Vradi04g00001206		AT1G52790.1	oxidoreductase, 2OG-FeII oxygenase domain containing protein, putative, expressed
	Vradi04g00001207		AT4G33030.1	NAD dependent epimerase/dehydratase family protein, putative, expressed
	Vradi04g00001208		AT1G47128.1	oryzain beta chain precursor, putative, expressed
	Vradi04g00001209		AT5G04020.1	expressed protein
chr4-4	Vradi04g00001216	*SCN_33-7*	AT2G41250.1	rhythmically expressed gene 2 protein, putative, expressed
	Vradi04g00001217		AT2G39970.1	mitochondrial carrier protein, putative, expressed
	Vradi04g00001218		AT2G41290.1	strictosidine synthase, putative, expressed
	Vradi04g00001219		AT3G57030.1	strictosidine synthase, putative, expressed
	Vradi04g00001220		AT3G57040.1	OsRR4 type-A response regulator, expressed
	Vradi04g00001221		AT4G29060.2	elongation factor TS family protein, expressed
	Vradi04g00001222		AT3G10250.2	plant-specific domain TIGR01589 family protein, putative, expressed
	Vradi04g00001223		AT3G57062.2	
chr4-5	Vradi04g00001241	*SCN_33-7*	AT1G14840.2	ATMAP70 protein, putative, expressed
	Vradi04g00001242		AT5G13270.1	pentatricopeptide repeat protein PPR986-12, putative, expressed
	Vradi04g00001243		AT5G04130.1	DNA gyrase subunit B, chloroplast/mitochondrial precursor, putative, expressed
	Vradi04g00001244		AT5G63840.1	glycosyl hydrolase, family 31, putative, expressed
	Vradi04g00001245		AT1G80270.3	PPR repeat domain containing protein, putative, expressed
	Vradi04g00001246		AT3G07490.1	OsCML28 - Calmodulin-related calcium sensor protein, expressed
chr4-6	Vradi04g00003608		AT3G54700.1	inorganic phosphate transporter, putative, expressed
	Vradi04g00003609		AT4G14385.1	sarcoma antigen NY-SAR-91, putative, expressed
	Vradi04g00003610		AT2G31190.2	DUF647 domain containing protein, putative, expressed

For Review Only

	Vradi04g00003611		AT2G31180.1	MYB family transcription factor, putative, expressed
	Vradi04g00003612		AT5G13270.1	
chr5-1	Vradi05g00000315	*Canopy_wilt_6-3* *mqPlant_height_003* *Seed_weight_per_plant_6-4* *SCN_37-3*	AT1G66170.1	PHD-finger domain containing protein, putative, expressed
	Vradi05g00000316		AT1G66160.1	U-box protein CMPG1, putative, expressed
	Vradi05g00000317		AT1G66150.1	receptor protein kinase TMK1 precursor, putative, expressed
	Vradi05g00000318		AT1G66150.1	
	Vradi05g00000319		AT1G18910.1	E3 ubiquitin ligase, putative, expressed
	Vradi05g00000320		AT2G01820.1	receptor protein kinase TMK1 precursor, putative, expressed
	Vradi05g00000321		AT1G66150.1	
	Vradi05g00000322		AT1G66140.1	ZOS3-07 - C2H2 zinc finger protein, expressed
	Vradi05g00000323		AT5G48170.1	OsFBX77 - F-box domain containing protein, expressed
	Vradi05g00000324		AT5G16550.1	expressed protein
	Vradi05g00000325		AT5G16560.1	KANADI1, putative, expressed
	Vradi05g00000326		AT1G66080.1	expressed protein
chr5-2	Vradi05g00000765	*Plant_height_22-1*	AT3G26700.1	protein kinase, putative, expressed
	Vradi05g00000766		AT3G19330.1	DUF677 domain containing protein, putative, expressed
	Vradi05g00000767		AT3G02130.1	receptor-like protein kinase precursor, putative, expressed
	Vradi05g00000768		AT3G19340.1	expressed protein
	Vradi05g00000769		AT5G20090.2	UPF0041 domain containing protein, putative, expressed
	Vradi05g00000770		AT1G05560.1	UDP-glucoronosyl and UDP-glucosyl transferase domain containing protein, expressed
	Vradi05g00000771		AT4G14090.1	UDP-glucoronosyl and UDP-glucosyl transferase domain containing protein, expressed
chr6-1	Vradi06g00002026		AT5G53940.1	yippee zinc-binding protein, putative, expressed
	Vradi06g00002027		AT5G53940.1	
	Vradi06g00002028		AT3G15210.1	AP2 domain containing protein, expressed
	Vradi06g00002029		AT3G15190.1	ribosomal protein rpS20, putative, expressed
	Vradi06g00002030		AT5G53920.1	ribosomal protein L11 methyltransferase-related, putative, expressed

For Review Only

	Vradi06g00002031		AT5G37450.1	expressed protein
	Vradi07g00000951		AT2G25090.1	CAMK_KIN1/SNF1/Nim1_like.34 - CAMK includes calcium/calmodulin dependent protein kinases, expressed
	Vradi07g00000952		AT3G02645.1	plant protein of unknown function domain containing protein, expressed
	Vradi07g00000953	*Seed_weight_50-16*	AT1G71270.1	vacuolar protein sorting-associated protein 52, putative, expressed
chr7-1	Vradi07g00000954	*SCN_39-12*	AT4G00026.1	AGAP008572-PA, putative, expressed
	Vradi07g00000955	*Japanese_beetle_resistance_1-5*	AT1G17820.1	expressed protein
	Vradi07g00000956	*SDS_15-5*	AT1G73190.1	aquaporin protein, putative, expressed
	Vradi07g00000957		AT1G28150.1	expressed protein
	Vradi07g00000958		AT5G36970.1	harpin-induced protein 1 domain containing protein, expressed
	Vradi07g00000959		AT3G05070.1	coiled-coil domain-containing protein 12, putative, expressed
	Vradi07g00000960		AT1G17940.1	expressed protein
	Vradi07g00001316		AT2G32700.6	transcriptional corepressor LEUNIG, putative, expressed
chr7-2	Vradi07g00001317		AT2G32560.1	OsFBX5 - F-box domain containing protein, expressed
	Vradi07g00001318		AT2G32560.1	
	Vradi07g00001319		AT3G52990.1	pyruvate kinase, putative, expressed
	Vradi07g00001369		AT2G04842.1	threonyl-tRNA synthetase, putative, expressed
	Vradi07g00001370		AT1G74670.1	GASR5 - Gibberellin-regulated GASA/GAST/Snakin family protein precursor, expressed
	Vradi07g00001371		AT5G04010.1	expressed protein
	Vradi07g00001372		AT3G57190.1	peptide chain release factor 2, putative, expressed
chr7-3	Vradi07g00001373		AT3G10160.1	folylpolyglutamate synthase, mitochondrial precursor, putative, expressed
	Vradi07g00001374		AT2G41460.1	apurinic endonuclease-redox protein, putative, expressed
	Vradi07g00001375		AT5G26110.1	RIO1 family protein, expressed
	Vradi07g00001376		AT2G41490.1	UDP-N-acetylglucosamine--dolichyl-phosphate N-acetylglucosaminephosphotransferase, putative, expressed

For Review Only

	Vradi07g00001377		AT2G41500.1	WD domain, G-beta repeat domain containing protein, expressed
	Vradi07g00001378		AT3G54010.1	peptidyl-prolyl isomerase PASTICCINO1, putative, expressed
chr8-1	Vradi08g00001763	*Seed_weight_34-1*<br>*Seed_weight_36-1*<br>*Seed_weight_per_plant_6-5*<br>*SCN_19-4*<br>*Plant_height_37-3*	AT5G55760.1	transcriptional regulator Sir2 family protein, putative, expressed
	Vradi08g00001764		AT5G47650.1	hydrolase, NUDIX family, domain containing protein, expressed
chr8-2	Vradi08g00002230	*Seed_weight_34-1*<br>*Seed_weight_36-1*<br>*Seed_weight_per_plant_6-5*<br>*SCN_19-4*<br>*Plant_height_37-3*	AT5G55480.1	glycerophosphoryl diester phosphodiesterase family protein, putative, expressed
	Vradi08g00002231		AT5G05710.1	pleckstrin homology domain-containing protein, putative, expressed
	Vradi08g00002232		AT4G26690.1	glycerophosphoryl diester phosphodiesterase family protein, putative, expressed
	Vradi08g00002233		AT1G80010.1	FAR1 family protein, expressed
	Vradi08g00002234		AT1G80930.1	
	Vradi08g00002235		AT4G26690.1	
	Vradi08g00002236		AT5G55470.1	transporter, monovalent cation:proton antiporter-2 family, putative, expressed
	Vradi08g00002237		AT5G55410.1	LTPL42 - Protease inhibitor/seed storage/LTP family protein precursor, expressed
	Vradi08g00002238		AT5G54010.1	UDP-glucoronosyl and UDP-glucosyl transferase domain containing protein, expressed
	Vradi08g00002239		AT4G22360.1	SWIB/MDM2 domain containing protein, expressed
chr9-1	Vradi09g00000147	*Plant_height_42-1*	AT2G03050.1	mTERF domain containing protein, expressed
	Vradi09g00000148	*Bean_pyralid_1-2*	AT1G26770.1	expansin precursor, putative, expressed
chr9-2	Vradi09g00002655	*Seed_weight_47-2*<br>*Pod_number_11-1*	AT1G72510.2	expressed protein
	Vradi09g00002656		AT2G26280.1	smr domain containing protein, expressed
	Vradi09g00002657		AT2G36230.1	1-5-phosphoribosyl-5-5-phosphoribosylaminomethylideneaminoimidazole-4-carboxamide isomerase, chloroplast precursor, putative, expressed

For Review Only

	Vradi09g00002658		AT1G19210.1	ethylene-responsive transcription factor, putative, expressed
chr10-1	Vradi10g00000510		AT2G42110.1	expressed protein
	Vradi10g00000511	*Plant_height_33-3*	AT2G42130.4	expressed protein
	Vradi10g00000512	*Plant_height_34-3*	AT2G42140.1	VQ domain containing protein, putative, expressed
	Vradi10g00000513	*Seed_yield_13-1*	AT3G12120.2	fatty acid desaturase, putative, expressed
	Vradi10g00000514	*Seed_weight_15-6* *SDS_16-1*	AT5G06760.1	late embryogenesis abundant group 1, putative, expressed
	Vradi10g00000515	*Seed_yield_28-10* *Pod_maturity_28-1*	AT5G06770.1	zinc finger C-x8-C-x5-C-x3-H type family protein, expressed
	Vradi10g00000516		AT3G12810.1	expressed protein
chr11-1	Vradi11g00001425		AT5G04520.1	uncharacterized protein HI0077, putative, expressed
	Vradi11g00001426		AT3G52210.1	mRNA cap guanine-N7 methyltransferase 2, putative, expressed
	Vradi11g00001427		AT5G04550.1	avr9/Cf-9 rapidly elicited protein, putative, expressed
	Vradi11g00001428		AT5G53460.3	glutamate synthase, chloroplast precursor, putative, expressed
	Vradi11g00001429		AT5G06160.1	ZOS3-17 - C2H2 zinc finger protein, expressed

For Review Only

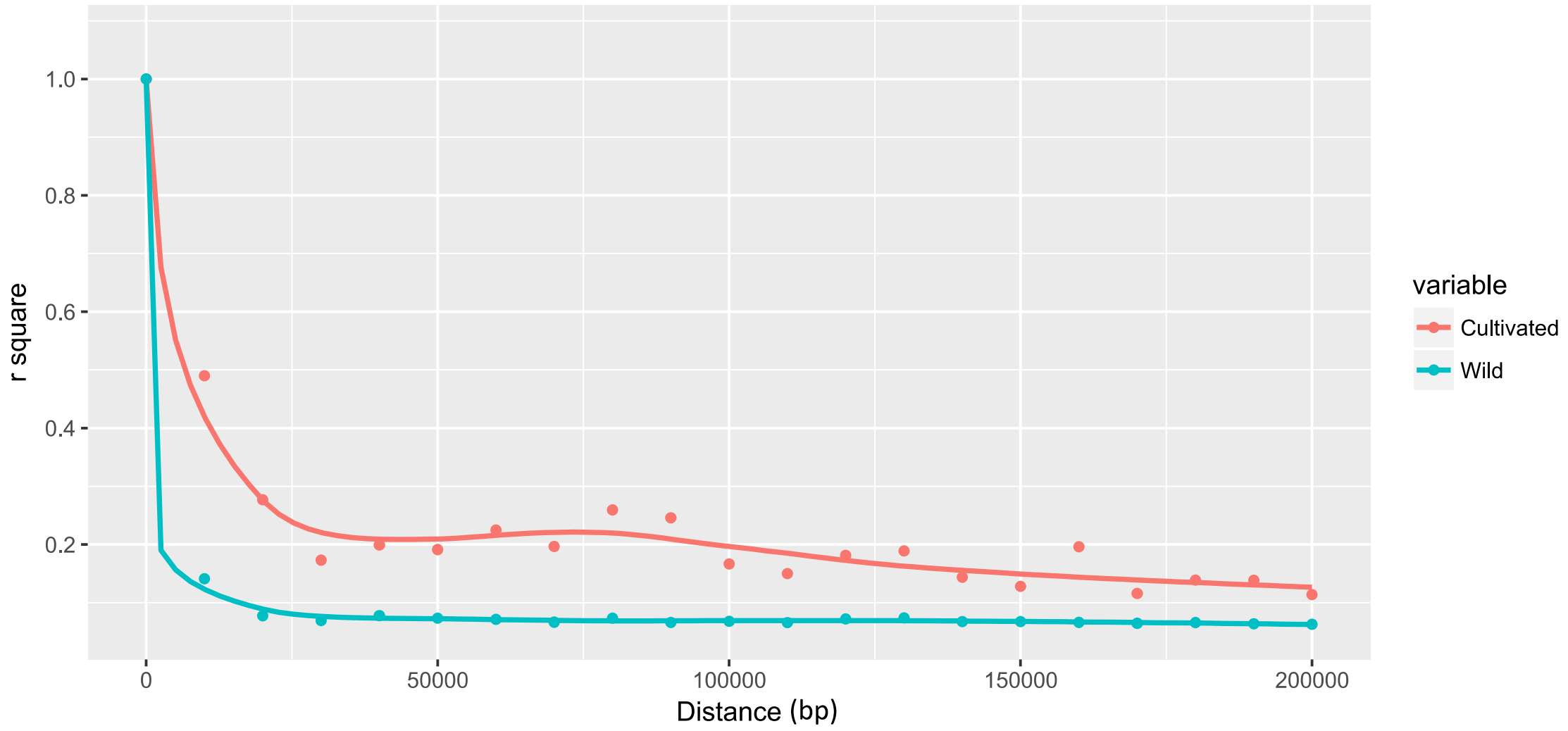

Supplementary figure 9. Average distance of LD decay in wild and cultivated mungbeans.

For Review Only

Supplementary table 13. List of nucleotide variations on candidate genes.
Upstream and downstream indicate ±2kbp of genic region.

Gene number		position	VC1973A	V2984	*V. reflexo-pilosa var. glabra*	*V. radiata var. sublobata*	*V. hirtella*	*V. trinervia*
Vradi03g00002294	upstream	41505695	TG	TGG	TGG	TGG	TGG	TGG
	upstream	41506316	AGG	AGGG	AGGG	AGGG	AGGG	AGGG
	downstream	41507888	TAAAAA	TAAAAAA	TAAAAAA	TAAAAAA	TAAAAAA	TAAAAAA
Vradi04g00002764	upstream	29612580	TCC	TCCC	TCCC	TCCC	TCCC	TCCC
	downstream	29610726	CAAA	CAAAA	CAAAA	CAAAA	CAAAA	CAAAA
	downstream	29610124	A	G	G	G	G	G
	downstream	29609961	ATTTT	ATTTTT	ATTTTT	ATTTTT	ATTTTT	ATTTTT
	downstream	29609761	T	TA	TA	TA	TA	TA
	downstream	29608979	C	CGGTAT	CGGTAT	CGGTAT	CGGTAT	CGGTAT
Vradi04g00002773	upstream	29654085	ATT	AT	AT	AT	AT	AT
	intron variant	29656262	GAA	GAAA	GAAA	GAAA	GAAA	GAAA
	frameshift	29656675	GTTT	GTT	GTT	GTT	GTT	GTT
	intron variant	29658028	GA	GATA	GATA	GATA	GATA	GATA
	intron variant	29658029	A	ATG	ATC	ATC	ATC	ATC
	frameshift	29658583	CAG	CAGTTGAG	CAGTTGAG	CAGTTGAG	CAGTTGAG	CAGTTGAG
Vradi05g00000394	upstream	4822912	AGTGT	AGT	-	AGT	AGT	-
	upstream	4822702	GAAA	GAA	-	GAA	GAA	-
	upstream	4821575	G	C	C	C	C	C
	intron variant	4820649	G	A	A	A	A	A
	5' UTR	4820586	T	TC	TC	TC	TC	TC
	intron variant	4820210	T	C	C	C	C	C

For Review Only

	5' UTR	4814554	CG	CGG	CGG	CGG	CGG	CGG
	intron variant	4813096	T	A	A	A	A	A
	missense	4812127	A	T	T	T	T	T
	intron variant	4812046	G	A	A	A	A	A
	intron variant	4811953	CA	CAA	CAA	CAA	CAA	CAA
	intron variant	4811438	A	C	C	C	C	C
	intron variant	4810085	G	A	A	A	A	A
	intron variant	4809856	T	TG	TG	TG	TG	TG
	intron variant	4809614	ATTTT	ATTTTT	-	ATTTTT	ATTTTT	-
	intron variant	4809143	G	A	A	A	A	A
	intron variant	4809057	G	A	A	A	A	A
	intron variant	4809024	A	G	G	G	G	G
	intron variant	4809023	G	A	A	A	A	A
	intron variant	4809003	T	A	A	A	A	A
	splice region	4808957	A	C	C	C	C	C
	stop lost	4808470	T	C	C	C	C	C
	downstream	4807966	T	G	G	G	G	G
	downstream	4807925	AT	ATT	ATT	ATT	ATT	ATT
	downstream	4807328	A	G	C	G	G	C
	downstream	4807266	ATTTT	ATTTTT	ATTTTT	ATTTTT	-	ATTTTT
	downstream	4807224	A	ATG	ATG	ATG	-	ATG
	downstream	4806868	CTTT	CTT	CTT	CTT	CTT	-
Vradi07g00000916	upstream	11564820	T	A	A	A	A	A
	upstream	11564755	A	C	C	C	C	-
	upstream	11564741	C	CA	CA	CA	CA	CA

For Review Only

upstream	11564490	GTT	GTTT	GTTT	GTTT	GTTT	GTTT
upstream	11564375	A	AG	AG	AG	AG	-
upstream	11564355	CGG	CGGG	CGGG	CGGG	CGGG	CGGG
upstream	11563809	AT	ATT	ATT	ATT	ATT	ATT
upstream	11563746	TG	TGG	TGG	TGG	TGG	TGG
upstream	11563540	GAAAAAA	GAAAAA	GAAAAA	GAAAAA	GAAAAA	-
upstream	11563469	A	C	C	C	C	C
intergenic	11563223	CT	CTGT	CTGT	CTGT	CTGT	CTGT
frameshift	11563102	T	TGA	TGA	TGA	TGA	TGA
frameshift	11563032	AGG	AGGG	AGGG	AGGG	AGGG	AGGG
frameshift	11562972	AGGGGG	AGGGGGG	AGGGGGG	AGGGGGG	AGGGGGG	AGGGGGG
intron	11562387	GTT	GT	GT	GT	GT	GT
frameshift	11562253	TCCCC	TCCCCC	TCCCCC	TCCCCC	TCCCCC	TCCCCC
intron	11561916	AC	ACC	ACC	ACC	ACC	ACC
intron	11561839	A	AT	AT	AT	AT	AT
intron	11561145	G	A	A	A	A	A
intron	11560817	G	A	C	A	C	C
intron	11560617	A	G	G	G	G	G
intron	11560616	T	A	A	A	A	A
intron	11560354	C	CT	CT	CT	CT	CT
intron	11560136	GTTT	GTT	GTT	GTT	GTT	GTT
intron	11560113	A	G	G	G	G	G
intron	11559855	A	AT	AT	AT	-	AT
intron	11559431	GTT	GT	GT	GT	GT	GT
intron	11559338	ATTTT	ATTTTT	ATTTTT	ATTTTT	ATTTTT	ATTTTT

For Review Only

	intron	11559186	G	C	C	C	C	C
	frameshift	11559057	AA	AATA	AATA	AATA	AATA	AATA
	intron	11558621	A	AG	AG	AG	AG	AG
	intron	11558592	AGG	AGGG	AGGG	AGGG	AGGG	AGGG
	splice region	11558512	T	TG	TG	TG	TG	TG
	intron	11558440	TC	TCC	TCC	TCC	TCC	TCC
	intron	11558340	ACC	ACCC	ACCC	ACCC	ACCC	ACCC
	intron	11558249	AA	AGTA	ATA	ATA	ATA	ATA
	intron	11558248	A	AG	AG	AG	AG	AG
	intron	11558183	T	TGG	TGG	TGG	TGG	TGG
	intron	11557980	A	AG	AG	AG	AG	AG
	frameshift	11557874	CT	CTT	CTT	CTT	CTT	CTT
	frameshift	11557770	GCCC	GCCCC	GCCCC	GCCCC	GCCCC	GCCCC
	3' UTR	11557468	ACTC	ACTCTC	ACTCTC	ACTCTC	ACTCTC	ACTCTC
	downstream	11555916	A	T	T	T	T	T
Vradi11g00000623	upstream	6179544	GAAAA	GAAAAA	GAAAAA	GAAAAA	GAAAAA	GAAAAA
	intron	6183762	TGG	TG	TG	TG	TG	TG
	intron	6183843	TCC	TC	TC	TC	TC	TC
	intron	6183928	ATTT	ATTTT	ATTTT	ATTTT	ATTTT	ATTTT
	downstream	6184281	GTTT	GTTTT	GTTTT	GTTTT	GTTTT	GTTTT
	downstream	6184324	ATT	ATTT	ATTT	ATTT	ATTT	ATTT
	downstream	6184571	CTTTTTTT	CTTTTTT	CTTTTTT	CTTTTTT	CTTTTTT	CTTTTTT

For Review Only
